# Recovery of Previously Uncultured Bacterial Genera from Three Mediterranean Sponges

**DOI:** 10.1007/s10126-017-9766-4

**Published:** 2017-07-10

**Authors:** Dennis Versluis, Kyle McPherson, Mark W. J. van Passel, Hauke Smidt, Detmer Sipkema

**Affiliations:** 1 Laboratory of Microbiology, Wageningen University & Research, Stippeneng 4, 6708 WE Wageningen, The Netherlands; 2 National Institute for Public Health and the Environment, Bilthoven, The Netherlands

**Keywords:** Antibiotic resistance, Sponge bacteria, Cultivation, Environmental resistance

## Abstract

Sponges often harbour a dense and diverse microbial community. Presently, a large discrepancy exists between the cultivable bacterial fraction from sponges and the community in its natural environment. Here, we aimed to acquire additional insights into cultivability of (previously uncultured) bacteria from three sponge species, namely *Aplysina aerophoba*, *Corticium candelabrum* and *Petrosia ficiformis*, by studying bacterial growth on five media in the form of 60 communities scraped from plates without antibiotics, as well as in the form of individual isolates that were grown on these media supplemented with antibiotics. We applied (double-)barcoded 16S ribosomal RNA (rRNA) gene amplicon sequencing for species identification. We show that previously uncultured bacteria can be cultivated using conventional plating and that application of antibiotics in the media can serve to capture a greater bacterial diversity. Moreover, we present criteria to address an important caveat of the plate scraping method whereby bacteria may be detected that did not actually grow. Fourteen out of 27 cultivated novel taxa (<95% identity of the 16S rRNA gene amplicon to reported species) belong to *Actinobacteria*, which indicates the presence of a large untapped reservoir of bioactive compounds. Three *Flavobacteriaceae* spp. were isolated that potentially constitute two new genera and one new species.

**Electronic supplementary material:**

The online version of this article (doi:10.1007/s10126-017-9766-4) contains supplementary material, which is available to authorized users.

## Introduction

Sponges arose more than 600 million years ago and have been consistent members of benthic communities ever since (Yin et al. 2015). Their diet consists of organic debris and plankton collected from the surrounding water through filter feeding. Even though sponges actively feed on microorganisms, they often simultaneously harbour dense and diverse microbial communities that co-exist with the host in a mutualistic relationship, and together are referred to as the sponge holobiont (Li et al. 2014).

Sponges are generally distinguished according to the bacterial cell density in their tissues where they either have a bacterial concentration within the range of surrounding seawater (low-microbial-abundance sponges), or the bacterial concentration is increased by two to four orders of magnitude (high-microbial-abundance sponges) (Hentschel et al. 2006). A comprehensive survey of publicly available 16S ribosomal RNA (rRNA) gene sequences of sponge-associated bacteria showed that, overall (in descending order of relative abundance) *Proteobacteria*, *Actinobacteria, Bacteroidetes, Chloroflexi* and *Firmicutes* are the dominant bacterial phyla (Webster and Taylor 2012). Another study that screened >12 million 16S rRNA gene pyrotags from 649 non-sponge marine environments found that 96 out of 173 previously described sponge-specific clusters could still only be retrieved from sponges (Simister et al. 2012; Taylor et al. 2013). The taxa that putatively remained sponge-specific comprised *Acidobacteria*, *Actinobacteria*, *Chloroflexi*, *Cyanobacteria*, *Gemmatimonadetes* and *Proteobacteria*, and the fact that they are predominantly obtained via molecular studies highlights the need to improve cultivation. There is great variation between sponge-associated microbial communities, and remarkably, bacterial communities seem to be largely host species-specific, with only few bacterial lineages shared between sponge species (Schmitt et al. 2012; Lee et al. 2011; Easson and Thacker 2014). Furthermore, host species-specific bacteriomes were found to be relatively stable in time (Hardoim and Costa 2014) and over wide bathymetric and geographic gradients (Reveillaud et al. 2014). Sponge-specific bacteria are of particular interest in the study of the complex host-symbiont relationship since they may represent the oldest animal-microbe symbioses on Earth (McFall-Ngai et al. 2013).

However, little is known regarding the metabolic functions and possible host-microbe interactions of sponge-associated bacteria. Functional roles that have been ascribed to sponge bacteria are carbon fixation (Thacker 2005), nitrogen cycling (Hoffmann et al. 2009; Taylor et al. 2007) and host defence (Hoppers et al. 2014; Mollica et al. 2012; Proksch 1994). Host defence is suggested in most cases to be the result of microbe-derived bioactive compounds, and in the last decade, their potential pharmaceutical application has sparked numerous studies (Mehbub et al. 2014; Indraningrat et al. 2016). The classical non-ribosomal polyketide synthethase (NRPS) and polyketide synthase (PKS) pathways responsible for secondary metabolite production in soil microbes have also been found in sponge bacteria, including novel variations that appear to be restricted to symbiotic bacteria (Manivasagan et al. 2014; Noro et al. 2012; Piel 2010). Still, bioactive compounds from sponge-associated bacteria are rather unexplored, a phenomenon that can be attributed to sponge-associated bacteria being difficult to culture.

Cultivation of sponge-associated bacteria is desirable in order to obtain a comprehensive understanding of their biology and ecological role (Alain and Querellou 2009). However, there is still a large discrepancy between the cultivable bacterial fraction from sponges and the community in its natural environment (Montalvo et al. 2014; Sipkema et al. 2011). To address this gap between presence in the natural environment and cultivation, we extensively studied the cultivability of sponge-associated bacteria from three Mediterranean high-microbial-abundance sponges, namely *Aplysina aerophoba, Petrosia ficiformis* and *Corticium candelabrum*. We collected total colony material obtained under different cultivation conditions, and performed community analysis via 16S rRNA gene amplicon sequencing. In addition, colonies were picked from media supplemented with different antibiotics in an effort to select for different bacterial species. We, for the first time, applied double-barcoded 16S rRNA gene amplicon sequencing for identification of individual bacterial colonies.

## Materials and Methods

### Overview of the Experiment

**Fig. 1:**
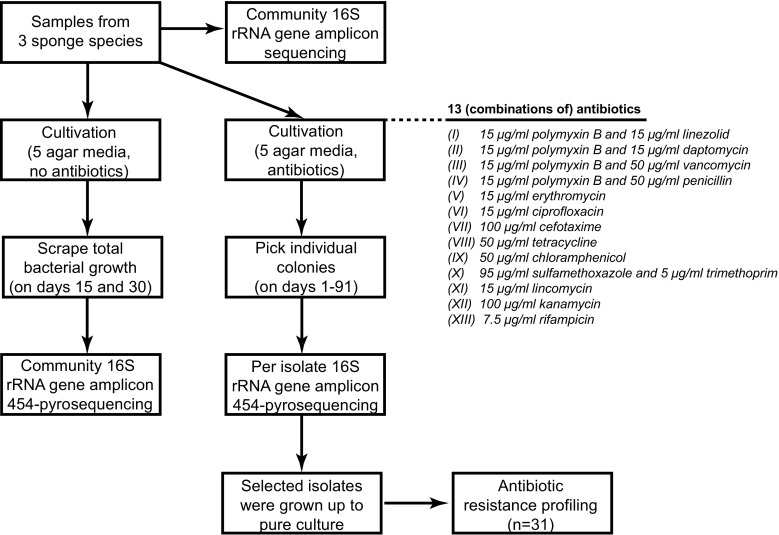
Flow diagram of the experiment. Bacterial diversity in samples from three different sponges (*A. aerophoba*, *P. ficiformis* and *C. candelabrum*) was compared to their cultivable fraction. The cultivable bacterial fraction was studied on five agar media in the form of 60 communities scraped from plates without antibiotics, and in the form of individual isolates picked from these media supplemented with 13 (combinations of) different antibiotics. Sponge samples were inoculated directly onto agar and on filter membranes on top of the agar. Resistance profiles were determined of 31 different isolates that were grown up to pure culture

The cultivable bacterial fraction from *A. aerophoba, P. ficiformis* and *C. candelabrum* was studied on five media in the form of 60 communities scraped from plates without antibiotics, and in the form of individual isolates picked from these media supplemented with 13 (combinations of) different antibiotics (Fig. 1). Agar media were supplemented with two antibiotics if they predominantly affect the growth of either Gram-negative or Gram-positive bacteria. We observed many more colonies on media without antibiotics; therefore, for practical reasons, we decided to use the scraping method to analyse bacterial growth on these media.

### Sample Collection

Tissue samples were collected from one individual per sponge species. Samples from *P. ficiformis* (Pf1) and *C. candelabrum* (CC1) were collected on 5 June 2008 from Punta de Santa Ana, Blanes (41° 40′ 33″ N, 2° 48′ 10″E) at 10 m depth and identified by M.J. Uriz. *A. aerophoba* (AA3) samples were collected on 15 January 2012 from Cala Montgo (near L’Escala) (42° 06′ 52.20″ N, 03° 10′ 06.52″ E) at 12 m depth and identified by D. Sipkema. Specimens were brought to the surface in plastic ziplock bags. The sponge samples were rinsed three times with sterile artificial seawater (ASW, 33 g/l synthetic sea salt [Instant Ocean Reef Crystals, Aquarium Systems, Sarrebourg, France]) and were cut into pieces of ~0.1 cm^3^. Half of the pieces was stored in 100% ethanol, while the other half was homogenized with mortar and pestle, and two tissue volumes of sterile artificial seawater were added to obtain a homogeneous cell suspension. This suspension was aliquotted in 1.2 ml and mixed with 0.6 ml 50% sterile glycerol in ASW before storage at −80 °C.

### Cultivation Conditions

#### Isolation of Bacteria on Media with Antibiotics

For each sponge, material from the original glycerol stocks was 10-fold diluted in ASW. Subsequently, one agar plate was inoculated with 75 μl diluted sample for each combination of five different agar media and 13 different selections of antibiotics (amounting to 65 agar plates per sponge). Out of the 75-μl sample per plate, 50 μl was inoculated in contact with agar whereas the other 25 μl was inoculated on a 0.2 μm GTTP isopore membrane filter (Merck Millipore, Billerica, USA) that was placed on top of the agar. The following five media were used: (I) 37.40 g/l marine broth 2216 (Difco, Detroit, USA) in Milli-Q; (II) 3.74 g/l marine broth 2216 (Difco) in Milli-Q; (III) 22 g/l BBL Mueller-Hinton broth (BD, Franklin Lakes, USA) in ASW; (IV) 2.2 g/l BBL Mueller-Hinton broth (BD) in ASW and (V) 1 g/l porcine stomach mucin (Sigma) in ASW. All media contained 15 g/l noble agar (Sigma-Aldrich, St. Louis, USA). Media III to V were supplemented with 1 ml/l trace metal solution (Olson and McCarthy 2005), 1 ml/l phosphate solution (Olson and McCarthy 2005) and 1 ml/l vitamin solution (BME Vitamins, Sigma-Aldrich), with the pH adjusted to 7.7 before autoclaving. Solutions of phosphate, vitamins and antibiotics were filter-sterilized (0.2 μm) and added after autoclaving in order to prevent precipitation or inactivation. The following 13 (combinations of) antibiotics were added: (I) 15 μg/ml polymyxin B and 15 μg/ml linezolid; (II) 15 μg/ml polymyxin B and 15 μg/ml daptomycin; (III) 15 μg/ml polymyxin B and 50 μg/ml vancomycin; (IV) 15 μg/ml polymyxin B and 50 μg/ml penicillin; (V) 15 μg/ml erythromycin; (VI) 15 μg/ml ciprofloxacin; (VII) 100 μg/ml cefotaxime; (VIII) 50 μg/ml tetracycline; (IX) 50 μg/ml chloramphenicol; (X) 95 μg/ml sulfamethoxazole and 5 μg/ml trimethoprim; (XI) 15 μg/ml lincomycin; (XII) 100 μg/ml kanamycin and (XIII) 7.5 μg/ml rifampicin. Plates were incubated in the dark at 20 °C for 3 months. Colonies were picked twice per week in the first month and subsequently once per week. At most, three colonies of the same morphology were picked per plate. Colonies were transferred to a fresh plate, and colony material from these plates was used for preparation of glycerol stocks. Colonies appearing on filters were immediately stored in glycerol. Glycerol stocks were prepared by transferring colony material to 96-well plates containing 25% glycerol in ASW, and stored at −80 °C. In addition, colony material was transferred to 96-well plates containing nuclease-free water (Promega, Madison, USA) to be used as direct template for 16S rRNA gene PCR.

#### Bacterial Growth on Media Without Antibiotics

Material from the original glycerol stocks of *A. aerophoba*, *P. ficiformis* and *C. candelabrum* was respectively 100-, 10- and 2-fold diluted in ASW based on prior CFU counts, and subsequently plated on six plates of each of the five media described previously. Seventy-five microlitres of diluted material was inoculated on each plate (50 μl in contact with agar and 25 μl on a filter membrane on top of the agar). The plates were stored at 20 °C in the dark. Colony material from each combination of media and sponges was harvested 15 days (3 plates) and 30 days (the remaining three plates) post incubation by plate washing with the help of an L-shaped spreader. Colony material on top of agar and filter membranes was collected separately in 3 ml phosphate-buffered saline (PBS, 137 mM NaCl, 2.7 mM KCl, 10 mM Na_2_HPO_4_, KH_2_PO_4_) from which 2.5 ml was stored for genomic DNA isolation at −20 °C. The rest was mixed with 0.5 ml 50% glycerol (in ASW) and stored at −80 °C for cultivation.

### DNA Extraction and 16S rRNA Gene Amplicon Sequencing

#### DNA Extraction

The DNeasy Blood & Tissue Kit (Qiagen, Venlo, Netherlands) was used to extract total DNA from 0.4 cm^3^ of sponge tissue as well as from colony material stored in PBS (the fractions that were washed from the plates without antibiotics), according to the manufacturer’s protocol for animal tissue. The DNA concentration was determined by NanoDrop (Thermo Fisher Scientific, Bremen, Germany). DNA samples were stored at −20 °C in AE buffer.

#### Barcoded 16S rRNA Gene Amplicon Pyrosequencing

Barcoded 16S rRNA gene amplicon 454-pyrosequencing was done (I) to analyse bacterial communities present in the sponge samples, (II) to identify bacterial colonies that were picked from media containing antibiotics and (III) to analyse bacterial communities retrieved by scraping from media without antibiotics. PCR was performed to amplify an approximately 311-bp fragment comprising the V1 and V2 regions of the 16S rRNA gene. For this purpose, the extracted DNA of the samples was diluted to 10–20 ng/μl. In case of identification of bacterial colonies, cell material in nuclease-free water served as template. The composite forward primer consisted of titanium sequencing adaptor A, a barcode (Hamady et al. 2008) and degenerate primer 27F-DegS (van den Bogert et al. 2011) (Supplementary Table S1). Reverse priming was done by an equimolar mixture of primers 338R-I and 338R-II (Daims et al. 1999), each attached to titanium sequencing adaptor B. For the identification of individually picked colonies, a barcode was also included in the reverse primer (for each colony, a unique combination of barcodes was used). PCR conditions and library preparation are described in the Supplementary Materials and Methods. Five libraries were sent for sequencing that contained samples particular to this study (Supplementary Table S1).

### Demultiplexing, Quality-Filtering, Denoising and Taxonomy Assignment of 454-Pyrosequencing Data

454-Pyrosequencing reads were processed with QIIME (Caporaso et al. 2010). Firstly, each library was demultiplexed according to the forward primer barcodes. Reads were retained if the average Phred quality score was >25, no homopolymer stretches >6 nucleotides were present, the read length was ≥200 bp and no mismatches were present in the 5′-end primers. For 454-pyrosequencing reads that were double-barcoded (i.e. those generated from picked colonies), an additional demultiplexing step based on the reverse barcode was performed. Subsequently, reads were denoised by Acacia (Bragg et al. 2012) using default settings. Next, open reference operational taxonomic unit (OTU) picking and taxonomic assignment was performed by UCLUST (Edgar 2010) based on a 97% identity threshold, and the representative sequences of the 97% identity clustered SILVA 111 database as a reference. The representative sequence from each OTU was picked, which is the centroid that was used for OTU picking. Potentially chimeric OTUs were identified using ChimeraSlayer and the downloadable Greengenes core_set_aligned.fasta.imputed, and were excluded from downstream analysis. Finally, OTU tables were generated that tabulate which OTUs, orders or phyla were detected in each sample, and by how many reads.

### Regrowth, Identification and Resistance Profiling of Picked Isolates

#### Regrowth from Glycerol Stocks and Identification

OTUs were selected for regrowth from the glycerol stocks from picked colonies if the reads assigned to the OTU in at least one case represented >50% of reads that were assigned to that colony. The strains belonging to the OTUs were regrown on their original isolation media, and subsequently maintained on marine agar if this medium could support their growth. Bacterial cultures were passaged until pure, and their identity was confirmed by 16S rRNA gene Sanger sequencing using the 27F and 1492R general bacterial primers (Jiang et al. 2006). One reaction mixture contained 10 μl 5× GoTaq reaction buffer (Promega), 1 μl 10 mM dNTPs (Promega), 0.5 μl GoTaq® DNA polymerase (5u/μl, Promega), 1 μl 10 μM upstream primer, 1 μl 10 μM downstream primer, 1 μl template (briefly boiled bacterial biomass in water) and 35.5 μl nuclease-free water (Promega). The PCR program consisted of initial denaturation at 95 °C for 5 min; 30 cycles of denaturation at 95 °C for 30 s, annealing at 52 °C for 40 s and extension at 72 °C for 90 s; and final extension at 72 °C for 7 min. PCR products were sent to GATC Biotech with sequencing primers 27F, 1492R or 907R (Schauer et al. 2000). Read ends were trimmed with DNA Baser version 3.5.4.2 until there were 99% good bases (quality value >21) in a 20-base window.

The V1–V2 region of the 16S rRNA gene of each isolate was Sanger sequenced to facilitate alignment with the 454-pyrosequencing reads. Furthermore, in order to improve taxonomic resolution, the 16S rRNA gene of each isolate was Sanger sequenced to a length of ≥800 bp. The Sanger read pertaining to an isolate needed to be >97% identical to the representative read of the target OTU for it to be considered the corresponding strain. Three attempts for cultivation up to pure culture were made per selected OTU, where possible, from distinct wells in the 96-well plates.

#### Resistance Profiling of Pure Isolates

Isolates were tested for resistance to all antibiotics used in the cultivation experiment (at identical concentrations), as well as for resistance to ampicillin (50 μg/ml) and imipenem (10 μg/ml). The isolates were pregrown in liquid culture (same media but without noble agar), and subsequently inoculated on agar media supplemented with antibiotics. Antibiotic resistance was evaluated 3, 4 and 8 days post inoculation while using growth on media without antibiotics as a reference. Bacteria were categorized to be ‘resistant’, ‘intermediately resistant’, or ‘susceptible’.

### Analyses and Visualization of Bacterial Diversity

#### Diversity and Clustering Analyses of Bacterial Communities

For sponge bacterial communities, rarefaction curves were calculated with QIIME scripts. Hierarchical clustering was performed using R package ‘Vegan’ (Dixon 2003) based on OTU-level relative abundance data obtained from bacterial communities in sponge tissues as well as those scraped from agar plates. Canonical (constrained) correspondence analysis (CCA) as implemented in Canoco 5 (Šmilauer and Lepš 2014) was used to investigate which experimental variables best explain the variation in species composition regarding OTU-level relative abundance data obtained from the bacterial communities scraped from agar plates. SIMPER (Clarke and Gorley 2006) was used with respect to square-rooted OTU-level relative abundance data to break down the contribution of each OTU to the observed dissimilarity between sample groups. ARB (Ludwig et al. 2004) was used to construct a 16S rRNA gene-based phylogenetic tree of bacterial isolates (>800-bp sequences were obtained by Sanger sequencing), their closest type strain (based on blastn, bitscore sorted) and the nearest neighbour in the Silva guide tree (release 115). The analyses are described in detail in the Supplementary Materials and Methods.

### Nucleotide Sequence Accession Numbers

The partial 16S rRNA gene sequences of pure isolates were deposited under accession numbers KP769416 to KP769446. The 16S rRNA gene amplicon sequences were deposited in the ENA SRA database under accession number PRJEB4784 (Supplementary Table S1).

## Results

### Bacterial Diversity in Sponge Samples and Their Cultivable Fraction

After quality checking, 454-pyrosequencing sequence data yielded a total of 716,939 reads that clustered into 2282 bacterial OTUs. Firstly, 14,670 reads were assigned to the three sponge samples. Secondly, 41,548 reads were assigned to 542 out of 679 picked colonies from plates with antibiotics, a discrepancy that can largely be explained by the fact that 82 red colonies were picked to which no OTU could be assigned. These red colonies, which were picked from plates inoculated with *A. aerophoba* sample, were found to belong to the *Penicillium* genus (data not shown). Five hundred fifty-six (81.9%) colonies were picked from *A. aerophoba*, 95 (14.0%) colonies were picked from *P. ficiformis* and 28 (4.1%) colonies were picked from *C. candelabrum*. Thirdly, 660,721 reads were assigned to the 60 bacterial communities collected by scraping from agar media without antibiotics. In total, 2844, 571 and 334 colonies were scraped from agar media inoculated with samples from *A. aerophoba*, *C. candelabrum* and *P. ficiformis*, respectively. Most colonies were scraped from mucin agar (*n* = 1097), followed by 10× diluted Mueller-Hinton agar (*n* = 808), marine agar (*n* = 745), 10× diluted marine agar (*n* = 741) and Mueller-Hinton agar (*n* = 358). Supplementary Table S2 shows the number of colonies that were picked or scraped in this experiment subdivided per sponge species according to the agar media that were used.

**Fig. 2:**
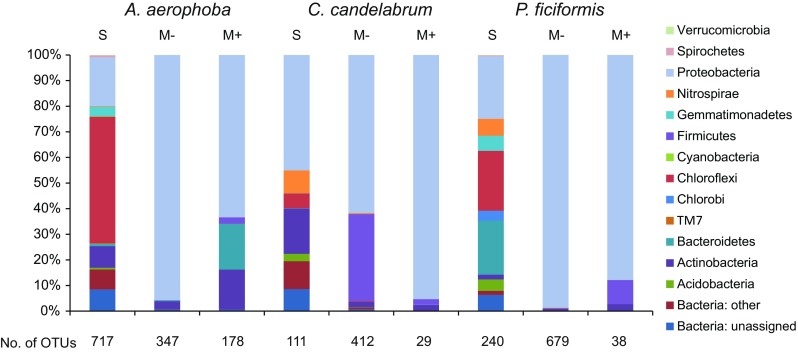
Phyla that were detected in the sponge samples (S), on media without antibiotics (M−) and on media with antibiotics (M+). Phyla with a relative abundance <0.1% are not shown. The relative abundance values are based on the combined reads of the different experimental groups

Shannon indices for sponge samples indicated that *C. candelabrum* (H′= 3.96 ± 0.01 [s.d.]) contained the least diverse bacteriome when compared to *A. aerophoba* (H′= 5.29 ± 0.04 [s.d.]) and *P. ficiformis* (H′= 4.54). *Proteobacteria, Nitrospirae*, *Chloroflexi*, *Bacteroidetes*, *Actinobacteria* and *Acidobacteria* were present in all three sponges at >0.1% relative abundance (Fig. 2). *A. aerophoba* and *C. candelabrum* were dominated by *Chloroflexi* and *Proteobacteri*a, respectively (>40% of the bacteria). The bacteriome profile of *P. ficiformis* was characterized by high relative abundances of *Proteobacteria*, *Chloroflexi* and *Bacteroidetes*. It is noteworthy that in the bacteriome of *P. ficiformis* >20% of the bacteria belonged to *Bacteroidetes* whereas in the other two sponges their relative abundance was <1%. Rarefaction analysis demonstrated that the current sequencing depth did not capture the complete bacterial diversity in the sponge samples (Supplementary Fig. S1).

### Bacteria Isolated in the Absence of Antibiotics

Bacterial communities on the five agar media without antibiotics were dominated by *Proteobacteria* (>50% relative abundance) for the three sponge species investigated (Fig. 2). High proteobacterial abundance was in most cases attributed to OTU denovo1749 (>50% relative abundance in 44 out of 60 scraped fractions), an *α-proteobacterium* affiliated to the *Pseudovibrio* genus. Notably, OTU1749 was not detected in the sponge samples. On plates inoculated with *C. candelabrum* samples, also *Firmicutes* were relatively abundant with 34% of the total reads.

**Fig. 3:**
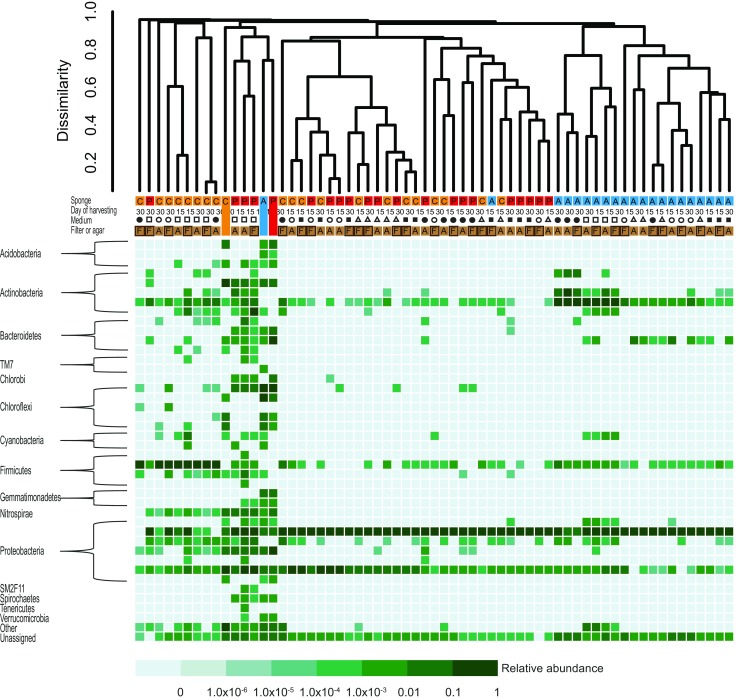
Hierarchical clustering using Bray-Curtis distance based on 16S rRNA gene amplicons generated from three sponge samples (*A. aerophoba*, *P*. *ficiformis* and *C. candelabrum*) and corresponding communities retrieved by scraping from agar media. Bacterial communities were investigated on marine agar (*squares*), marine agar 10-fold diluted (*open squares*), Mueller-Hinton agar (*circles*), Mueller-Hinton 10-fold diluted (*open circles*) and mucin agar (*open triangles*).Samples were either inoculated in direct contact with agar (*A*) or on top of a filter (*F*), and were harvested 15 and 30 days post inoculation. The sponge samples (inocula) are indicated with continuous colour bars. Hierarchical clustering was performed at the OTU level (97% identity clusters). The *heatmap* corresponds to relative abundance values of order-level phylogenetic groups (>0.01% relative abundance in at least one sample)

Hierarchical clustering was performed to investigate dissimilarity at the OTU level between communities present in the sponge samples and those present on media without antibiotics (Fig. 3). *A. aerophoba*-derived communities retrieved by scraping from plates and filters formed one large cluster because all samples featured a relatively high abundance of members belonging to the genus *Ruegeria* (OTU HE574879) (Supplementary Table S3). Within this cluster, as a general rule, higher similarity was observed between samples that were retrieved from the same medium (Fig. 3). Scraped communities derived from *P. ficiformis* and *C. candelabrum* formed two mixed branches that each contain >10 samples. Separate clustering can be attributed to a relatively high abundance of a subset of six specific OTUs in one of the two branches. These signature OTUs constitute *Microbulbifer* spp*.* (five OTUs) and a *Pseudovibrio* sp. (one OTU). Samples within both of these branches showed further grouping according to cultivation medium. The remaining samples in the dendrogram are the original sponge communities and 12 communities derived from *P. ficiformis* and *C. candelabrum* that were highly dissimilar to each other. All except one of these 12 communities were composed of <10 visible colonies.

CCA of square-rooted OTU-level relative abundance data from the scraped bacterial communities indicated that the explanatory variables medium I (marine agar), sponge *C. candelabrum*, sponge *P. ficiformis*, sponge *A. aerophoba*, medium II (marine agar, 10-fold diluted nutrients) and medium III (Mueller-Hinton agar) could explain respectively 5.1% (*p* = 0.002), 5.0% (*p* = 0.002), 3.8% (*p* = 0.002), 3.8% (*p* = 0.002), 3.2% (*p* = 0.002) and 2.8% (*p* = 0.002) of the total variation in diversity using forward selection (Supplementary Fig. S2). Most variation could not be accounted for by the explanatory variables that were evaluated. Similarity percentage (SIMPER) analyses indicated that most dissimilarity in terms of bacterial growth scraped from each sponge species could be explained by different levels of average relative abundance of OTU denovo1749 (*Pseudovibrio* sp.) (Supplementary Table S4). In scraped communities derived from *P. ficiformis*, the average relative abundance of OTU denovo1749 was 89%, while the corresponding values for *A. aerophoba* and *C. candelabrum* were 77 and 63%, respectively. Other top contributors to dissimilarity were OTU HE574879 (*Ruegeria* sp.) that was present with higher relative abundance, and OTU GQ118701 (*Microbulbifer* sp.) that was present with lower relative abundance, on plates inoculated with material from *A. aerophoba*, as compared to plates inoculated with samples from the other two sponges. In addition, OTU JN579972 (*Bacillus* sp.) contributed to the dissimilarity by a higher relative abundance on plates inoculated with *C. candelabrum*. Comparison by SIMPER of bacterial communities retrieved directly from agar and those obtained on filter membranes showed that there are no bacterial taxa that highly favour one growth surface over the other i.e. no taxon occurred with great differences in relative abundance that also substantially (>2%) contributed to the dissimilarity.

Bacterial growth on the scraped plates was investigated for novel cultivable bacteria. Seventy-nine novel OTUs (≤95% identity of the representative read to the 16S rRNA gene of the closest type strain) were identified. However, 13 of these 79 OTUs had a lower relative abundance in the scraped communities as compared to the inoculum (sponge sample) (Supplementary Fig. S3). We applied three criteria to conclude whether bacteria grew: (I) OTUs needed to increase in relative abundance as compared to the inoculum, (II) OTUs needed to have a relative abundance of ≥0.2% and (III) the scraped community required ≥20 visible colonies.

Adopting these criteria, we retained as many as 27 novel cultivable OTUs from the scraped communities (Table 1). At the phylum level, the 27 OTUs belong to the *Proteobacteria* (10 OTUs [8 α- and 2 γ-]), *Firmicutes* (2 OTUs), *Bacteroidetes* (1 OTU) and *Actinobacteria* (14 OTUs). Most (19/27) novel OTUs grew on Mueller-Hinton-based agar media. Five of the novel OTUs (AJ347026, denovo528, denovo574, GU118526 and denovo98) were detected in the inoculum as well (Supplementary Table S3).

**Table 1:** OTUs were derived from 16S rRNA gene sequences generated from bacterial communities retrieved from agar media inoculated with samples from three sponges (*A. aerophoba*, *P. ficiformis* and *C. candelabrum*) on different agar media

OTU ID	Phylum	Closest type strain	% identity	No. of samples	Rel. abundance (%)	Sponge	Medium
AJ347026	Actinobacteria	*Euzebya tangerina*	82.24	1	0.91	*P. ficiformis*	Ma10
denovo528	Proteobacteria	*Nisaea nitritireducens*	85.34	1	0.43	*P. ficiformis*	Ma10
denovo2029	Actinobacteria	*Luteimicrobium subarcticum*	87.58	1	0.31	*A. aerophoba*	MH
denovo1961	Actinobacteria	*Brachybacterium paraconglomeratum*	88.25	3	≤1.68	*A. aerophoba*	MH, Ma10
denovo574	Proteobacteria	*Haliea mediterranea*	88.66	1	0.36	*P. ficiformis*	Ma10
denovo927	Firmicutes	*Bacillus horikoshii*	89.34	2	≤0.63	*A. aerophoba*	MH
denovo639	Actinobacteria	*Brachybacterium paraconglomeratum*	89.74	1	0.31	*A. aerophoba*	MH
denovo1500	Actinobacteria	*Rhodococcus globerulus*	89.91	1	0.32	*A. aerophoba*	MH
GU118526	Proteobacteria	*Nisaea denitrificans*	90.38	1	3.04	*P. ficiformis*	Ma10
denovo1249	Proteobacteria	*Pseudovibrio ascidiaceicola*	91.26	1	0.20	*P. ficiformis*	MH10
denovo98	Proteobacteria	*Albimonas donghaensis*	92.50	1	0.24	*P. ficiformis*	Ma10
denovo1070	Firmicutes	*Bacillus horikoshii*	93.06	1	0.31	*A. aerophoba*	MH
denovo767	Actinobacteria	*Nocardia ninae*	93.09	2	≤0.32	*A. aerophoba*	MH
denovo1341	Actinobacteria	*Brachybacterium paraconglomeratum*	93.53	1	0.32	*A. aerophoba*	MH
denovo1132	Actinobacteria	*Janibacter melonis*	93.61	1	0.21	*A. aerophoba*	MH
denovo1129	Actinobacteria	*Nocardia globerula*	94.08	3	≤0.37	*A. aerophoba*	MH
denovo1096	Actinobacteria	*Pseudonocardia dioxanivorans CB1190*	94.12	2	≤0.63	*A. aerophoba*	MH
denovo1923	Actinobacteria	*Knoellia locipacati*	94.23	1	0.51	*A. aerophoba*	Ma10
denovo1146	Proteobacteria	*Microbulbifer epialgicus*	94.30	3	≤0.72	All three sponges	MH10, Ma
denovo1414	Proteobacteria	*Sphingomonas desiccabilis*	94.33	1	0.24	*A. aerophoba*	Ma10
denovo1558	Bacteroidetes	*Postechiella marina*	94.50	2	≤1.50	*A. aerophoba*	MH10, Mu
denovo1446	Actinobacteria	*Brachybacterium paraconglomeratum*	94.52	1	0.32	*A. aerophoba*	MH
denovo103	Proteobacteria	*Pseudovibrio denitrificans*	94.68	1	0.21	*A. aerophoba*	Ma10
denovo706	Actinobacteria	*Rhodococcus globerulus*	94.70	1	0.31	*A. aerophoba*	MH
denovo1412	Actinobacteria	*Brevibacterium antiquum*	94.90	2	≤1.05	*A. aerophoba*	MH
denovo1479	Proteobacteria	*Ruegeria atlantica*	94.96	4	≤2.74	*A. aerophoba*	MH10, Ma
denovo953	Proteobacteria	*Ruegeria halocynthiae*	95.00	4	≤0.36	*A. aerophoba*	MH10, MH

Amongst all OTUs, 27 were detected that showed ≤95% identity (blastn) to the closest type strain. These OTUs all increased in relative abundance as compared to the inoculum, had a relative abundance of ≥0.2% and were detected in a scraped community of ≥20 colonies, in at least one scraped community. The 27 OTUs are listed together with the closest type strain, the number of samples in which they were detected with the aforementioned criteria, the maximum relative abundance achieved for this OTU in these samples, the inoculum (sponge species) and the media on which they were detected

*MH* Mueller-Hinton agar, *MH10* 10-fold diluted Mueller-Hinton agar, *Mu* mucin agar, *Ma* marine agar, *Ma10* 10-fold diluted marine agar

### Bacteria Isolated in the Presence of Antibiotics

Bacterial colonies were identified by double-barcoded pyrosequencing. Reads were allocated to 542 out of 679 picked colonies, and amongst these colonies a total of 211 OTUs were detected. There was a high number of OTUs that were detected by only one or a few reads, and in almost all cases these comprised the minority of reads assigned to a colony (Supplementary Fig. S4). An OTU was only assigned if it represented >50% of reads obtained for that colony, which resulted in 47 OTUs being assigned to 425 picked colonies (Supplementary Fig. S5). None of these 47 OTUs belonged to sponge-specific clusters (Simister et al. 2012). On average, a similar number of OTUs were assigned to colonies picked directly from agar media (2.56 ± 2.10 [s.d]) as compared to those picked from filters on top of agar media (2.33 ± 1.80 [s.d.]). For 31 of 47 OTUs that were assigned to colonies, a representative isolate was successfully regrown from the glycerol stocks up to pure culture. OTU denovo1749 (*Pseudovibrio* sp.) was assigned to isolates obtained from all three sponge species. From *A. aerophoba* and *P. ficiformis*, a representative isolate belonging to this OTU was successfully cultured up to pure culture. Isolates may be different strains if they belong to the same OTU but are obtained from different sponges, and hence, both strains were included in subsequent analyses.

**Fig. 4:**
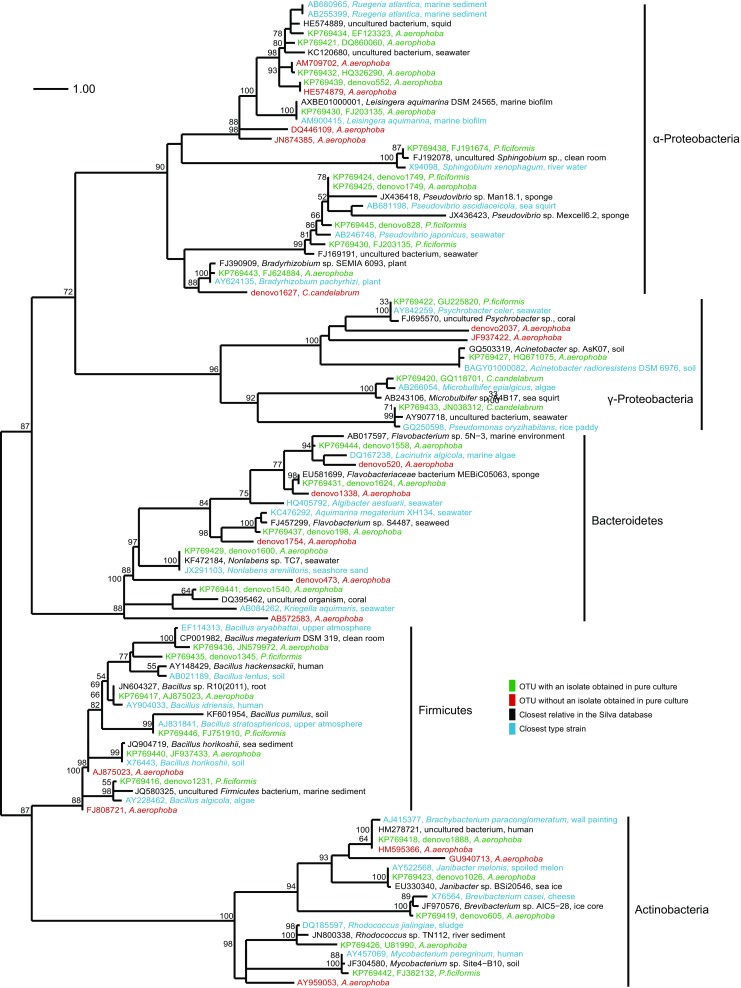
Phylogenetic tree based on 16S rRNA gene sequence similarity (>800-bp sequences) showing sponge bacteria cultured up to pure culture that were isolated agar media containing antibiotics (*green*), their closest type strain (based on blastn, *blue*) and the nearest neighbour in the Silva guide tree (*black*). The tree was constructed in ARB by maximum likelihood analysis using 1000 iterations of RAxML rapid bootstrapping. For tree calculation, highly variable positions (1–9) were excluded using the bacterial positional variability by parsimony filter, and non-overlapping regions were excluded with a custom filter (window of inclusion, positions 5331 to 26,803). For each strain, the accession number, full species name and isolation source are indicated. Bootstrap values <50 are not shown. The *horizontal bar* corresponds to one substitution per site. After tree creation, representative pyrosequencing reads of OTUs for which unsuccessful attempts were made to obtain a representative in pure culture (*red*) were added using “add species to existing tree” with ARB_Parsimony, applying similar filtering settings as those used for creation of the base tree. For these OTUs, the OTU name is stated, thereafter followed by the isolation source

For each of the 31 pure cultures, the 16S rRNA gene was sequenced to a length of at least 800 bp. A phylogenetic tree was constructed (Fig. 4) based on 16S rRNA gene sequences from the isolates we obtained in pure culture, their most closely related type strain and their closest neighbour in the Silva guide tree. These isolates belonged to *Firmicutes* (*n* = 5), *Actinobacteria* (*n* = 6), *Proteobacteria* (*n* = 15) and *Bacteroidetes* (*n* = 5). The most closely related type strains could be separated into those isolated from the marine environment (*n* = 15), and those isolated mostly from soil, air and plants (*n* = 12). A similar distribution was observed for the closest neighbours where 18 out of 27 sequences originated from the marine environment. Three isolates belonging to the family of *Flavobacteriaceae* were isolated that potentially constitute two new genera and one new species. These strains exhibited the highest 16S rRNA gene sequence identity to *Kriegella aquimaris* (denovo1540, 94.9% identity), *Gaetbulibacter marinus* (denovo1624, 95.6% identity) and *Lacinutrix algicola* (denovo1558, 96.7% identity).

Furthermore, each of the 31 pure cultures was investigated for resistance to the antibiotics used in the cultivation experiment, plus ampicillin and imipenem (Table 2). Resistance to tetracycline, polymyxin B and kanamycin was most frequent, whereas no single isolate was resistant to rifampicin or imipenem. Generally, if isolates were susceptible to a certain antibiotic, then the corresponding OTU was not detected on media supplemented with this antibiotic in the original cultivation experiment from which they were derived. Except for *Bradyrhizobium pachyrhizi* (OTU: FJ624884), isolates were always simultaneously resistant to both antibiotics belonging to the penicillin group of β-lactam antibiotics i.e. ampicillin and penicillin. Eleven Gram-negative bacteria were sensitive to vancomycin and/or daptomycin, even though these antibiotics primarily target Gram-positive bacteria (Table 2).

**Table 2:**
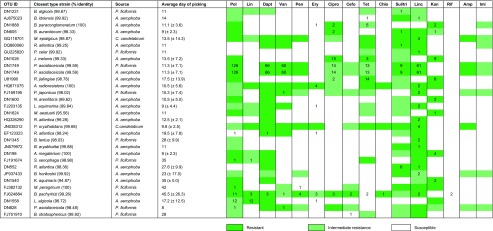
Resistance profiles of bacteria isolated from the sponges *A. aerophoba*, *P. ficiformis* and *C. candelabrum*

Bacterial identification was done by blastn of the 16S rRNA gene sequence (>800 bp) against a database of bacterial type strains (sequence identity values are shown). Numerical values indicate the number of times bacteria from the same OTU were detected on media supplemented with these antibiotics in the cultivation experiment from which they were derived. Note that no media were supplemented with ampicillin nor imipenem in the preceding cultivation experiment

Publicly available genomes were obtained for six bacteria of the same species as our isolates and investigated in terms of resistance gene composition (Supplementary Table S5) (Gibson et al. 2014). We detected putative β-lactam resistance genes in all tested genomes even though only *Acinetobacter radioresistens* was found to be resistant to a β-lactam antibiotic (penicillin). *A. radioresistens* was also resistant to chloramphenicol and erythromycin. In each of six publicly available *A. radioresistens* genomes, we detected genes encoding a chloramphenicol transferase, membrane fusion protein MacA and inner membrane protein MacB, which could provide resistance to the aforementioned antibiotics. In three genomes (from *Sphingobium xenophagum, Leisingera aquimarina* and *Bacillus stratosphericus*), MFS efflux pump genes were detected, which could explain why our corresponding isolates are tetracycline-resistant.

### Comparison of Bacterial Presence in Sponge Samples and their Cultivable Fraction

**Fig. 5:**
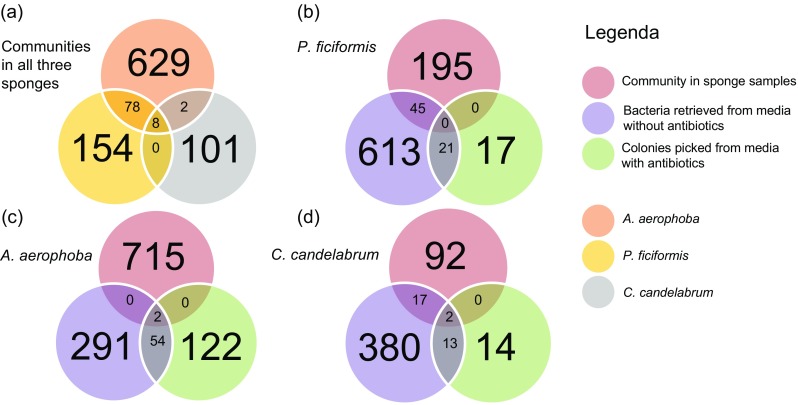
Venn diagrams that illustrate the relationship between OTUs that were detected in the three sponge samples, colonies picked from agar media supplemented with a wide variety of antibiotics and bacteria retrieved by scraping from agar media without antibiotics. **a** Comparison of the overlap in terms OTUs in the samples of the three different sponge species (*A. aerophoba, P. ficiformis* and *C. candelabrum*). **b**–**d** Comparison of the overlap in terms of OTUs between the sponge samples and their cultivable fractions

For *P. ficiformis*, 45 OTUs were detected in both the sponge sample and the corresponding scraped communities (Fig. 5), whereas for *C. candelabrum* the overlap constituted 19 OTUs. In the case of *A. aerophoba*, only two OTUs (GU940713 and EU803928) were retrieved in the scraped fraction, and those were classified at the genus level to *Propionibacterium* and *Halomonas*. Supplementary Table S6 provides taxonomic information regarding the OTUs that overlap for the different datasets. No OTUs were detected both in the sponge samples and on media supplemented with antibiotics, without them also being detected in the communities that were derived from media without antibiotics.

Eight OTUs were detected in the bacterial communities of the three sponge species (Fig. 5). In addition, 78 OTUs were shared exclusively between *A. aerophoba* and *P. ficiformis*, whereas very few OTUs were shared between either of these two sponges and *C. candelabrum* (two and zero OTUs, respectively). The highest number of OTUs was detected in the combined scraped communities, and this experimental group also constituted the bulk of the reads (92.2%) (Supplementary Table S3).

## Discussion

### Bacterial Profiles of the Sponge Hosts

In studying bacterial culturability, it is essential to know which bacterial species are present in the original samples. Therefore, the bacterial community profiles of the sponges studied here i.e. *A. aerophoba, C. candelabrum* and *P. ficiformis* were determined. In the bacteriome of *Aplysina* spp., the sponge-specific candidate phylum *Poribacteria* was originally discovered (Fieseler et al. 2004), of which just recently a member was genomically characterized after single-cell sorting (Siegl et al. 2011; Kamke et al. 2014). *Poribacteria* could not be detected in this study due to insufficient similarity of the 454-pyrosequencing forward primer to the 16S rRNA gene target. Only 8 out of 972 OTUs detected in the sponge samples were shared between all three sponges indicating that, as suggested by past studies (Schmitt et al. 2012; Naim et al. 2014), bacterial colonization is highly sponge species-specific.

### Bacterial Diversity Captured by Scraping Bacterial Biomass from Agar Media

Twenty-seven novel OTUs (defined as those for which the representative read has <95% identity to the 16S rRNA gene of the closest type strain) were cultivated, albeit at ≤3% relative abundance. Fourteen out of 27 novel OTUs belong to the phylum *Actinobacteria*, members of which are known producers of secondary metabolites (Manivasagan et al. 2014; Sanglier et al. 1993; Taylor et al. 2007). These strains are therefore particularly interesting for obtaining novel bioactive compounds with potential pharmaceutical applications. Five OTUs that met the criteria for growth were detected of which the 454-pyrosequencing read exhibited <89% sequence similarity with the closest type strain, and these strains (three *Actinobacteria* and two *Proteobacteria*) potentially belong to novel orders (Yarza et al. 2014). Three of these five OTUs (OTUs AJ347026, GU118536 and denovo528) were detected in bacterial biomass retrieved from agar media as well as in the original sponge samples, indicating a role in the sponge host. Consequently, they are of particular interest in the study of host-symbiont relations. OTU AJ347026 is especially relevant for these studies since it is highly novel (82.2% sequence similarity of the 454-pyrosequencing read to the 16S rRNA gene of the closest type strain), and it was previously assigned to a sponge-specific clade (Hentschel et al. 2002). OTU GU118536 was detected earlier in the coral *Montastraea faveolata* by cultivation-independent methods (Sunagawa et al. 2010). We detected no growth of novel *Chloroflexi*, *Nitrospirae* and *Bacteroidetes*, which are typically considered harder to cultivate by conventional plating methodologies such as those applied in this study. Our study is the first cultivation study for *C. candelabrum*, but nevertheless, most novel bacteria were detected in the cultivable fraction from *A. aerophoba*, warranting further investigation into those samples. It should be noted that since we did not obtain strains belonging to the novel OTUs (Table 1) in pure culture, there is the possibility that some are dependent on other strains for growth or some compounds from the sponges, and might therefore not be readily cultivable up to pure cultures by the methods applied here (West et al. 2007). It should also be noted that sponges filter thousands of litres of water per day (Vogel 1977), and as a result, it could be that some of the bacteria that we detected are not actually permanently associated to the sponge but merely transiently present. However, irrespective of these bacteria being permanently associated or not, our results show that these sponges are an accessible resource of bacterial taxa that may have the potential to produce pharmaceutically relevant secondary metabolites. The next step would be to isolate members of these taxa in a targeted approach based on the media that best support their growth (Rettedal et al. 2014). The enriched cryostocks can be re-plated, and subsequently large numbers of colonies can be picked e.g. with the help of colony pickers (Lagier et al. 2012). One issue of analysing bacterial growth by scraping from agar media is that cell material from the inoculum can still be present, and as a result bacteria may be detected that did not actually grow. This is an important caveat of the plate scraping method that needs to be considered in growth experiments that use 16S rRNA gene amplicon sequencing. Previous studies that evaluated bacterial growth from sponge and human gut samples using this method did not examine this caveat (Hardoim et al. 2014; Rettedal et al. 2014). We acknowledged that this phenomenon could have occurred in our experiments when in one scraped community that was composed of only two visible colonies as many as 188 OTUs were detected. In addition, in some scraped communities composed of <10 colonies, numerous OTUs were detected that were also present in the sponge samples. However, these OTUs were detected at lower relative abundance in the cultivated fraction. For example, an OTU (DQ889875) that corresponded to a novel *δ-proteobacterium* was present with 12% relative abundance in the *P. ficiformis* sample, whereas it was present at a relative abundance of 2.7% in a *P. ficiformis*-scraped community that consisted of two colonies. We assume that this bacterium did not grow, even though we cannot exclude the possibility that microcolonies appeared that were invisible to the naked eye (Davis et al. 2011). In order to address this issue, we have applied conservative criteria before concluding bacterial growth. A separate issue is that glycerol-frozen sponge samples were used, and as such, bacteria that do not survive the freeze-thawing process are not recovered.

### Bacterial Diversity Captured by Picking of Individual Isolates

OTUs were assigned to picked colonies if they represented >50% of reads obtained for that colony. This assignment strategy was used in order to address the high number of OTUs that were detected by only one or a few reads and, in almost all cases, comprised the minority of reads assigned to a colony. In this way, we adopted a conservative approach and limited ourselves to strains from OTUs that are most likely truly represented by growing bacteria and discard potential sequence artefacts, even though OTUs detected by one or a few reads can still be informative about bacterial presence (Zhan et al. 2013). Our results showed that double-barcoded 16S rRNA gene sequencing can reliably identify a large number of bacterial colonies. Therefore, this method can be used to complement existing mass spectrometry-based methods (Lagier and Raoult 2016; Tani et al. 2012), with the advantage being that the phylogenetic signal of the isolates can be directly compared to other 16S rRNA gene data (e.g. data from bacterial communities or data in 16S rRNA gene databases).

Bacteria isolated from sponges typically belong to the *Actinobacteria, Bacteroidetes, Firmicutes* or *α*-, *β*- and *γ*-*Proteobacteria* (Hardoim et al. 2014; Muscholl-Silberhorn et al. 2008). Here, each of 47 isolates obtained from media supplemented with antibiotics belongs to these four phyla, and only in two cases the bacterium (or a similar strain from the same OTU) was also detected in the original sponge sample. This is consistent with previous studies that often found limited overlap between bacterial diversity detected by culture-dependent and culture-independent approaches (Donachie et al. 2007; Hardoim et al. 2014). OTU1749 was assigned to 223 colonies even though in the sponge samples this OTU was not detected, suggesting that *Pseudovibrio* spp. have a strong growth advantage on the applied cultivation media. *Pseudovibrio* spp. have been repeatedly isolated from marine sponges without being detected by cultivation-independent techniques (Schippers et al. 2012; Enticknap et al. 2006). These results imply that in sponges a vast number of different bacterial species reside that are not detected at read depths of at least 3﻿000–7﻿000 reads. Therefore, the presence of additional uncovered bacterial diversity is still to be expected.

To isolate a diverse range of bacteria, we also applied a polycarbonate filter that could mimic the inner structures of the filter-feeding sponge, an extended incubation period, poor and selective media and supplementation of antibiotics. We found that the application of a polycarbonate filter did not complement conventional plating with respect to retrieved bacterial diversity. Two bacterial strains were picked no earlier than 34 days post incubation. These were a *Mycobacterium peregrinum* (OTU FJ382132) and a bacterium that potentially belongs to a new genus in the *Flavobacteriaceae* family (OTU denovo1540). After transfer to fresh media, visible colonies grew after a week, which indicates that their initially slower growth might have been hampered due to adjustment to in vitro conditions. The *Flavobacteriaceae* sp. was originally isolated on mucin agar, but was also found to grow on marine agar. Two other strains, namely *B. pachyrhizi* (OTU FJ624884) and another novel *Flavobacteriaceae* sp. (OTU denovo1558), were isolated on 10-fold diluted marine agar and mucin agar, respectively, and both were unable to grow on (undiluted) marine agar. Lastly, on marine agar, a third novel *Flavobacteriaceae* sp. (OTU denovo1624) was isolated which potentially constitutes a new genus. These results demonstrate that longer incubation periods and a spectrum of different media were effective to capture a broader diversity of bacteria. *Pseudovibrio* spp. hindered the isolation of diverse bacteria by virtue of their fast (over-)growth. Bacteria from this genus often dominate cultured fractions of sponge-associated bacterial communities (Esteves et al. 2013; Muscholl-Silberhorn et al. 2008). *Pseudovibrio* spp. were sensitive to kanamycin, which aided in the isolation of four different bacterial strains from agar media supplemented with this antibiotic that were not retrieved from media supplemented with other antibiotics. In general, these results show that refinement of standard cultivation strategies, such as supplementation of media with antibiotics, can be used to acquire yet uncovered bacterial diversity. The success at acquisition of previously uncultivated bacteria is also related to sponges being relatively unexplored (Esteves et al. 2013; Sipkema et al. 2011; Alain and Querellou 2009).

### Sponge Bacteria as a Reservoir for Antibiotic Resistance Genes

Resistance genes were shown to be present (Durso et al. 2012) and expressed (Versluis et al. 2015) in all biological niches with complex microbial communities that so far have been investigated. It is therefore hypothesised that also sponge-associated bacteria harbour resistance genes that could (eventually) become available to human pathogens (Santos-Gandelman et al. 2013). The bacteria isolated in this study show resistance to a diverse range of antibiotics that are routinely used in the clinic. Publicly available genomes of a subset of these species indicated the potential presence of genes providing resistance to tetracyclines, β-lactams, aminoglycosides, chloramphenicol and erythromycin, frequently correlating with the observed resistance phenotypes of our corresponding strains. However, class A or B β-lactamase resistance genes were detected in all investigated public genomes even though resistance to ampicillin and/or penicillin was only observed in one out of six of our corresponding strains. Functional (meta-)genomic studies are suggested to identify mechanisms behind the observed antibiotic resistance, and to investigate if sponges are a reservoir for (novel) antibiotic resistance genes (Versluis et al. 2016).

## Conclusion

Using multiple cultivation conditions, we captured a breadth of previously uncultured sponge-associated bacteria, mainly from *A. aerophoba*. At the same time, we showed that double-barcoded 16S rRNA gene sequencing can be used to identify a large number of bacterial isolates, and we presented criteria to address an important caveat of the plate scraping method whereby bacteria may be detected that did not actually grow. We propose 27 OTUs that are of prime interest due to their novelty alone. Amongst these, a diverse range of novel *Actinobacteria* was cultivated, which shows that previously uncultivated bacteria with high biotechnological and pharmaceutical potential are still accessible by classic cultivation. One highly novel previously uncultured *Actinobacterium* was cultivated that was also detected in the original sponge sample. This strain appears to belong to a sponge-specific clade, which makes it particularly interesting in the study of host-symbiont relations. In addition, three *Flavobacteriaceae* spp. were cultured up to pure cultures and potentially constitute two new genera and one new species.

## Electronic supplementary material

ESM 1 (DOCX 28 kb)

ESM 2 (DOCX 813 kb)

ESM 3 (XLSX 18 kb)

ESM 4 (XLSX 13 kb)

ESM 5 (AI 27945 kb)

ESM 6 (DOCX 99 kb)

ESM 7 (XLSX 38 kb)

ESM 8 (XLSX 18 kb)
