# Supplementary material for: Recovery of Previously Uncultured Bacterial Genera from Three Mediterranean Sponges
Source: Mar Biotechnol (NY). 2017 Jul 10;19(5):454–68. doi: 10.1007/s10126-017-9766-4 (PMC5599449; doi:10.1007/s10126-017-9766-4)
Supplement: Supplementary file 1 — (DOCX 28 kb) [file 10126_2017_9766_MOESM1_ESM.docx]

**Supplementary Materials and Methods**

Marine Biotechnology

**Recovery of previously uncultured** **bacterial genera from three Mediterranean sponges**

**Dennis Versluis^1^, Kyle McPherson^1^, Mark W.J. van Passel^1,2^, Hauke Smidt^1^, Detmer Sipkema^1α^**

^1^Laboratory of Microbiology, Wageningen University & Research, Wageningen, The Netherlands

^2^National Institute for Public Health and the Environment, Bilthoven, The Netherlands

Corresponding author

α detmer.sipkema@wur.nl

**Barcoded 16S rRNA gene amplification and library preparation**

Barcoded 16S rRNA gene amplicon 454-pyrosequencing was done (I) to analyse bacterial communities present in the sponge samples, (II to identify bacterial colonies that were picked from media containing antibiotics, and (III) to analyse bacterial communities retrieved by scraping from media without antibiotics. PCR was performed to amplify an approximately 311 bp fragment comprising the V1 and V2 regions of the 16S rRNA gene. For this purpose, the DNA that was extracted from the samples was diluted to 10-20 ng/μl. In case of identification of bacterial colonies cell material in nuclease-free water served as template. The composite forward primer consisted of titanium sequencing adaptor A, a barcode (Hamady et al. 2008) and degenerate primer 27F-DegS (van den Bogert et al. 2011) (Supplementary Table S1). Reverse priming was done by an equimolar mixture of primers 338R-I and 338R-II (Daims et al. 1999), each attached to titanium sequencing adaptor B. For the identification of individually picked colonies, a barcode was also included in the reverse primer (for each colony a unique combination of barcodes was used). PCRs were performed in a total volume of 100 μl containing 20 μl 5X Phusion Green HF buffer (Thermo Fisher Scientific), 2 μl 10 mM dNTPs, 5 μl 10 μM forward primer, 5 μl 10 μM reverse primer, 1 μl Phusion Hot start II DNA polymerase (2 U/µl, Thermo Scientific) and 2 µl sample (10-20 ng/µl). The PCR program consisted of: initial denaturation of 30 s at 98 ^o^C; 30 cycles of denaturation at 98 ^o^C for 10 s, annealing at 56 ^o^C for 20 s, and extension at 72 ^o^C for 20 s; and final extension at 72 ^o^C for 10 min. At this point the identification of picked colonies required an additional experimental step wherein PCR products with identical barcodes in the forward primer (but different barcodes in the reverse primer) were pooled equimolarly (at maximum 48 different barcodes in the reverse primer were combined that had one distinct barcode in the forward primer). Either 5, 10 or 20 μl of PCR product was included in the pool, depending on agarose gel band intensity. Subsequently (pools of) PCR products were purified with the GeneJET PCR Purification Kit (Thermo Scientific) according to manufacturers’ instructions. Isopropanol was included in the binding buffer and elution buffer was re-applied to the column to increase the DNA yield. The DNA concentration of the elute was measured with the Qubit® 2.0 Fluorometer (Invitrogen) using the Qubit® dsDNA BR assay according to manufacturers’ instructions. An equimolar mixture of at maximum 54 purified PCR products containing different forward barcodes was prepared and run on a 1% agarose gel. The gel band at ~425 bp was cut from the gel and purified using the DNA Gel Extraction Kit (Millipore) according to manufacturers’ instructions. The DNA concentration of the mixture was measured by Qubit® 2.0 and sent for pyrosequencing at GATC Biotech (Konstanz, Germany) on a Roche GS FLX. Five libraries were sent for sequencing that contained samples particular to this study (Supplementary Table S1).

**Diversity and clustering analyses**

*Rarefactions curves*

For sponge bacterial communities, rarefaction curves were calculated with QIIME scripts (Caporaso et al. 2010) applying 10 iterations per subsampling of the OTU table. Subsamples were generated beginning with 100 reads, plus an additional 250 reads were added per subsampling step. QIIME was also used to calculate Shannon diversity metrics. Therefore, the number of reads in the OTU table per sample was rarefied using 1,000 iterations to match the sample with the smallest number of reads (i.e. Pf1 with 3,247 reads). The Shannon diversity metric was calculated for each of the 1,000 rarefied OTU tables and used to calculate an average value per sample.

*Hierarchical clustering*

Hierarchical clustering was performed using R package ‘Vegan’ (Dixon 2003) based on OTU-level relative abundance data obtained from bacterial communities in sponge tissues as well as those scraped from agar plates. Firstly, a matrix of Bray-Curtis dissimilarity values was created with Vegan function ‘vegdist’. Thereafter, the dissimilarity matrix was used for average hierarchical clustering with hclust, and finally, a dendrogram was created using the function ‘as.dendrogram’.

*Canonical (constrained) correspondence analysis*

Canonical (constrained) correspondence analysis as implemented in Canoco 5 (Šmilauer and Lepš 2014) was used to investigate which experimental variables best explain the variation in species composition regarding square-rooted OTU-level relative abundance data obtained from the bacterial communities scraped from agar plates. Interactive-forward-selection analysis was selected with ‘sponge species’, ‘day of harvesting’, ‘medium’ and ‘growth surface’ (agar or filter) as explanatory variables. Explanatory variables at the forward selection step were included in order of contribution to the explained variation until the value of the adjusted R^2^ including the current best candidate exceeded the threshold based on a model with all predictors.

*SIMPER*

SIMPER analysis (Clarke and Gorley 2006) was used with respect to square rooted OTU-level relative abundance data to break down the contribution of each OTU to the observed dissimilarity between sample groups. Pairwise comparisons were performed between samples from scraped communities that were grouped based on sponge species, day of harvesting, medium and growth surface.

*Phylogenetic tree construction*

The tree was constructed in ARB (Ludwig et al. 2004) by Maximum likelihood analysis using 1,000 iterations of RAxML rapid bootstrapping. For tree calculation, highly variable positions (1-9) were excluded using the bacterial Positional Variability by Parsimony filter, and non-overlapping regions were excluded (window of inclusion, position 5,331 to 26,803). After tree construction, representative pyrosequencing reads belonging to OTUs for which unsuccessful attempts were made to obtain a member in pure culture were added using “add species to existing tree” with ARB_Parsimony, applying similar filtering settings as those used for the base tree.

**References**

Caporaso JG, Kuczynski J, Stombaugh J, Bittinger K, Bushman FD, Costello EK, Fierer N, Pena AG, Goodrich JK, Gordon JI, Huttley GA, Kelley ST, Knights D, Koenig JE, Ley RE, Lozupone CA, Mcdonald D, Muegge BD, Pirrung M, Reeder J, Sevinsky JR, Turnbaugh PJ, Walters WA, Widmann J, Yatsunenko T, Zaneveld J, Knight, R (2010) QIIME allows analysis of high-throughput community sequencing data. Nat Methods 7**:** 335-6

Clarke K, Gorley R (2006) PRIMER v6: User Manual/Tutorial, PRIMER-E

Daims H, Bruhl A, Amann R, Schleifer KH, Wagner M (1999) The domain-specific probe EUB338 is insufficient for the detection of all Bacteria: Development and evaluation of a more comprehensive probe set. Syst Appl Microbiol 22**:** 434-444

Dixon, P. (2003) VEGAN, a package of R functions for community ecology. J Veg. Sci 14: 927-930

Hamady M, Walker JJ, Harris JK, Gold NJ, Knight R (2008) Error-correcting barcoded primers for pyrosequencing hundreds of samples in multiplex. Nat Methods 5**:** 235-7

Ludwig W, Strunk O, Westram R, Richter L, Meier H, Yadhukumar, Buchner A, Lai T, Steppi S, Jobb G, Forster W, Brettske I, Gerber S, Ginhart AW, Gross O, Grumann S, Hermann S, Jost R, Konig A, Liss T, Lussmann R, May M, Nonhoff B, Reichel B, Strehlow R, Stamatakis A, Stuckmann N, Vilbig A, Lenke M, Ludwig T, Bode A, Schleifer KH (2004) ARB: a software environment for sequence data. Nucleic Acids Res 32**:** 1363-71

Šmilauer P, Lepš, J (2014) Multivariate analysis of ecological data using Canoco 5. Cambridge University Press, New York

Van Den Bogert B, De Vos WM, Zoetendal EG, Kleerebezem M (2011) Microarray analysis and barcoded pyrosequencing provide consistent microbial profiles depending on the source of human intestinal samples. Appl Environ Microbiol 77**:** 2071-80
