# Supplementary material for: Recovery of Previously Uncultured Bacterial Genera from Three Mediterranean Sponges
Source: Mar Biotechnol (NY). 2017 Jul 10;19(5):454–68. doi: 10.1007/s10126-017-9766-4 (PMC5599449; doi:10.1007/s10126-017-9766-4)
Supplement: Supplementary file 2 — (DOCX 813 kb) [file 10126_2017_9766_MOESM2_ESM.docx]

Supplementary Figures S1-S5

## Marine Biotechnology

**Recovery of previously uncultured bacterial genera from three Mediterranean sponges**

**Dennis Versluis1, Kyle McPherson1, Mark W.J. van Passel1,2, Hauke Smidt1, Detmer Sipkema1 α**

### 1Laboratory of Microbiology, Wageningen University & Research, Wageningen, The Netherlands

2National Institute for Public Health and the Environment, Bilthoven, The Netherlands

Corresponding author

α [detmer.sipkema@wur.nl](mailto:detmer.sipkema@wur.nl)


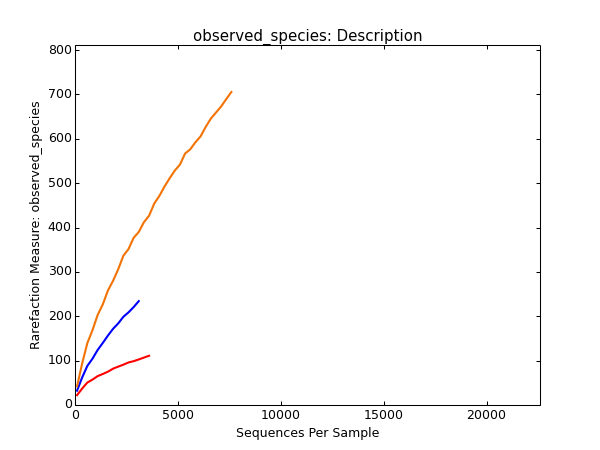


**Supplementary Figure S1** Rarefaction curves in which is plotted the number of OTUs detected in samples from the sponges *A. aerophoba* (yellow)*, P. ficiformis* (blue) and *C. candelabrum* (red) as a function of the number of reads sampled.

**Supplementary Figure S2** Canonical correspondence analysis was used to investigate which environmental variables could explain the variation in square-rooted OTU-level relative abundance data of bacterial communities scraped from agar plates.

### % identity to closest type strain

### 10log relative abundance

0 0,930546624

0 0,103236527

0 0,224059293

0 0,135336594

0 0,030760986

0 0,06299886

0 0,012518741

0 0,037343216

0 0,073511543

0 0,007152146

0,027339004

0 0,021664766

0,032806804

0 0,004912585

0 0,017103763

0,030376671

0 0,003395463

0 0,02490887

0 0,010832383

0 0,002005964

0 0,002908469

0,0329535 0,010935601


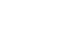

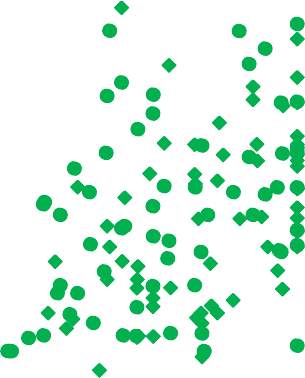


75

80

85

90

95

100

1

0,1

0,01

0,001

0,0001

**Supplementary Figure S3** The relative abundance values of OTUs that were detected with >4 reads in the communities scraped from agar media are plotted against the identity of the corresponding read to the closest type strain, as determined

by Blastn. The highest relative abundance value is shown if the OTU was detected in multiple scraped communities. The OTUs are shown separately for each of the three sponges: *A. aerophoba* (circles), *P. ficiformis* (diamonds) and *C. candelabrum* (triangles). Data points are marked green if the relative abundance of the OTU in the scraped fraction is higher than in the

in the sponge sample (the inoculum) or in red if the converse is true.

OTU

U81990 (24)

JN874385 (2)

JN579972 (1)

JN038312 (4)

JF937433 (2)

JF937422 (4)

HQ671075 (6)

HQ326290 (2)

HM595366 (7)

HE574879 (3)

GU940713 (1)

GU225820 (1)

GQ118701 (4)

FJ808721 (8)

FJ751910 (1)

FJ624884 (26)

FJ382132 (1)

FJ203135 (3)

FJ191674 (1)

FJ169195 (3)

EF123323 (2)

DQ860060 (1)

DQ446109 (1)

denovo828 (1)

denovo605 (4)

denovo552 (7)

denovo520 (1)

denovo473 (4)

denovo2037 (1)

denovo198 (4)

denovo1888 (10)

denovo1754 (1)

denovo1749 (223)

denovo1627 (1)

denovo1624 (1)

denovo1600 (2)

denovo1558 (13)

denovo1540 (2)

denovo1345 (2)

denovo1338 (1)

denovo1231 (1)

denovo1026 (27)

AY959053 (4)

AM709702 (3)

AJ875023 (1)

AJ633970 (2)

AB572583 (1)

0 10 20 30 40 50 60 70 80

### Average day of isolate picking

**Supplementary Figure S4** An OTU was assigned to a colony if represented by >50 % of reads pertaining to that isolate. For each of these 47 OTUs is shown on average the day at which the corresponding isolates were picked. The value between brackets behind the OTU name indicates the number of strains that were picked belonging to this OTU. Error bars representing standard deviations are given if >1 isolate was obtained. For OTUs with green bars an isolate was obtained in pure culture whereas for those with red bars regrowth was unsuccessful.

## 600

500

400

**Frequency**

300

200

100

0

**Relative abundance**

**Supplementary Figure S5** For each OTU assigned to a picked colony, its relative abundance was calculated by dividing the number of reads with which this OTU was detected by the total number of reads assigned to the colony. By this manner a list of relative abundance values was generated, and the distribution of these values is shown here. Note that a high number OTUs were assigned to colonies with <10% relative abundance (in most cases these OTUs are detected by one read only), which are expected to be mostly sequencing artefacts.
