# Supplementary material for: Recovery of Previously Uncultured Bacterial Genera from Three Mediterranean Sponges
Source: Mar Biotechnol (NY). 2017 Jul 10;19(5):454–68. doi: 10.1007/s10126-017-9766-4 (PMC5599449; doi:10.1007/s10126-017-9766-4)
Supplement: Supplementary file 6 — (DOCX 99 kb) [file 10126_2017_9766_MOESM6_ESM.docx]

**"Recovery of previously uncultured bacterial genera from three Mediterranean sponges"**

by Dennis Versluis, Kyle McPherson, Mark W.J. van Passel, Hauke Smidt and Detmer Sipkema

in *Marine Biotechnology*

**Supplementary Table S3** SIMPER was used to analyse square rooted OTU-level relative abundance data that was obtained from bacterial communities scraped from agar plates. The analysis aimed to break down the contribution of each OTU to the observed dissimilarity between samples grouped by day of harvesting **(A)**, growth medium **(B)**, growth on agar vs. growth on a filter on top of agar **(C)**, and sponge species **(D)**. The raw PRIMER 6 output for the comparisons of all combinations of sample groups are shown in this sheet. The first column lists the OTUs. The OTUs are ranked from those that contribute most to those that contribute least to the dissimilarity of sample groups. The second and third columns list the average relative abundance of the OTUs in the sample groups that were compared. The final column provides the cumulative percentage that OTUs contribute to the dissimilarity between sample groups.

**(A)**

Groups 15 & 30

Average dissimilarity = 63.59

Group 15 Group 30

Species Av.Abund Av.Abund Av.Diss Diss/SD Contrib% Cum.%

denovo1749 0.84 0.68 6.96 0.82 10.95 10.95

GQ118701 0.10 0.06 2.49 0.83 3.92 14.87

denovo1026 0.13 0.08 2.11 0.81 3.31 18.18

JN579972 0.00 0.09 1.88 0.33 2.95 21.13

denovo75 0.06 0.02 1.45 0.31 2.28 23.41

HE574879 0.05 0.03 1.41 0.67 2.21 25.62

HM595366 0.03 0.04 1.10 0.41 1.73 27.35

FJ516885 0.00 0.03 1.05 0.20 1.65 28.99

denovo1888 0.02 0.04 1.00 0.42 1.57 30.56

JF937422 0.12 0.02 0.98 0.79 1.54 32.10

denovo1484 0.10 0.02 0.87 0.82 1.36 33.46

FJ624884 0.02 0.04 0.84 0.35 1.32 34.78

JN030555 0.00 0.03 0.77 0.19 1.20 35.98

U81990 0.02 0.02 0.69 0.42 1.09 37.07

denovo427 0.02 0.02 0.67 0.86 1.05 38.12

denovo552 0.02 0.01 0.64 0.65 1.01 39.13

denovo1146 0.02 0.02 0.63 0.92 0.99 40.11

denovo189 0.02 0.02 0.60 0.85 0.95 41.06

denovo547 0.02 0.01 0.55 0.79 0.87 41.93

denovo1017 0.10 0.01 0.53 0.40 0.83 42.76

JN874385 0.01 0.01 0.50 0.62 0.79 43.54

AM709702 0.01 0.02 0.50 0.36 0.78 44.32

FJ751910 0.00 0.02 0.48 0.18 0.75 45.08

JF802167 0.00 0.02 0.44 0.20 0.69 45.77

denovo109 0.02 0.01 0.43 0.81 0.68 46.44

denovo1249 0.02 0.01 0.43 1.00 0.67 47.12

denovo605 0.01 0.02 0.42 0.36 0.67 47.78

denovo507 0.01 0.01 0.42 0.83 0.67 48.45

denovo1189 0.01 0.01 0.41 0.84 0.64 49.09

AY914065 0.02 0.01 0.38 0.72 0.60 49.69

AY739689 0.00 0.02 0.36 0.18 0.57 50.26

denovo119 0.01 0.01 0.35 0.70 0.55 50.81

HQ245988 0.00 0.02 0.35 0.20 0.55 51.36

denovo838 0.07 0.01 0.32 0.35 0.50 51.86

denovo1479 0.01 0.01 0.32 0.55 0.50 52.36

denovo862 0.01 0.01 0.31 0.34 0.48 52.84

EU935300 0.02 0.01 0.30 0.71 0.48 53.31

EU846601 0.00 0.02 0.30 0.18 0.47 53.79

denovo114 0.08 0.00 0.30 0.31 0.47 54.25

denovo756 0.07 0.00 0.28 0.31 0.44 54.69

AB176201 0.01 0.01 0.28 0.59 0.44 55.13

AB299573 0.02 0.00 0.28 0.19 0.43 55.57

GU940713 0.02 0.00 0.28 0.54 0.43 56.00

AF286480 0.00 0.01 0.27 0.50 0.43 56.43

denovo953 0.01 0.01 0.27 0.63 0.42 56.85

denovo1873 0.01 0.00 0.26 0.33 0.41 57.26

denovo1022 0.01 0.00 0.26 0.46 0.41 57.68

denovo1905 0.10 0.00 0.26 0.20 0.40 58.08

denovo204 0.01 0.01 0.24 0.73 0.38 58.46

denovo1070 0.01 0.01 0.24 0.80 0.38 58.84

denovo1558 0.01 0.00 0.24 0.34 0.38 59.22

denovo1273 0.01 0.00 0.24 0.63 0.37 59.59

denovo1743 0.07 0.00 0.23 0.25 0.36 59.96

denovo1961 0.01 0.01 0.23 0.37 0.36 60.32

EU035954 0.01 0.01 0.22 0.42 0.35 60.66

denovo545 0.01 0.01 0.22 1.23 0.35 61.01

denovo141 0.01 0.01 0.22 0.74 0.35 61.36

denovo1476 0.07 0.00 0.21 0.24 0.34 61.69

denovo828 0.01 0.00 0.21 0.35 0.34 62.03

denovo324 0.07 0.00 0.21 0.23 0.32 62.35

denovo1791 0.07 0.00 0.21 0.23 0.32 62.68

denovo1243 0.00 0.01 0.20 0.33 0.31 62.99

DQ889875 0.01 0.01 0.19 0.37 0.30 63.29

denovo415 0.01 0.01 0.19 0.98 0.29 63.58

EU536078 0.01 0.00 0.19 0.52 0.29 63.88

denovo1322 0.07 0.00 0.18 0.20 0.28 64.16

denovo854 0.00 0.00 0.18 0.46 0.28 64.44

AY631057 0.00 0.01 0.18 0.21 0.28 64.72

denovo1842 0.01 0.00 0.17 0.73 0.27 64.99

HQ118426 0.00 0.01 0.17 0.35 0.27 65.26

denovo1085 0.00 0.00 0.17 0.71 0.27 65.53

denovo1565 0.07 0.00 0.17 0.19 0.27 65.80

denovo1640 0.07 0.00 0.17 0.19 0.27 66.06

denovo1785 0.07 0.00 0.17 0.19 0.27 66.33

GQ385289 0.07 0.00 0.17 0.19 0.27 66.60

AM992178 0.00 0.01 0.17 0.19 0.26 66.86

denovo1720 0.00 0.01 0.17 0.41 0.26 67.12

denovo1145 0.01 0.00 0.17 0.84 0.26 67.38

HQ721418 0.01 0.00 0.17 0.62 0.26 67.65

denovo1462 0.00 0.00 0.16 0.65 0.26 67.90

AM935808 0.01 0.00 0.16 0.18 0.25 68.15

denovo792 0.00 0.01 0.15 0.31 0.24 68.39

HQ616267 0.01 0.00 0.15 0.74 0.24 68.63

AJ292596 0.00 0.01 0.15 0.32 0.24 68.87

X97093 0.01 0.00 0.15 0.59 0.24 69.11

denovo1292 0.00 0.00 0.14 0.49 0.23 69.34

DQ860060 0.01 0.00 0.14 0.62 0.22 69.56

GU118526 0.01 0.01 0.14 0.28 0.22 69.77

AF170746 0.01 0.00 0.14 0.39 0.22 69.99

denovo430 0.00 0.00 0.14 0.76 0.21 70.20

HM108471 0.01 0.00 0.13 0.57 0.21 70.41

GU179554 0.01 0.00 0.13 0.54 0.20 70.62

FQ659744 0.00 0.01 0.13 0.18 0.20 70.82

denovo1848 0.00 0.01 0.12 0.18 0.19 71.01

denovo709 0.00 0.00 0.12 0.65 0.19 71.20

denovo1547 0.01 0.00 0.12 0.34 0.19 71.38

AB477015 0.01 0.00 0.12 0.55 0.18 71.57

HQ323455 0.01 0.00 0.12 0.20 0.18 71.75

denovo1632 0.00 0.00 0.12 0.65 0.18 71.93

denovo1753 0.00 0.00 0.11 0.27 0.18 72.11

denovo1162 0.01 0.00 0.11 0.34 0.17 72.29

denovo1703 0.00 0.00 0.11 0.35 0.17 72.46

denovo198 0.00 0.00 0.11 0.28 0.17 72.63

denovo1374 0.00 0.00 0.11 0.31 0.17 72.80

denovo1923 0.00 0.00 0.11 0.40 0.17 72.97

JF802166 0.00 0.01 0.10 0.18 0.16 73.13

JF937433 0.00 0.00 0.10 0.41 0.16 73.29

denovo1577 0.00 0.00 0.10 0.42 0.16 73.45

AF252321 0.00 0.01 0.10 0.22 0.16 73.61

denovo293 0.00 0.00 0.10 0.24 0.15 73.76

denovo1412 0.00 0.01 0.10 0.29 0.15 73.92

denovo2019 0.00 0.00 0.10 0.35 0.15 74.07

denovo1306 0.00 0.00 0.10 0.25 0.15 74.22

denovo1420 0.00 0.00 0.09 0.62 0.15 74.36

denovo1129 0.00 0.00 0.09 0.32 0.15 74.51

JN868782 0.00 0.00 0.09 0.52 0.15 74.66

EU160577 0.01 0.00 0.09 0.45 0.14 74.80

FJ957855 0.00 0.00 0.09 0.18 0.14 74.94

denovo1579 0.00 0.00 0.09 0.65 0.14 75.09

HE574926 0.00 0.00 0.09 0.44 0.14 75.22

denovo1491 0.00 0.00 0.09 0.21 0.14 75.36

denovo550 0.00 0.01 0.09 0.18 0.13 75.49

AJ633944 0.00 0.00 0.08 0.35 0.13 75.62

AJ347026 0.00 0.00 0.08 0.31 0.13 75.75

FJ418924 0.01 0.00 0.08 0.31 0.12 75.87

denovo1190 0.00 0.00 0.08 0.34 0.12 76.00

FJ957708 0.00 0.00 0.08 0.18 0.12 76.12

denovo927 0.00 0.00 0.08 0.25 0.12 76.24

denovo916 0.00 0.00 0.08 0.26 0.12 76.36

denovo607 0.00 0.00 0.08 0.34 0.12 76.49

denovo776 0.00 0.00 0.08 0.33 0.12 76.61

denovo1182 0.00 0.00 0.08 0.34 0.12 76.73

denovo1263 0.00 0.00 0.08 0.18 0.12 76.85

FJ849072 0.00 0.00 0.08 0.18 0.12 76.96

denovo767 0.00 0.00 0.07 0.31 0.12 77.08

denovo40 0.00 0.00 0.07 0.23 0.12 77.20

denovo2003 0.00 0.00 0.07 0.35 0.11 77.31

denovo95 0.00 0.00 0.07 0.22 0.11 77.42

denovo1996 0.00 0.00 0.07 0.18 0.11 77.53

FJ169195 0.00 0.00 0.07 0.42 0.11 77.64

denovo822 0.00 0.00 0.07 0.29 0.11 77.75

denovo190 0.00 0.00 0.07 0.51 0.11 77.86

denovo1096 0.00 0.00 0.07 0.25 0.11 77.96

denovo790 0.00 0.00 0.07 0.53 0.11 78.07

denovo1390 0.00 0.00 0.07 0.47 0.11 78.18

denovo61 0.00 0.00 0.07 0.43 0.11 78.28

denovo6 0.00 0.00 0.07 0.27 0.11 78.39

denovo1341 0.00 0.00 0.07 0.30 0.11 78.49

GQ903461 0.00 0.00 0.06 0.28 0.10 78.60

GU118575 0.00 0.00 0.06 0.35 0.10 78.70

denovo1193 0.00 0.00 0.06 0.18 0.10 78.79

EU803928 0.01 0.00 0.06 0.35 0.10 78.89

HQ671075 0.00 0.00 0.06 0.20 0.10 78.99

FJ892785 0.00 0.00 0.06 0.43 0.09 79.08

denovo1789 0.00 0.00 0.06 0.33 0.09 79.17

GU305772 0.00 0.00 0.06 0.50 0.09 79.26

denovo1250 0.00 0.00 0.06 0.39 0.09 79.35

GU553034 0.00 0.00 0.06 0.21 0.09 79.43

FJ826409 0.00 0.00 0.06 0.34 0.09 79.52

HQ326290 0.00 0.00 0.05 0.35 0.09 79.61

denovo1921 0.00 0.00 0.05 0.20 0.08 79.69

FJ203135 0.00 0.00 0.05 0.25 0.08 79.78

denovo43 0.00 0.00 0.05 0.53 0.08 79.86

denovo1478 0.00 0.00 0.05 0.18 0.08 79.95

denovo1570 0.00 0.00 0.05 0.18 0.08 80.03

denovo2056 0.00 0.00 0.05 0.18 0.08 80.11

denovo757 0.00 0.00 0.05 0.18 0.08 80.20

JQ032050 0.00 0.00 0.05 0.18 0.08 80.28

denovo1480 0.00 0.00 0.05 0.30 0.08 80.36

denovo1446 0.00 0.00 0.05 0.25 0.08 80.45

denovo499 0.00 0.00 0.05 0.26 0.08 80.53

denovo1898 0.00 0.00 0.05 0.43 0.08 80.61

denovo1964 0.00 0.00 0.05 0.31 0.08 80.68

denovo2029 0.00 0.00 0.05 0.24 0.08 80.76

denovo1284 0.00 0.00 0.05 0.32 0.08 80.84

GQ263306 0.00 0.00 0.05 0.38 0.08 80.92

GQ433928 0.00 0.00 0.05 0.24 0.08 81.00

denovo249 0.00 0.00 0.05 0.46 0.08 81.07

GQ921403 0.00 0.00 0.05 0.18 0.08 81.15

denovo98 0.00 0.00 0.05 0.31 0.08 81.23

GU118606 0.00 0.00 0.05 0.37 0.08 81.30

DQ316817 0.00 0.00 0.05 0.18 0.08 81.38

GU118690 0.00 0.00 0.05 0.32 0.07 81.45

denovo294 0.00 0.00 0.05 0.28 0.07 81.53

denovo1592 0.00 0.00 0.05 0.18 0.07 81.60

denovo325 0.00 0.00 0.05 0.18 0.07 81.67

denovo1757 0.00 0.00 0.05 0.29 0.07 81.75

denovo696 0.00 0.00 0.05 0.18 0.07 81.82

denovo663 0.00 0.00 0.05 0.31 0.07 81.89

denovo777 0.00 0.00 0.05 0.40 0.07 81.96

denovo574 0.00 0.00 0.04 0.27 0.07 82.03

denovo815 0.00 0.00 0.04 0.40 0.07 82.10

denovo1619 0.00 0.00 0.04 0.34 0.07 82.17

denovo7 0.00 0.00 0.04 0.46 0.07 82.23

denovo676 0.00 0.00 0.04 0.42 0.07 82.30

EF076171 0.00 0.00 0.04 0.40 0.07 82.37

AB294294 0.00 0.00 0.04 0.22 0.07 82.43

denovo1832 0.00 0.00 0.04 0.45 0.07 82.50

DQ889881 0.00 0.00 0.04 0.32 0.07 82.57

JF925031 0.00 0.00 0.04 0.40 0.06 82.63

denovo1379 0.00 0.00 0.04 0.35 0.06 82.69

denovo729 0.00 0.00 0.04 0.36 0.06 82.76

EU463921 0.00 0.00 0.04 0.26 0.06 82.82

denovo657 0.00 0.00 0.04 0.25 0.06 82.89

denovo706 0.00 0.00 0.04 0.21 0.06 82.95

EF613488 0.00 0.00 0.04 0.25 0.06 83.01

denovo320 0.00 0.00 0.04 0.45 0.06 83.07

FJ900573 0.00 0.00 0.04 0.32 0.06 83.14

denovo1436 0.00 0.00 0.04 0.27 0.06 83.20

AY959053 0.00 0.00 0.04 0.38 0.06 83.26

denovo1230 0.00 0.00 0.04 0.40 0.06 83.32

denovo917 0.00 0.00 0.04 0.21 0.06 83.38

FJ557790 0.00 0.00 0.04 0.29 0.06 83.44

denovo520 0.00 0.00 0.04 0.28 0.06 83.49

AJ240982 0.00 0.00 0.04 0.22 0.06 83.55

denovo1880 0.00 0.00 0.04 0.18 0.06 83.61

denovo457 0.00 0.00 0.04 0.29 0.06 83.67

FJ820465 0.00 0.00 0.04 0.26 0.06 83.72

denovo412 0.00 0.00 0.04 0.28 0.06 83.78

denovo172 0.00 0.00 0.04 0.35 0.06 83.83

denovo423 0.00 0.00 0.03 0.26 0.05 83.89

FJ802385 0.00 0.00 0.03 0.32 0.05 83.94

denovo788 0.00 0.00 0.03 0.18 0.05 84.00

denovo853 0.00 0.00 0.03 0.34 0.05 84.05

denovo639 0.00 0.00 0.03 0.18 0.05 84.11

FN396956 0.00 0.00 0.03 0.18 0.05 84.16

denovo528 0.00 0.00 0.03 0.20 0.05 84.21

denovo679 0.00 0.00 0.03 0.22 0.05 84.26

denovo305 0.00 0.00 0.03 0.31 0.05 84.32

FJ457296 0.00 0.00 0.03 0.22 0.05 84.37

denovo1423 0.00 0.00 0.03 0.37 0.05 84.42

AJ621576 0.00 0.00 0.03 0.38 0.05 84.47

denovo659 0.00 0.00 0.03 0.37 0.05 84.52

denovo87 0.00 0.00 0.03 0.30 0.05 84.57

denovo744 0.00 0.00 0.03 0.30 0.05 84.62

denovo1696 0.00 0.00 0.03 0.22 0.05 84.68

denovo1282 0.00 0.00 0.03 0.35 0.05 84.73

denovo1694 0.00 0.00 0.03 0.35 0.05 84.78

denovo1500 0.00 0.00 0.03 0.18 0.05 84.82

denovo1556 0.00 0.00 0.03 0.25 0.05 84.87

FJ381979 0.00 0.00 0.03 0.32 0.05 84.92

FJ497578 0.00 0.00 0.03 0.30 0.05 84.97

HE574895 0.00 0.00 0.03 0.18 0.05 85.02

FJ999591 0.00 0.00 0.03 0.29 0.05 85.06

JF712668 0.00 0.00 0.03 0.35 0.05 85.11

denovo193 0.00 0.00 0.03 0.31 0.05 85.16

denovo1983 0.00 0.00 0.03 0.31 0.05 85.20

HQ753432 0.00 0.00 0.03 0.31 0.05 85.25

EF018153 0.00 0.00 0.03 0.18 0.05 85.29

denovo2018 0.00 0.00 0.03 0.25 0.04 85.34

denovo1590 0.00 0.00 0.03 0.31 0.04 85.38

denovo1321 0.00 0.00 0.03 0.26 0.04 85.43

denovo1459 0.00 0.00 0.03 0.37 0.04 85.47

denovo1244 0.00 0.00 0.03 0.18 0.04 85.51

denovo719 0.00 0.00 0.03 0.29 0.04 85.56

denovo1701 0.00 0.00 0.03 0.36 0.04 85.60

denovo1088 0.00 0.00 0.03 0.31 0.04 85.64

denovo88 0.00 0.00 0.03 0.18 0.04 85.68

denovo1199 0.00 0.00 0.03 0.18 0.04 85.73

denovo1298 0.00 0.00 0.03 0.18 0.04 85.77

denovo1830 0.00 0.00 0.03 0.18 0.04 85.81

denovo1892 0.00 0.00 0.03 0.18 0.04 85.85

denovo481 0.00 0.00 0.03 0.18 0.04 85.89

denovo694 0.00 0.00 0.03 0.18 0.04 85.94

denovo946 0.00 0.00 0.03 0.18 0.04 85.98

DQ833490 0.00 0.00 0.03 0.18 0.04 86.02

HM243844 0.00 0.00 0.03 0.18 0.04 86.06

denovo1889 0.00 0.00 0.03 0.36 0.04 86.11

denovo1414 0.00 0.00 0.03 0.18 0.04 86.15

denovo1801 0.00 0.00 0.03 0.36 0.04 86.19

JN177591 0.00 0.00 0.03 0.24 0.04 86.23

denovo613 0.00 0.00 0.03 0.36 0.04 86.27

DQ396132 0.00 0.00 0.03 0.25 0.04 86.31

denovo165 0.00 0.00 0.03 0.23 0.04 86.35

GU584610 0.00 0.00 0.03 0.24 0.04 86.39

denovo1526 0.00 0.00 0.03 0.18 0.04 86.43

denovo1014 0.00 0.00 0.03 0.26 0.04 86.47

denovo993 0.00 0.00 0.03 0.26 0.04 86.51

denovo1581 0.00 0.00 0.03 0.32 0.04 86.55

denovo1062 0.00 0.00 0.03 0.36 0.04 86.59

denovo1621 0.00 0.00 0.03 0.30 0.04 86.63

denovo1705 0.00 0.00 0.03 0.18 0.04 86.67

denovo1962 0.00 0.00 0.03 0.37 0.04 86.71

denovo308 0.00 0.00 0.03 0.32 0.04 86.75

denovo611 0.00 0.00 0.02 0.18 0.04 86.79

denovo312 0.00 0.00 0.02 0.31 0.04 86.83

denovo1637 0.00 0.00 0.02 0.31 0.04 86.87

denovo143 0.00 0.00 0.02 0.23 0.04 86.91

denovo103 0.00 0.00 0.02 0.18 0.04 86.95

denovo28 0.00 0.00 0.02 0.25 0.04 86.99

EF076172 0.00 0.00 0.02 0.31 0.04 87.02

denovo1585 0.00 0.00 0.02 0.25 0.04 87.06

denovo1093 0.00 0.00 0.02 0.18 0.04 87.10

denovo1131 0.00 0.00 0.02 0.18 0.04 87.14

FJ529262 0.00 0.00 0.02 0.30 0.04 87.17

denovo1132 0.00 0.00 0.02 0.18 0.04 87.21

denovo860 0.00 0.00 0.02 0.18 0.04 87.25

denovo812 0.00 0.00 0.02 0.29 0.04 87.29

denovo707 0.00 0.00 0.02 0.26 0.04 87.32

EF153415 0.00 0.00 0.02 0.31 0.04 87.36

denovo473 0.00 0.00 0.02 0.18 0.04 87.40

HQ672216 0.00 0.00 0.02 0.25 0.04 87.43

denovo1670 0.00 0.00 0.02 0.25 0.04 87.47

GU584786 0.00 0.00 0.02 0.31 0.04 87.50

denovo1688 0.00 0.00 0.02 0.18 0.04 87.54

AY922243 0.00 0.00 0.02 0.25 0.04 87.58

denovo1303 0.00 0.00 0.02 0.24 0.04 87.61

HQ616309 0.00 0.00 0.02 0.26 0.04 87.65

denovo1899 0.00 0.00 0.02 0.25 0.03 87.68

HQ118340 0.00 0.00 0.02 0.30 0.03 87.72

AJ583166 0.00 0.00 0.02 0.22 0.03 87.75

denovo1555 0.00 0.00 0.02 0.24 0.03 87.79

EU817491 0.00 0.00 0.02 0.25 0.03 87.82

denovo1291 0.00 0.00 0.02 0.23 0.03 87.85

denovo1338 0.00 0.00 0.02 0.18 0.03 87.89

FQ660217 0.00 0.00 0.02 0.26 0.03 87.92

AJ867671 0.00 0.00 0.02 0.31 0.03 87.96

HQ588359 0.00 0.00 0.02 0.18 0.03 87.99

denovo1944 0.00 0.00 0.02 0.31 0.03 88.03

denovo521 0.00 0.00 0.02 0.18 0.03 88.06

denovo690 0.00 0.00 0.02 0.26 0.03 88.09

denovo692 0.00 0.00 0.02 0.31 0.03 88.13

GQ274045 0.00 0.00 0.02 0.25 0.03 88.16

denovo1118 0.00 0.00 0.02 0.24 0.03 88.19

denovo1641 0.00 0.00 0.02 0.18 0.03 88.22

JF808978 0.00 0.00 0.02 0.31 0.03 88.26

denovo1561 0.00 0.00 0.02 0.24 0.03 88.29

denovo492 0.00 0.00 0.02 0.31 0.03 88.32

denovo990 0.00 0.00 0.02 0.18 0.03 88.36

denovo1517 0.00 0.00 0.02 0.25 0.03 88.39

AB176169 0.00 0.00 0.02 0.22 0.03 88.42

FJ957596 0.00 0.00 0.02 0.30 0.03 88.45

denovo1403 0.00 0.00 0.02 0.31 0.03 88.48

JN411267 0.00 0.00 0.02 0.25 0.03 88.52

DQ889935 0.00 0.00 0.02 0.26 0.03 88.55

denovo24 0.00 0.00 0.02 0.26 0.03 88.58

denovo272 0.00 0.00 0.02 0.25 0.03 88.61

FJ808721 0.00 0.00 0.02 0.31 0.03 88.64

denovo1690 0.00 0.00 0.02 0.25 0.03 88.67

denovo1401 0.00 0.00 0.02 0.31 0.03 88.70

denovo1827 0.00 0.00 0.02 0.32 0.03 88.73

denovo1147 0.00 0.00 0.02 0.22 0.03 88.77

denovo850 0.00 0.00 0.02 0.31 0.03 88.80

denovo887 0.00 0.00 0.02 0.26 0.03 88.83

denovo1082 0.00 0.00 0.02 0.18 0.03 88.86

denovo1452 0.00 0.00 0.02 0.31 0.03 88.89

denovo1823 0.00 0.00 0.02 0.31 0.03 88.92

denovo184 0.00 0.00 0.02 0.31 0.03 88.95

denovo148 0.00 0.00 0.02 0.18 0.03 88.98

denovo1265 0.00 0.00 0.02 0.26 0.03 89.01

denovo1346 0.00 0.00 0.02 0.31 0.03 89.04

denovo1754 0.00 0.00 0.02 0.25 0.03 89.07

denovo1875 0.00 0.00 0.02 0.18 0.03 89.10

denovo771 0.00 0.00 0.02 0.25 0.03 89.13

denovo1917 0.00 0.00 0.02 0.26 0.03 89.15

denovo210 0.00 0.00 0.02 0.25 0.03 89.18

AJ863291 0.00 0.00 0.02 0.18 0.03 89.21

denovo1274 0.00 0.00 0.02 0.18 0.03 89.24

denovo60 0.00 0.00 0.02 0.25 0.03 89.27

denovo34 0.00 0.00 0.02 0.18 0.03 89.30

denovo1441 0.00 0.00 0.02 0.32 0.03 89.33

denovo608 0.00 0.00 0.02 0.25 0.03 89.36

denovo183 0.00 0.00 0.02 0.18 0.03 89.39

denovo682 0.00 0.00 0.02 0.25 0.03 89.42

denovo1095 0.00 0.00 0.02 0.18 0.03 89.44

denovo871 0.00 0.00 0.02 0.24 0.03 89.47

denovo1759 0.00 0.00 0.02 0.31 0.03 89.50

HQ326257 0.00 0.00 0.02 0.18 0.03 89.53

denovo898 0.00 0.00 0.02 0.26 0.03 89.56

denovo872 0.00 0.00 0.02 0.25 0.03 89.58

denovo1839 0.00 0.00 0.02 0.18 0.03 89.61

denovo1881 0.00 0.00 0.02 0.25 0.03 89.64

denovo1240 0.00 0.00 0.02 0.25 0.03 89.67

GU563744 0.00 0.00 0.02 0.26 0.03 89.70

denovo1523 0.00 0.00 0.02 0.18 0.03 89.72

denovo1655 0.00 0.00 0.02 0.26 0.03 89.75

denovo1383 0.00 0.00 0.02 0.18 0.03 89.78

denovo225 0.00 0.00 0.02 0.18 0.03 89.80

denovo1489 0.00 0.00 0.02 0.25 0.03 89.83

denovo1150 0.00 0.00 0.02 0.18 0.03 89.86

denovo1203 0.00 0.00 0.02 0.26 0.03 89.88

denovo818 0.00 0.00 0.02 0.18 0.03 89.91

denovo1103 0.00 0.00 0.02 0.24 0.03 89.94

denovo358 0.00 0.00 0.02 0.18 0.03 89.96

denovo147 0.00 0.00 0.02 0.18 0.03 89.99

denovo199 0.00 0.00 0.02 0.18 0.03 90.02

**(B)**

Groups marine & marine_dil

Average dissimilarity = 82.53

Group marine Group marine_dil

Species Av.Abund Av.Abund Av.Diss Diss/SD Contrib% Cum.%

denovo1749 0.97 0.45 9.83 1.24 11.91 11.91

denovo1026 0.18 0.17 3.20 0.98 3.88 15.79

denovo75 0.00 0.15 2.97 0.41 3.60 19.38

JN579972 0.00 0.11 2.40 0.37 2.91 22.30

FJ624884 0.00 0.13 2.28 0.61 2.76 25.06

JN030555 0.00 0.09 2.05 0.31 2.49 27.54

JF937422 0.18 0.13 1.93 1.32 2.33 29.87

GQ118701 0.12 0.05 1.80 0.78 2.18 32.06

denovo1484 0.19 0.05 1.10 0.86 1.33 33.39

AY739689 0.00 0.05 0.96 0.29 1.17 34.56

HQ245988 0.00 0.05 0.87 0.30 1.05 35.61

AY914065 0.00 0.06 0.78 1.18 0.95 36.56

denovo1017 0.25 0.00 0.73 0.39 0.88 37.45

AB299573 0.00 0.04 0.70 0.30 0.85 38.29

HM595366 0.00 0.04 0.67 0.67 0.82 39.11

HE574879 0.02 0.03 0.67 0.74 0.81 39.93

EU935300 0.00 0.05 0.64 1.21 0.77 40.70

denovo1888 0.00 0.04 0.60 0.64 0.73 41.43

U81990 0.00 0.04 0.60 0.45 0.73 42.16

denovo1905 0.25 0.00 0.59 0.31 0.71 42.87

GU940713 0.00 0.04 0.58 0.97 0.71 43.58

EU035954 0.00 0.04 0.57 0.73 0.69 44.27

denovo1146 0.04 0.00 0.55 0.96 0.66 44.93

denovo189 0.04 0.00 0.54 0.89 0.65 45.59

denovo1873 0.00 0.03 0.53 0.43 0.64 46.22

denovo838 0.18 0.00 0.52 0.39 0.63 46.85

denovo756 0.18 0.00 0.48 0.36 0.58 47.43

HQ118426 0.00 0.03 0.47 0.62 0.57 48.00

denovo1273 0.00 0.03 0.46 1.38 0.56 48.56

denovo114 0.18 0.00 0.46 0.34 0.56 49.12

AF286480 0.00 0.02 0.46 0.61 0.55 49.67

DQ889875 0.00 0.04 0.45 0.56 0.54 50.21

AM992178 0.00 0.02 0.44 0.29 0.53 50.74

denovo1476 0.18 0.00 0.43 0.32 0.52 51.26

denovo1791 0.18 0.00 0.42 0.32 0.51 51.78

denovo1322 0.18 0.00 0.42 0.32 0.51 52.29

denovo1720 0.00 0.02 0.42 0.69 0.51 52.80

denovo1743 0.18 0.00 0.42 0.31 0.51 53.31

AJ292596 0.00 0.02 0.41 0.55 0.50 53.81

denovo324 0.18 0.00 0.40 0.30 0.49 54.30

denovo1565 0.18 0.00 0.40 0.30 0.49 54.79

denovo1640 0.18 0.00 0.40 0.30 0.49 55.28

denovo1785 0.18 0.00 0.40 0.30 0.49 55.77

GQ385289 0.18 0.00 0.40 0.30 0.49 56.26

AM935808 0.00 0.03 0.40 0.29 0.48 56.75

AY631057 0.00 0.02 0.39 0.29 0.48 57.22

denovo1022 0.00 0.03 0.37 0.79 0.45 57.67

denovo109 0.02 0.00 0.37 0.81 0.45 58.13

GU118526 0.00 0.03 0.36 0.47 0.44 58.57

AB176201 0.00 0.03 0.36 1.00 0.43 59.00

denovo1249 0.02 0.00 0.36 1.01 0.43 59.43

denovo427 0.02 0.01 0.36 1.01 0.43 59.86

HQ721418 0.00 0.03 0.35 1.04 0.43 60.29

FQ659744 0.00 0.01 0.35 0.29 0.43 60.71

denovo507 0.02 0.01 0.34 0.81 0.41 61.13

denovo1189 0.02 0.00 0.33 0.85 0.40 61.52

denovo1848 0.00 0.02 0.32 0.29 0.39 61.92

denovo605 0.00 0.02 0.32 0.43 0.39 62.31

denovo792 0.00 0.02 0.32 0.42 0.39 62.69

denovo552 0.01 0.01 0.30 0.71 0.36 63.06

denovo547 0.01 0.01 0.30 0.75 0.36 63.42

FJ516885 0.00 0.01 0.29 0.36 0.35 63.77

GU179554 0.00 0.02 0.29 0.88 0.35 64.12

denovo1547 0.00 0.02 0.28 0.52 0.34 64.46

AB477015 0.00 0.02 0.28 1.02 0.34 64.79

X97093 0.00 0.02 0.27 0.92 0.33 65.12

denovo1842 0.01 0.01 0.25 0.90 0.31 65.43

denovo545 0.01 0.00 0.25 1.74 0.30 65.73

AM709702 0.00 0.01 0.25 0.72 0.30 66.03

denovo1162 0.00 0.02 0.25 0.48 0.30 66.33

denovo2019 0.00 0.01 0.25 0.57 0.30 66.63

denovo204 0.01 0.01 0.24 0.92 0.29 66.92

denovo550 0.00 0.01 0.23 0.29 0.28 67.20

JN868782 0.00 0.02 0.22 0.97 0.27 67.47

denovo1923 0.00 0.01 0.22 0.58 0.27 67.74

denovo1070 0.01 0.01 0.22 0.91 0.27 68.01

denovo119 0.01 0.01 0.22 0.68 0.27 68.28

AJ347026 0.00 0.02 0.21 0.52 0.26 68.54

denovo1190 0.00 0.01 0.21 0.60 0.26 68.80

EU160577 0.00 0.02 0.21 0.74 0.26 69.05

AF252321 0.00 0.01 0.21 0.29 0.25 69.31

denovo1263 0.00 0.01 0.20 0.29 0.25 69.55

JN874385 0.01 0.01 0.20 0.64 0.25 69.80

FJ418924 0.00 0.02 0.20 0.52 0.24 70.04

HQ616267 0.00 0.01 0.19 1.16 0.23 70.28

denovo854 0.00 0.01 0.19 0.59 0.23 70.51

denovo822 0.00 0.01 0.19 0.48 0.22 70.73

denovo862 0.00 0.01 0.18 0.37 0.22 70.95

denovo1632 0.00 0.01 0.18 0.72 0.22 71.17

denovo1182 0.00 0.02 0.18 0.52 0.22 71.39

denovo776 0.00 0.02 0.18 0.51 0.22 71.61

denovo607 0.00 0.01 0.18 0.51 0.22 71.83

denovo141 0.01 0.00 0.18 0.82 0.22 72.05

AF170746 0.00 0.02 0.18 0.55 0.21 72.26

EU536078 0.00 0.01 0.16 0.51 0.19 72.45

GU118575 0.00 0.01 0.15 0.56 0.19 72.64

denovo1250 0.00 0.01 0.15 0.71 0.19 72.83

denovo415 0.01 0.00 0.15 0.88 0.18 73.00

HM108471 0.00 0.01 0.15 0.66 0.18 73.18

denovo953 0.01 0.00 0.15 0.62 0.18 73.36

denovo1577 0.00 0.01 0.14 0.61 0.17 73.53

GU553034 0.00 0.01 0.14 0.33 0.17 73.70

denovo1145 0.01 0.00 0.14 1.01 0.17 73.87

denovo1964 0.00 0.01 0.14 0.54 0.16 74.03

denovo61 0.00 0.01 0.14 0.66 0.16 74.20

EU803928 0.00 0.01 0.13 0.50 0.16 74.35

denovo98 0.00 0.01 0.13 0.52 0.15 74.51

GQ921403 0.00 0.01 0.13 0.29 0.15 74.66

denovo1284 0.00 0.01 0.13 0.54 0.15 74.82

FJ892785 0.00 0.01 0.13 0.66 0.15 74.97

JF937433 0.00 0.01 0.12 0.52 0.15 75.12

GU118690 0.00 0.01 0.12 0.54 0.15 75.27

GQ263306 0.00 0.01 0.12 0.66 0.15 75.42

denovo696 0.00 0.01 0.12 0.29 0.15 75.57

denovo663 0.00 0.01 0.12 0.54 0.15 75.72

DQ316817 0.00 0.01 0.12 0.29 0.15 75.86

denovo574 0.00 0.01 0.12 0.45 0.14 76.01

denovo249 0.00 0.01 0.11 0.76 0.14 76.15

GU118606 0.00 0.01 0.11 0.62 0.14 76.28

denovo1420 0.00 0.01 0.11 0.58 0.13 76.42

DQ889881 0.00 0.01 0.11 0.55 0.13 76.55

GU305772 0.00 0.01 0.10 0.72 0.12 76.67

EU463921 0.00 0.01 0.10 0.43 0.12 76.80

FJ900573 0.00 0.01 0.10 0.55 0.12 76.92

denovo430 0.01 0.00 0.10 0.90 0.12 77.04

denovo1462 0.01 0.00 0.10 0.60 0.12 77.16

denovo1479 0.00 0.00 0.10 0.39 0.12 77.28

FJ557790 0.00 0.01 0.10 0.49 0.12 77.39

HQ671075 0.01 0.00 0.10 0.29 0.12 77.51

FJ820465 0.00 0.01 0.09 0.43 0.11 77.62

denovo412 0.00 0.00 0.09 0.47 0.11 77.74

DQ860060 0.00 0.00 0.09 0.57 0.11 77.85

denovo423 0.00 0.01 0.09 0.43 0.11 77.96

JF925031 0.00 0.01 0.09 0.61 0.11 78.07

FJ169195 0.00 0.00 0.09 0.58 0.11 78.18

denovo1961 0.00 0.01 0.09 0.42 0.11 78.29

AY959053 0.00 0.01 0.09 0.62 0.11 78.40

FJ802385 0.00 0.01 0.09 0.54 0.11 78.51

denovo528 0.00 0.01 0.09 0.33 0.11 78.61

denovo305 0.00 0.01 0.09 0.52 0.11 78.72

EF076171 0.00 0.01 0.09 0.61 0.11 78.83

denovo788 0.00 0.01 0.09 0.29 0.10 78.93

denovo917 0.00 0.00 0.09 0.29 0.10 79.03

denovo1480 0.00 0.00 0.09 0.38 0.10 79.14

denovo828 0.00 0.00 0.08 0.48 0.10 79.24

FJ381979 0.00 0.01 0.08 0.54 0.10 79.34

denovo1789 0.00 0.00 0.08 0.48 0.10 79.44

FJ457296 0.00 0.00 0.08 0.33 0.10 79.54

denovo1306 0.00 0.00 0.08 0.40 0.10 79.63

denovo1085 0.00 0.00 0.08 0.60 0.10 79.73

FJ999591 0.00 0.01 0.08 0.48 0.09 79.82

denovo1898 0.00 0.00 0.08 0.49 0.09 79.92

EF018153 0.00 0.00 0.08 0.29 0.09 80.01

denovo520 0.00 0.00 0.08 0.50 0.09 80.10

denovo709 0.00 0.00 0.07 0.65 0.09 80.19

denovo1590 0.00 0.01 0.07 0.54 0.09 80.28

HQ753432 0.00 0.01 0.07 0.51 0.09 80.37

denovo88 0.00 0.00 0.07 0.29 0.09 80.46

denovo1579 0.00 0.00 0.07 0.77 0.09 80.54

denovo2003 0.00 0.01 0.07 0.43 0.09 80.63

denovo1526 0.00 0.00 0.07 0.29 0.09 80.71

denovo744 0.00 0.00 0.07 0.41 0.09 80.80

denovo499 0.00 0.00 0.07 0.29 0.08 80.88

denovo1705 0.00 0.00 0.07 0.29 0.08 80.96

denovo165 0.00 0.01 0.07 0.38 0.08 81.05

denovo308 0.00 0.00 0.07 0.54 0.08 81.13

denovo1414 0.00 0.00 0.07 0.29 0.08 81.21

denovo1093 0.00 0.00 0.07 0.29 0.08 81.29

denovo860 0.00 0.00 0.06 0.29 0.08 81.37

denovo1696 0.00 0.00 0.06 0.29 0.08 81.45

denovo1585 0.00 0.00 0.06 0.42 0.08 81.52

EF076172 0.00 0.01 0.06 0.52 0.08 81.60

FJ529262 0.00 0.01 0.06 0.50 0.08 81.68

denovo1688 0.00 0.00 0.06 0.29 0.08 81.75

denovo1703 0.00 0.00 0.06 0.42 0.07 81.83

denovo916 0.00 0.00 0.06 0.42 0.07 81.90

denovo103 0.00 0.00 0.06 0.29 0.07 81.98

denovo659 0.00 0.00 0.06 0.51 0.07 82.05

denovo1670 0.00 0.00 0.06 0.41 0.07 82.13

denovo1753 0.00 0.00 0.06 0.38 0.07 82.20

AJ621576 0.00 0.01 0.06 0.53 0.07 82.27

HQ672216 0.00 0.00 0.06 0.41 0.07 82.34

FQ660217 0.00 0.00 0.06 0.43 0.07 82.41

HQ118340 0.00 0.00 0.06 0.51 0.07 82.49

denovo1303 0.00 0.01 0.06 0.39 0.07 82.56

denovo521 0.00 0.00 0.06 0.29 0.07 82.63

denovo707 0.00 0.01 0.06 0.42 0.07 82.70

denovo1555 0.00 0.00 0.06 0.39 0.07 82.77

EU817491 0.00 0.00 0.06 0.41 0.07 82.84

denovo6 0.00 0.00 0.06 0.39 0.07 82.91

AJ583166 0.00 0.00 0.06 0.35 0.07 82.98

JN411267 0.00 0.00 0.05 0.42 0.07 83.04

DQ889935 0.00 0.00 0.05 0.43 0.06 83.11

denovo1641 0.00 0.00 0.05 0.29 0.06 83.17

denovo320 0.00 0.00 0.05 0.65 0.06 83.23

denovo43 0.00 0.00 0.05 0.65 0.06 83.30

denovo767 0.00 0.00 0.05 0.29 0.06 83.36

denovo1341 0.00 0.00 0.05 0.29 0.06 83.42

denovo1701 0.00 0.00 0.05 0.66 0.06 83.49

denovo1147 0.00 0.00 0.05 0.36 0.06 83.55

denovo1082 0.00 0.00 0.05 0.29 0.06 83.61

AJ863291 0.00 0.00 0.05 0.29 0.06 83.67

denovo1274 0.00 0.00 0.05 0.29 0.06 83.73

denovo1556 0.00 0.00 0.05 0.29 0.06 83.79

denovo1875 0.00 0.00 0.05 0.29 0.06 83.86

denovo1088 0.00 0.00 0.05 0.54 0.06 83.92

denovo40 0.00 0.00 0.05 0.40 0.06 83.97

denovo1265 0.00 0.00 0.05 0.42 0.06 84.03

AJ633944 0.00 0.00 0.05 0.41 0.06 84.09

DQ396132 0.00 0.00 0.05 0.41 0.06 84.15

denovo898 0.00 0.00 0.05 0.43 0.06 84.21

denovo818 0.00 0.00 0.05 0.29 0.06 84.26

denovo1374 0.00 0.00 0.05 0.41 0.06 84.32

denovo1240 0.00 0.00 0.05 0.41 0.06 84.37

denovo1095 0.00 0.00 0.05 0.29 0.06 84.43

AY922243 0.00 0.00 0.04 0.41 0.05 84.48

denovo1230 0.00 0.00 0.04 0.54 0.05 84.54

denovo624 0.00 0.00 0.04 0.29 0.05 84.59

FJ826409 0.00 0.00 0.04 0.41 0.05 84.65

denovo657 0.00 0.00 0.04 0.29 0.05 84.70

denovo790 0.00 0.00 0.04 0.54 0.05 84.75

denovo727 0.00 0.00 0.04 0.43 0.05 84.81

denovo358 0.00 0.00 0.04 0.29 0.05 84.86

denovo1383 0.00 0.00 0.04 0.29 0.05 84.91

denovo225 0.00 0.00 0.04 0.29 0.05 84.97

GU563744 0.00 0.00 0.04 0.42 0.05 85.02

denovo543 0.00 0.00 0.04 0.40 0.05 85.07

FJ497578 0.00 0.00 0.04 0.43 0.05 85.12

denovo1415 0.00 0.00 0.04 0.40 0.05 85.17

EF153415 0.00 0.00 0.04 0.40 0.05 85.23

denovo28 0.00 0.00 0.04 0.29 0.05 85.28

denovo1299 0.00 0.00 0.04 0.29 0.05 85.33

denovo787 0.00 0.00 0.04 0.29 0.05 85.38

denovo918 0.00 0.00 0.04 0.29 0.05 85.43

FJ946588 0.00 0.00 0.04 0.29 0.05 85.48

GQ903461 0.00 0.00 0.04 0.29 0.05 85.53

denovo1226 0.00 0.00 0.04 0.29 0.05 85.58

denovo819 0.00 0.00 0.04 0.29 0.05 85.63

EU794092 0.00 0.00 0.04 0.42 0.05 85.68

FN666870 0.00 0.00 0.04 0.29 0.05 85.73

denovo1338 0.00 0.00 0.04 0.29 0.05 85.78

FJ675001 0.00 0.01 0.04 0.29 0.05 85.83

denovo244 0.00 0.00 0.04 0.40 0.05 85.87

JF808978 0.00 0.00 0.04 0.42 0.05 85.92

denovo1561 0.00 0.00 0.04 0.29 0.05 85.97

denovo431 0.00 0.00 0.04 0.41 0.05 86.01

denovo769 0.00 0.00 0.04 0.43 0.05 86.06

denovo1292 0.00 0.00 0.04 0.40 0.05 86.11

denovo719 0.00 0.00 0.04 0.38 0.05 86.15

HQ143274 0.00 0.00 0.04 0.29 0.05 86.20

denovo1855 0.00 0.00 0.04 0.29 0.05 86.24

denovo66 0.00 0.00 0.04 0.29 0.05 86.29

denovo1757 0.00 0.00 0.04 0.38 0.04 86.33

denovo1203 0.00 0.00 0.04 0.40 0.04 86.38

denovo1243 0.00 0.00 0.04 0.29 0.04 86.42

denovo1412 0.00 0.00 0.04 0.29 0.04 86.47

denovo1014 0.00 0.00 0.04 0.29 0.04 86.51

denovo993 0.00 0.00 0.04 0.29 0.04 86.56

denovo1139 0.00 0.00 0.04 0.29 0.04 86.60

denovo1443 0.00 0.00 0.04 0.29 0.04 86.65

denovo1911 0.00 0.00 0.04 0.29 0.04 86.69

denovo1937 0.00 0.00 0.04 0.29 0.04 86.73

denovo1998 0.00 0.00 0.04 0.29 0.04 86.78

denovo565 0.00 0.00 0.04 0.29 0.04 86.82

denovo921 0.00 0.00 0.04 0.29 0.04 86.87

denovo104 0.00 0.00 0.04 0.42 0.04 86.91

FJ808721 0.00 0.00 0.04 0.41 0.04 86.96

denovo1775 0.00 0.00 0.04 0.29 0.04 87.00

denovo260 0.00 0.00 0.04 0.29 0.04 87.04

denovo190 0.00 0.00 0.04 0.43 0.04 87.09

denovo1237 0.00 0.00 0.04 0.29 0.04 87.13

denovo1856 0.00 0.00 0.04 0.29 0.04 87.17

denovo236 0.00 0.00 0.04 0.29 0.04 87.21

denovo94 0.00 0.00 0.04 0.29 0.04 87.26

denovo631 0.00 0.00 0.04 0.29 0.04 87.30

EF613488 0.00 0.00 0.03 0.29 0.04 87.34

JN544140 0.00 0.00 0.03 0.43 0.04 87.38

denovo804 0.00 0.00 0.03 0.39 0.04 87.42

denovo432 0.00 0.00 0.03 0.39 0.04 87.46

HQ616309 0.00 0.00 0.03 0.29 0.04 87.50

denovo1423 0.00 0.00 0.03 0.40 0.04 87.54

denovo1457 0.00 0.00 0.03 0.43 0.04 87.58

denovo183 0.00 0.00 0.03 0.29 0.04 87.62

denovo1930 0.00 0.00 0.03 0.39 0.04 87.66

denovo1936 0.00 0.00 0.03 0.29 0.04 87.70

denovo827 0.00 0.00 0.03 0.29 0.04 87.74

denovo2018 0.00 0.00 0.03 0.29 0.04 87.77

denovo1396 0.00 0.00 0.03 0.29 0.04 87.81

denovo385 0.00 0.00 0.03 0.29 0.04 87.85

denovo728 0.00 0.00 0.03 0.29 0.04 87.89

GU118588 0.00 0.00 0.03 0.29 0.04 87.93

EU776368 0.00 0.00 0.03 0.29 0.04 87.96

denovo1391 0.00 0.00 0.03 0.43 0.04 88.00

denovo600 0.00 0.00 0.03 0.29 0.04 88.04

denovo1845 0.00 0.00 0.03 0.29 0.04 88.07

denovo977 0.00 0.00 0.03 0.29 0.04 88.11

denovo1643 0.00 0.00 0.03 0.29 0.04 88.14

denovo593 0.00 0.00 0.03 0.29 0.04 88.18

denovo1110 0.00 0.00 0.03 0.29 0.04 88.21

denovo1141 0.00 0.00 0.03 0.29 0.04 88.25

denovo1227 0.00 0.00 0.03 0.29 0.04 88.29

denovo1254 0.00 0.00 0.03 0.29 0.04 88.32

denovo1264 0.00 0.00 0.03 0.29 0.04 88.36

denovo1309 0.00 0.00 0.03 0.29 0.04 88.39

denovo1340 0.00 0.00 0.03 0.29 0.04 88.43

denovo1407 0.00 0.00 0.03 0.29 0.04 88.46

denovo142 0.00 0.00 0.03 0.29 0.04 88.50

denovo1521 0.00 0.00 0.03 0.29 0.04 88.54

denovo1674 0.00 0.00 0.03 0.29 0.04 88.57

denovo1727 0.00 0.00 0.03 0.29 0.04 88.61

denovo1751 0.00 0.00 0.03 0.29 0.04 88.64

denovo1877 0.00 0.00 0.03 0.29 0.04 88.68

denovo1999 0.00 0.00 0.03 0.29 0.04 88.72

denovo25 0.00 0.00 0.03 0.29 0.04 88.75

denovo468 0.00 0.00 0.03 0.29 0.04 88.79

denovo603 0.00 0.00 0.03 0.29 0.04 88.82

denovo618 0.00 0.00 0.03 0.29 0.04 88.86

denovo717 0.00 0.00 0.03 0.29 0.04 88.89

GU941113 0.00 0.00 0.03 0.29 0.04 88.93

denovo1390 0.00 0.00 0.03 0.29 0.04 88.97

denovo1385 0.00 0.00 0.03 0.29 0.04 89.00

denovo161 0.00 0.00 0.03 0.29 0.04 89.04

denovo332 0.00 0.00 0.03 0.29 0.04 89.07

denovo428 0.00 0.00 0.03 0.29 0.04 89.11

denovo535 0.00 0.00 0.03 0.29 0.04 89.14

denovo2047 0.00 0.00 0.03 0.29 0.04 89.18

FJ957596 0.00 0.00 0.03 0.42 0.04 89.21

FJ848403 0.00 0.00 0.03 0.42 0.03 89.25

HQ757459 0.00 0.00 0.03 0.29 0.03 89.28

denovo1619 0.00 0.00 0.03 0.41 0.03 89.32

denovo1889 0.00 0.00 0.03 0.43 0.03 89.35

denovo853 0.00 0.00 0.03 0.29 0.03 89.39

denovo346 0.00 0.00 0.03 0.43 0.03 89.42

denovo1824 0.00 0.00 0.03 0.29 0.03 89.45

EU794074 0.00 0.00 0.03 0.29 0.03 89.49

denovo198 0.00 0.00 0.03 0.29 0.03 89.52

HQ326290 0.00 0.00 0.03 0.29 0.03 89.55

denovo797 0.00 0.00 0.03 0.29 0.03 89.58

denovo1633 0.00 0.00 0.03 0.29 0.03 89.61

HQ323455 0.00 0.00 0.03 0.29 0.03 89.65

denovo1584 0.00 0.00 0.03 0.29 0.03 89.68

denovo1518 0.00 0.00 0.03 0.29 0.03 89.71

AB084524 0.00 0.00 0.03 0.29 0.03 89.74

denovo1491 0.00 0.00 0.03 0.29 0.03 89.77

FJ269073 0.00 0.00 0.03 0.29 0.03 89.81

denovo2022 0.00 0.00 0.03 0.29 0.03 89.84

AF332511 0.00 0.00 0.03 0.29 0.03 89.87

denovo1764 0.00 0.00 0.03 0.29 0.03 89.90

denovo1940 0.00 0.00 0.03 0.29 0.03 89.93

denovo195 0.00 0.00 0.03 0.29 0.03 89.97

denovo2011 0.00 0.00 0.03 0.29 0.03 90.00

denovo2052 0.00 0.00 0.03 0.29 0.03 90.03

Groups marine & mueller_hinton

Average dissimilarity = 63.17

Group marine Group mueller_hinton

Species Av.Abund Av.Abund Av.Diss Diss/SD Contrib% Cum.%

denovo1749 0.97 0.60 8.99 0.79 14.23 14.23

GQ118701 0.12 0.03 2.68 0.80 4.25 18.48

FJ516885 0.00 0.08 2.62 0.29 4.15 22.64

denovo1026 0.18 0.12 2.58 0.87 4.08 26.72

HM595366 0.00 0.13 2.32 0.55 3.68 30.39

JN579972 0.00 0.08 2.23 0.30 3.54 33.93

denovo1888 0.00 0.12 2.11 0.56 3.34 37.27

HE574879 0.02 0.07 1.66 0.64 2.63 39.90

U81990 0.00 0.07 1.31 0.55 2.07 41.97

denovo1484 0.19 0.04 1.28 0.86 2.03 44.00

JF937422 0.18 0.03 0.93 0.71 1.47 45.47

denovo189 0.04 0.00 0.76 0.93 1.20 46.67

denovo605 0.00 0.04 0.75 0.41 1.19 47.86

denovo1146 0.04 0.00 0.75 1.02 1.19 49.05

denovo1017 0.25 0.00 0.75 0.38 1.18 50.23

denovo552 0.01 0.03 0.72 0.59 1.15 51.37

denovo114 0.18 0.01 0.66 0.46 1.04 52.41

denovo1905 0.25 0.00 0.63 0.31 0.99 53.40

denovo862 0.00 0.03 0.60 0.43 0.95 54.35

denovo838 0.18 0.00 0.58 0.42 0.92 55.27

denovo427 0.02 0.01 0.54 0.95 0.86 56.14

denovo109 0.02 0.00 0.54 0.82 0.85 56.98

denovo1961 0.00 0.03 0.52 0.54 0.82 57.81

denovo547 0.01 0.01 0.51 0.80 0.81 58.62

denovo1243 0.00 0.03 0.50 0.53 0.79 59.41

denovo1249 0.02 0.00 0.49 1.10 0.78 60.19

denovo756 0.18 0.00 0.48 0.34 0.76 60.95

denovo1791 0.18 0.00 0.48 0.34 0.76 61.71

denovo507 0.02 0.00 0.47 0.76 0.74 62.46

denovo1189 0.02 0.00 0.46 0.91 0.74 63.19

denovo1476 0.18 0.00 0.46 0.33 0.73 63.92

denovo1322 0.18 0.00 0.45 0.32 0.72 64.63

denovo1743 0.18 0.00 0.45 0.32 0.71 65.35

denovo324 0.18 0.00 0.42 0.30 0.67 66.02

denovo1565 0.18 0.00 0.42 0.30 0.67 66.69

denovo1640 0.18 0.00 0.42 0.30 0.67 67.36

denovo1785 0.18 0.00 0.42 0.30 0.67 68.04

GQ385289 0.18 0.00 0.42 0.30 0.67 68.71

AB176201 0.00 0.02 0.40 0.58 0.64 69.35

denovo119 0.01 0.01 0.34 0.75 0.53 69.88

denovo1022 0.00 0.02 0.32 0.40 0.51 70.39

JN874385 0.01 0.01 0.31 0.49 0.50 70.89

denovo1070 0.01 0.01 0.31 0.84 0.49 71.38

denovo545 0.01 0.01 0.28 1.48 0.44 71.82

HQ323455 0.00 0.01 0.27 0.29 0.43 72.26

denovo854 0.00 0.01 0.27 0.48 0.43 72.69

denovo204 0.01 0.00 0.27 0.70 0.42 73.11

denovo141 0.01 0.00 0.26 0.85 0.41 73.51

EU536078 0.00 0.01 0.26 0.48 0.41 73.92

denovo1129 0.00 0.01 0.26 0.55 0.40 74.32

denovo1703 0.00 0.01 0.24 0.49 0.38 74.70

denovo415 0.01 0.01 0.24 1.06 0.37 75.07

denovo1412 0.00 0.01 0.23 0.41 0.36 75.43

denovo1273 0.00 0.01 0.22 0.46 0.35 75.78

denovo953 0.01 0.00 0.22 0.55 0.35 76.13

denovo1753 0.00 0.01 0.22 0.33 0.34 76.47

denovo927 0.00 0.01 0.21 0.42 0.33 76.80

denovo1145 0.01 0.00 0.20 1.04 0.32 77.12

denovo1491 0.00 0.01 0.19 0.29 0.31 77.43

FJ849072 0.00 0.01 0.19 0.29 0.31 77.73

AF170746 0.00 0.01 0.18 0.40 0.29 78.02

denovo1096 0.00 0.01 0.18 0.41 0.29 78.31

denovo1374 0.00 0.01 0.18 0.33 0.28 78.59

denovo1462 0.01 0.00 0.17 0.67 0.27 78.86

HM108471 0.00 0.01 0.17 0.58 0.27 79.13

AY914065 0.00 0.01 0.16 0.81 0.26 79.39

denovo1842 0.01 0.00 0.16 0.73 0.25 79.63

JF802167 0.00 0.01 0.15 0.33 0.24 79.88

denovo95 0.00 0.01 0.15 0.29 0.24 80.12

AF286480 0.00 0.01 0.15 0.36 0.24 80.35

denovo767 0.00 0.01 0.15 0.42 0.23 80.59

denovo1873 0.00 0.00 0.15 0.40 0.23 80.82

denovo1479 0.00 0.00 0.15 0.41 0.23 81.05

denovo430 0.01 0.00 0.14 0.92 0.23 81.28

HQ616267 0.00 0.00 0.14 0.84 0.23 81.51

denovo6 0.00 0.00 0.14 0.36 0.23 81.73

JF937433 0.00 0.01 0.14 0.41 0.22 81.96

denovo1446 0.00 0.01 0.14 0.42 0.22 82.18

HQ671075 0.01 0.00 0.14 0.29 0.22 82.40

GU940713 0.00 0.01 0.14 0.29 0.22 82.61

denovo916 0.00 0.01 0.14 0.29 0.22 82.83

denovo1570 0.00 0.01 0.14 0.29 0.22 83.04

denovo2056 0.00 0.01 0.14 0.29 0.22 83.26

denovo757 0.00 0.01 0.14 0.29 0.22 83.48

JQ032050 0.00 0.01 0.14 0.29 0.22 83.69

denovo709 0.00 0.00 0.14 0.72 0.21 83.91

denovo2029 0.00 0.01 0.13 0.40 0.21 84.11

denovo1341 0.00 0.01 0.13 0.41 0.20 84.32

denovo1085 0.00 0.00 0.13 0.61 0.20 84.52

denovo2003 0.00 0.01 0.12 0.42 0.20 84.71

denovo1921 0.00 0.01 0.12 0.29 0.19 84.91

AM709702 0.00 0.00 0.12 0.29 0.19 85.10

FJ169195 0.00 0.00 0.12 0.52 0.19 85.29

AB294294 0.00 0.00 0.12 0.36 0.18 85.47

denovo1579 0.00 0.00 0.11 0.89 0.18 85.65

EU935300 0.00 0.00 0.11 0.68 0.18 85.83

GQ433928 0.00 0.00 0.10 0.29 0.16 85.99

denovo1789 0.00 0.00 0.10 0.40 0.16 86.15

denovo1619 0.00 0.00 0.10 0.48 0.15 86.30

denovo679 0.00 0.00 0.09 0.35 0.15 86.45

DQ860060 0.00 0.00 0.09 0.50 0.14 86.58

denovo706 0.00 0.00 0.09 0.29 0.14 86.72

denovo639 0.00 0.00 0.09 0.29 0.14 86.86

FN396956 0.00 0.00 0.09 0.29 0.14 87.00

denovo1500 0.00 0.00 0.08 0.29 0.13 87.13

denovo43 0.00 0.00 0.08 0.71 0.13 87.27

denovo320 0.00 0.00 0.08 0.71 0.13 87.40

denovo190 0.00 0.00 0.08 0.51 0.13 87.52

denovo1292 0.00 0.00 0.08 0.50 0.12 87.65

AJ240982 0.00 0.00 0.08 0.29 0.12 87.77

denovo1701 0.00 0.00 0.08 0.67 0.12 87.89

denovo828 0.00 0.00 0.08 0.38 0.12 88.01

denovo790 0.00 0.00 0.07 0.61 0.12 88.13

denovo1088 0.00 0.00 0.07 0.54 0.12 88.24

denovo1577 0.00 0.00 0.07 0.29 0.11 88.36

GU179554 0.00 0.00 0.07 0.52 0.11 88.47

denovo1131 0.00 0.00 0.07 0.29 0.10 88.57

denovo1230 0.00 0.00 0.07 0.55 0.10 88.68

HE574926 0.00 0.00 0.06 0.38 0.10 88.78

denovo1390 0.00 0.00 0.06 0.40 0.10 88.88

denovo657 0.00 0.00 0.06 0.29 0.10 88.98

denovo1132 0.00 0.00 0.06 0.29 0.10 89.08

X97093 0.00 0.00 0.06 0.55 0.10 89.18

HQ588359 0.00 0.00 0.06 0.29 0.09 89.27

denovo1338 0.00 0.00 0.06 0.29 0.09 89.37

denovo1832 0.00 0.00 0.06 0.52 0.09 89.46

denovo1420 0.00 0.00 0.06 0.51 0.09 89.55

denovo520 0.00 0.00 0.06 0.29 0.09 89.64

denovo1480 0.00 0.00 0.05 0.29 0.09 89.72

denovo990 0.00 0.00 0.05 0.29 0.09 89.81

denovo719 0.00 0.00 0.05 0.39 0.09 89.90

HQ721418 0.00 0.00 0.05 0.41 0.08 89.98

denovo815 0.00 0.00 0.05 0.39 0.08 90.06

Groups marine_dil & mueller_hinton

Average dissimilarity = 83.47

Group marine_dil Group mueller_hinton

Species Av.Abund Av.Abund Av.Diss Diss/SD Contrib% Cum.%

denovo1749 0.45 0.60 8.44 1.23 10.11 10.11

JN579972 0.11 0.08 3.42 0.48 4.09 14.20

denovo1026 0.17 0.12 3.08 1.05 3.68 17.88

denovo75 0.15 0.00 2.92 0.42 3.50 21.39

FJ624884 0.13 0.00 2.27 0.64 2.71 24.10

HM595366 0.04 0.13 2.16 0.77 2.58 26.68

JN030555 0.09 0.00 2.02 0.32 2.41 29.10

denovo1888 0.04 0.12 1.92 0.76 2.30 31.40

FJ516885 0.01 0.08 1.87 0.34 2.24 33.64

JF937422 0.13 0.03 1.37 1.13 1.64 35.28

U81990 0.04 0.07 1.33 0.72 1.59 36.87

HE574879 0.03 0.07 1.28 0.66 1.53 38.40

AY739689 0.05 0.00 0.96 0.30 1.15 39.55

HQ245988 0.05 0.00 0.88 0.32 1.06 40.61

GQ118701 0.05 0.03 0.84 1.05 1.00 41.61

denovo1484 0.05 0.04 0.84 1.16 1.00 42.61

denovo605 0.02 0.04 0.81 0.59 0.97 43.57

AY914065 0.06 0.01 0.73 1.16 0.88 44.45

AB299573 0.04 0.00 0.70 0.31 0.84 45.29

EU935300 0.05 0.00 0.62 1.25 0.74 46.03

GU940713 0.04 0.01 0.61 1.06 0.73 46.76

denovo862 0.01 0.03 0.59 0.56 0.71 47.47

denovo1873 0.03 0.00 0.58 0.51 0.69 48.16

EU035954 0.04 0.00 0.57 0.76 0.68 48.84

denovo552 0.01 0.03 0.53 0.59 0.63 49.47

denovo1022 0.03 0.02 0.49 0.82 0.59 50.06

AF286480 0.02 0.01 0.48 0.71 0.58 50.64

HQ118426 0.03 0.00 0.47 0.64 0.56 51.20

denovo1273 0.03 0.01 0.47 1.38 0.56 51.76

DQ889875 0.04 0.00 0.46 0.61 0.56 52.32

AB176201 0.03 0.02 0.46 1.02 0.55 52.87

denovo1961 0.01 0.03 0.45 0.67 0.54 53.40

AM992178 0.02 0.00 0.43 0.29 0.51 53.91

denovo1720 0.02 0.00 0.42 0.71 0.50 54.41

AJ292596 0.02 0.00 0.41 0.57 0.49 54.90

AM935808 0.03 0.00 0.40 0.30 0.48 55.38

denovo1243 0.00 0.03 0.40 0.59 0.48 55.86

AY631057 0.02 0.00 0.39 0.30 0.47 56.33

GU118526 0.03 0.00 0.37 0.49 0.44 56.77

HQ721418 0.03 0.00 0.35 1.09 0.42 57.19

FQ659744 0.01 0.00 0.34 0.29 0.41 57.60

denovo1848 0.02 0.00 0.33 0.30 0.39 57.99

denovo792 0.02 0.00 0.32 0.43 0.38 58.37

denovo1547 0.02 0.00 0.30 0.58 0.35 58.73

GU179554 0.02 0.00 0.29 1.01 0.35 59.08

AB477015 0.02 0.00 0.28 1.07 0.33 59.42

EU536078 0.01 0.01 0.28 0.70 0.33 59.75

denovo854 0.01 0.01 0.27 0.67 0.33 60.08

X97093 0.02 0.00 0.27 0.87 0.32 60.40

denovo427 0.01 0.01 0.26 0.64 0.31 60.71

denovo547 0.01 0.01 0.26 0.61 0.31 61.02

denovo1162 0.02 0.00 0.26 0.53 0.31 61.33

AF170746 0.02 0.01 0.25 0.60 0.30 61.64

denovo2019 0.01 0.00 0.24 0.59 0.29 61.93

denovo1842 0.01 0.00 0.24 0.79 0.29 62.22

denovo1923 0.01 0.00 0.24 0.65 0.28 62.50

denovo550 0.01 0.00 0.23 0.30 0.27 62.78

HQ323455 0.00 0.01 0.22 0.33 0.27 63.05

JN868782 0.02 0.00 0.22 1.04 0.27 63.31

denovo1070 0.01 0.01 0.22 0.84 0.27 63.58

denovo1703 0.00 0.01 0.22 0.62 0.26 63.84

AJ347026 0.02 0.00 0.22 0.54 0.26 64.10

denovo119 0.01 0.01 0.21 0.57 0.26 64.35

EU160577 0.02 0.00 0.21 0.76 0.25 64.61

denovo1190 0.01 0.00 0.21 0.62 0.25 64.86

AF252321 0.01 0.00 0.21 0.30 0.25 65.11

JN874385 0.01 0.01 0.20 0.54 0.25 65.36

denovo1263 0.01 0.00 0.20 0.30 0.24 65.60

HM108471 0.01 0.01 0.20 0.78 0.24 65.84

FJ418924 0.02 0.00 0.20 0.53 0.24 66.08

denovo1412 0.00 0.01 0.20 0.49 0.24 66.32

JF937433 0.01 0.01 0.20 0.69 0.24 66.56

AM709702 0.01 0.00 0.20 0.71 0.24 66.79

denovo1129 0.00 0.01 0.19 0.56 0.23 67.02

HQ616267 0.01 0.00 0.19 1.04 0.23 67.25

denovo114 0.00 0.01 0.19 0.51 0.22 67.48

denovo607 0.01 0.00 0.19 0.56 0.22 67.70

denovo822 0.01 0.00 0.18 0.50 0.22 67.92

denovo1182 0.02 0.00 0.18 0.53 0.22 68.14

denovo776 0.02 0.00 0.18 0.52 0.22 68.36

denovo1753 0.00 0.01 0.18 0.37 0.22 68.57

denovo1632 0.01 0.00 0.18 0.72 0.21 68.79

denovo204 0.01 0.00 0.18 0.74 0.21 69.00

denovo1491 0.00 0.01 0.17 0.34 0.20 69.20

denovo1577 0.01 0.00 0.16 0.70 0.20 69.39

denovo927 0.00 0.01 0.16 0.42 0.19 69.58

denovo1250 0.01 0.00 0.15 0.74 0.18 69.76

denovo916 0.00 0.01 0.15 0.45 0.18 69.95

denovo767 0.00 0.01 0.15 0.52 0.18 70.13

denovo2003 0.01 0.01 0.15 0.61 0.18 70.30

GU118575 0.01 0.00 0.14 0.53 0.17 70.48

denovo61 0.01 0.00 0.14 0.70 0.17 70.65

FJ849072 0.00 0.01 0.14 0.29 0.17 70.82

GU553034 0.01 0.00 0.14 0.34 0.17 70.99

denovo1096 0.00 0.01 0.14 0.41 0.17 71.15

denovo1341 0.00 0.01 0.14 0.51 0.16 71.32

denovo1964 0.01 0.00 0.14 0.56 0.16 71.48

denovo1017 0.00 0.00 0.13 0.47 0.16 71.64

FJ892785 0.01 0.00 0.13 0.73 0.16 71.80

EU803928 0.01 0.00 0.13 0.52 0.16 71.96

denovo98 0.01 0.00 0.13 0.54 0.15 72.11

denovo1284 0.01 0.00 0.13 0.55 0.15 72.27

GU118690 0.01 0.00 0.13 0.56 0.15 72.42

GQ263306 0.01 0.00 0.12 0.69 0.15 72.56

denovo953 0.00 0.00 0.12 0.49 0.15 72.71

GQ921403 0.01 0.00 0.12 0.29 0.15 72.86

denovo1374 0.00 0.01 0.12 0.36 0.15 73.01

denovo415 0.00 0.01 0.12 0.79 0.15 73.16

denovo696 0.01 0.00 0.12 0.30 0.15 73.30

denovo663 0.01 0.00 0.12 0.55 0.15 73.45

denovo545 0.00 0.01 0.12 0.71 0.15 73.59

DQ316817 0.01 0.00 0.12 0.30 0.14 73.74

denovo574 0.01 0.00 0.12 0.47 0.14 73.88

denovo249 0.01 0.00 0.12 0.86 0.14 74.02

denovo1420 0.01 0.00 0.12 0.65 0.14 74.16

denovo1480 0.00 0.00 0.12 0.49 0.14 74.30

denovo95 0.00 0.01 0.11 0.29 0.13 74.44

JF802167 0.00 0.01 0.11 0.33 0.13 74.57

DQ889881 0.01 0.00 0.11 0.57 0.13 74.70

denovo1446 0.00 0.01 0.11 0.42 0.13 74.83

denovo6 0.00 0.00 0.10 0.40 0.12 74.96

GU118606 0.01 0.00 0.10 0.56 0.12 75.08

EU463921 0.01 0.00 0.10 0.44 0.12 75.20

FJ900573 0.01 0.00 0.10 0.57 0.12 75.32

GU305772 0.01 0.00 0.10 0.73 0.12 75.44

denovo1570 0.00 0.01 0.10 0.29 0.12 75.57

denovo2056 0.00 0.01 0.10 0.29 0.12 75.69

denovo757 0.00 0.01 0.10 0.29 0.12 75.81

JQ032050 0.00 0.01 0.10 0.29 0.12 75.93

denovo2029 0.00 0.01 0.10 0.41 0.12 76.05

FJ557790 0.01 0.00 0.10 0.51 0.12 76.16

EF076171 0.01 0.00 0.10 0.70 0.12 76.28

denovo1249 0.00 0.00 0.10 0.58 0.11 76.39

FJ820465 0.01 0.00 0.09 0.44 0.11 76.51

denovo423 0.01 0.00 0.09 0.44 0.11 76.62

denovo412 0.00 0.00 0.09 0.48 0.11 76.73

denovo1921 0.00 0.01 0.09 0.29 0.11 76.84

JF925031 0.01 0.00 0.09 0.63 0.11 76.95

FJ802385 0.01 0.00 0.09 0.56 0.11 77.06

denovo528 0.01 0.00 0.09 0.34 0.11 77.16

denovo305 0.01 0.00 0.09 0.54 0.11 77.27

denovo657 0.00 0.00 0.09 0.42 0.10 77.37

denovo788 0.01 0.00 0.09 0.30 0.10 77.47

denovo838 0.00 0.00 0.09 0.72 0.10 77.58

DQ860060 0.00 0.00 0.09 0.52 0.10 77.68

denovo917 0.00 0.00 0.08 0.29 0.10 77.78

FJ381979 0.01 0.00 0.08 0.56 0.10 77.88

AY959053 0.01 0.00 0.08 0.57 0.10 77.98

GQ433928 0.00 0.00 0.08 0.35 0.10 78.08

denovo1306 0.00 0.00 0.08 0.41 0.09 78.17

FJ999591 0.01 0.00 0.08 0.49 0.09 78.26

AB294294 0.00 0.00 0.08 0.36 0.09 78.36

denovo1146 0.00 0.00 0.08 0.51 0.09 78.45

denovo507 0.01 0.00 0.08 0.44 0.09 78.54

EF018153 0.00 0.00 0.08 0.30 0.09 78.63

denovo1590 0.01 0.00 0.07 0.55 0.09 78.72

HQ753432 0.01 0.00 0.07 0.53 0.09 78.81

denovo88 0.00 0.00 0.07 0.30 0.09 78.89

denovo1479 0.00 0.00 0.07 0.41 0.09 78.98

FJ457296 0.00 0.00 0.07 0.30 0.08 79.06

AJ240982 0.00 0.00 0.07 0.38 0.08 79.15

denovo744 0.00 0.00 0.07 0.42 0.08 79.23

denovo499 0.00 0.00 0.07 0.30 0.08 79.31

denovo1526 0.00 0.00 0.07 0.29 0.08 79.39

denovo165 0.01 0.00 0.07 0.39 0.08 79.48

denovo1898 0.00 0.00 0.07 0.44 0.08 79.56

denovo1705 0.00 0.00 0.07 0.30 0.08 79.64

denovo308 0.00 0.00 0.07 0.56 0.08 79.72

denovo1414 0.00 0.00 0.07 0.30 0.08 79.80

denovo756 0.00 0.00 0.07 0.52 0.08 79.88

denovo1619 0.00 0.00 0.06 0.49 0.08 79.96

denovo1696 0.00 0.00 0.06 0.30 0.08 80.03

denovo706 0.00 0.00 0.06 0.29 0.08 80.11

denovo639 0.00 0.00 0.06 0.29 0.08 80.19

FN396956 0.00 0.00 0.06 0.29 0.08 80.27

denovo1093 0.00 0.00 0.06 0.29 0.08 80.34

denovo1585 0.00 0.00 0.06 0.43 0.08 80.42

EF076172 0.01 0.00 0.06 0.54 0.08 80.50

denovo1500 0.00 0.00 0.06 0.29 0.08 80.57

denovo860 0.00 0.00 0.06 0.29 0.08 80.65

FJ529262 0.01 0.00 0.06 0.52 0.08 80.72

FJ497578 0.00 0.00 0.06 0.53 0.07 80.80

denovo2018 0.00 0.00 0.06 0.42 0.07 80.87

denovo659 0.00 0.00 0.06 0.52 0.07 80.95

denovo103 0.00 0.00 0.06 0.30 0.07 81.02

denovo1688 0.00 0.00 0.06 0.29 0.07 81.09

denovo1670 0.00 0.00 0.06 0.42 0.07 81.17

AJ621576 0.01 0.00 0.06 0.55 0.07 81.24

denovo707 0.01 0.00 0.06 0.43 0.07 81.31

denovo679 0.00 0.00 0.06 0.36 0.07 81.38

denovo1303 0.01 0.00 0.06 0.40 0.07 81.45

HQ672216 0.00 0.00 0.06 0.42 0.07 81.52

HQ118340 0.00 0.00 0.06 0.53 0.07 81.59

denovo1555 0.00 0.00 0.06 0.40 0.07 81.66

FQ660217 0.00 0.00 0.06 0.44 0.07 81.73

EU817491 0.00 0.00 0.06 0.42 0.07 81.80

GQ903461 0.00 0.00 0.06 0.40 0.07 81.87

denovo521 0.00 0.00 0.06 0.29 0.07 81.94

denovo1014 0.00 0.00 0.06 0.41 0.07 82.01

denovo993 0.00 0.00 0.06 0.41 0.07 82.07

AJ583166 0.00 0.00 0.06 0.36 0.07 82.14

denovo28 0.00 0.00 0.06 0.39 0.07 82.21

JN411267 0.00 0.00 0.05 0.43 0.06 82.27

DQ889935 0.00 0.00 0.05 0.44 0.06 82.34

EF153415 0.00 0.00 0.05 0.50 0.06 82.40

denovo1641 0.00 0.00 0.05 0.30 0.06 82.46

denovo1147 0.00 0.00 0.05 0.37 0.06 82.53

denovo1082 0.00 0.00 0.05 0.30 0.06 82.59

denovo1556 0.00 0.00 0.05 0.30 0.06 82.65

denovo1875 0.00 0.00 0.05 0.30 0.06 82.71

AJ863291 0.00 0.00 0.05 0.29 0.06 82.77

denovo1274 0.00 0.00 0.05 0.29 0.06 82.83

denovo1132 0.00 0.00 0.05 0.29 0.06 82.88

denovo1085 0.00 0.00 0.05 0.41 0.06 82.94

denovo40 0.00 0.00 0.05 0.41 0.06 83.00

denovo1265 0.00 0.00 0.05 0.44 0.06 83.06

denovo898 0.00 0.00 0.05 0.44 0.06 83.11

denovo1561 0.00 0.00 0.05 0.37 0.06 83.17

HQ326290 0.00 0.00 0.05 0.42 0.06 83.23

JF808978 0.00 0.00 0.05 0.52 0.06 83.28

denovo1240 0.00 0.00 0.05 0.42 0.06 83.34

denovo690 0.00 0.00 0.05 0.42 0.06 83.39

denovo1095 0.00 0.00 0.05 0.30 0.05 83.45

denovo818 0.00 0.00 0.05 0.29 0.05 83.50

denovo520 0.00 0.00 0.04 0.44 0.05 83.56

FJ826409 0.00 0.00 0.04 0.42 0.05 83.61

denovo1131 0.00 0.00 0.04 0.29 0.05 83.66

FJ808721 0.00 0.00 0.04 0.52 0.05 83.71

GU563744 0.00 0.00 0.04 0.43 0.05 83.77

denovo543 0.00 0.00 0.04 0.41 0.05 83.82

denovo1383 0.00 0.00 0.04 0.30 0.05 83.87

denovo225 0.00 0.00 0.04 0.30 0.05 83.92

denovo624 0.00 0.00 0.04 0.29 0.05 83.98

denovo727 0.00 0.00 0.04 0.44 0.05 84.03

denovo189 0.00 0.00 0.04 0.40 0.05 84.08

denovo1415 0.00 0.00 0.04 0.41 0.05 84.13

AB176169 0.00 0.00 0.04 0.36 0.05 84.18

denovo358 0.00 0.00 0.04 0.29 0.05 84.23

denovo990 0.00 0.00 0.04 0.29 0.05 84.28

denovo1299 0.00 0.00 0.04 0.30 0.05 84.33

denovo787 0.00 0.00 0.04 0.30 0.05 84.38

denovo918 0.00 0.00 0.04 0.30 0.05 84.43

FJ946588 0.00 0.00 0.04 0.30 0.05 84.48

FJ957596 0.00 0.00 0.04 0.53 0.05 84.53

FJ675001 0.01 0.00 0.04 0.30 0.05 84.58

EU794092 0.00 0.00 0.04 0.43 0.05 84.63

denovo828 0.00 0.00 0.04 0.30 0.05 84.68

denovo1226 0.00 0.00 0.04 0.29 0.05 84.73

denovo819 0.00 0.00 0.04 0.29 0.05 84.78

FN666870 0.00 0.00 0.04 0.30 0.05 84.83

denovo244 0.00 0.00 0.04 0.41 0.05 84.87

denovo1690 0.00 0.00 0.04 0.42 0.05 84.92

HQ588359 0.00 0.00 0.04 0.29 0.05 84.97

denovo769 0.00 0.00 0.04 0.44 0.05 85.02

denovo1189 0.00 0.00 0.04 0.41 0.05 85.06

denovo431 0.00 0.00 0.04 0.42 0.05 85.11

denovo1579 0.00 0.00 0.04 0.54 0.05 85.15

HQ143274 0.00 0.00 0.04 0.30 0.04 85.20

denovo1855 0.00 0.00 0.04 0.30 0.04 85.24

denovo66 0.00 0.00 0.04 0.30 0.04 85.29

denovo771 0.00 0.00 0.04 0.41 0.04 85.33

denovo1757 0.00 0.00 0.04 0.39 0.04 85.38

denovo1139 0.00 0.00 0.04 0.30 0.04 85.42

denovo1443 0.00 0.00 0.04 0.30 0.04 85.46

denovo1911 0.00 0.00 0.04 0.30 0.04 85.51

denovo1937 0.00 0.00 0.04 0.30 0.04 85.55

denovo1998 0.00 0.00 0.04 0.30 0.04 85.60

denovo565 0.00 0.00 0.04 0.30 0.04 85.64

denovo921 0.00 0.00 0.04 0.30 0.04 85.68

denovo104 0.00 0.00 0.04 0.43 0.04 85.73

denovo109 0.00 0.00 0.04 0.30 0.04 85.77

denovo1237 0.00 0.00 0.04 0.30 0.04 85.81

denovo1856 0.00 0.00 0.04 0.30 0.04 85.85

denovo236 0.00 0.00 0.04 0.30 0.04 85.90

denovo94 0.00 0.00 0.04 0.30 0.04 85.94

denovo631 0.00 0.00 0.04 0.30 0.04 85.98

denovo1775 0.00 0.00 0.04 0.29 0.04 86.02

denovo260 0.00 0.00 0.04 0.29 0.04 86.07

denovo87 0.00 0.00 0.03 0.29 0.04 86.11

denovo147 0.00 0.00 0.03 0.29 0.04 86.15

denovo199 0.00 0.00 0.03 0.29 0.04 86.19

EU539909 0.00 0.00 0.03 0.29 0.04 86.23

JF712668 0.00 0.00 0.03 0.41 0.04 86.27

denovo812 0.00 0.00 0.03 0.40 0.04 86.31

JN544140 0.00 0.00 0.03 0.44 0.04 86.35

denovo804 0.00 0.00 0.03 0.40 0.04 86.39

denovo432 0.00 0.00 0.03 0.40 0.04 86.43

HQ616309 0.00 0.00 0.03 0.30 0.04 86.47

DQ298271 0.00 0.00 0.03 0.40 0.04 86.51

denovo34 0.00 0.00 0.03 0.29 0.04 86.55

HQ326257 0.00 0.00 0.03 0.29 0.04 86.59

denovo871 0.00 0.00 0.03 0.40 0.04 86.63

denovo636 0.00 0.00 0.03 0.40 0.04 86.67

denovo1462 0.00 0.00 0.03 0.29 0.04 86.71

HE574926 0.00 0.00 0.03 0.29 0.04 86.75

denovo1930 0.00 0.00 0.03 0.41 0.04 86.79

denovo1457 0.00 0.00 0.03 0.44 0.04 86.83

GU118588 0.00 0.00 0.03 0.30 0.04 86.86

EU776368 0.00 0.00 0.03 0.30 0.04 86.90

FJ203135 0.00 0.00 0.03 0.53 0.04 86.94

denovo827 0.00 0.00 0.03 0.30 0.04 86.98

denovo1396 0.00 0.00 0.03 0.30 0.04 87.01

denovo1391 0.00 0.00 0.03 0.44 0.04 87.05

denovo1936 0.00 0.00 0.03 0.29 0.04 87.09

denovo385 0.00 0.00 0.03 0.29 0.04 87.12

denovo728 0.00 0.00 0.03 0.29 0.04 87.16

denovo709 0.00 0.00 0.03 0.29 0.04 87.20

denovo2047 0.00 0.00 0.03 0.30 0.04 87.23

denovo600 0.00 0.00 0.03 0.30 0.04 87.27

denovo1845 0.00 0.00 0.03 0.30 0.04 87.30

denovo977 0.00 0.00 0.03 0.30 0.04 87.34

denovo1643 0.00 0.00 0.03 0.30 0.04 87.37

denovo593 0.00 0.00 0.03 0.30 0.04 87.41

denovo1110 0.00 0.00 0.03 0.30 0.04 87.44

denovo1141 0.00 0.00 0.03 0.30 0.04 87.48

denovo1227 0.00 0.00 0.03 0.30 0.04 87.52

denovo1254 0.00 0.00 0.03 0.30 0.04 87.55

denovo1264 0.00 0.00 0.03 0.30 0.04 87.59

denovo1309 0.00 0.00 0.03 0.30 0.04 87.62

denovo1340 0.00 0.00 0.03 0.30 0.04 87.66

denovo1407 0.00 0.00 0.03 0.30 0.04 87.69

denovo142 0.00 0.00 0.03 0.30 0.04 87.73

denovo1521 0.00 0.00 0.03 0.30 0.04 87.76

denovo1674 0.00 0.00 0.03 0.30 0.04 87.80

denovo1727 0.00 0.00 0.03 0.30 0.04 87.84

denovo1751 0.00 0.00 0.03 0.30 0.04 87.87

denovo1877 0.00 0.00 0.03 0.30 0.04 87.91

denovo1999 0.00 0.00 0.03 0.30 0.04 87.94

denovo25 0.00 0.00 0.03 0.30 0.04 87.98

denovo468 0.00 0.00 0.03 0.30 0.04 88.01

denovo603 0.00 0.00 0.03 0.30 0.04 88.05

denovo618 0.00 0.00 0.03 0.30 0.04 88.08

denovo717 0.00 0.00 0.03 0.30 0.04 88.12

GU941113 0.00 0.00 0.03 0.30 0.04 88.16

denovo1150 0.00 0.00 0.03 0.29 0.03 88.19

HQ757459 0.00 0.00 0.03 0.30 0.03 88.22

denovo1385 0.00 0.00 0.03 0.29 0.03 88.26

denovo161 0.00 0.00 0.03 0.29 0.03 88.29

denovo332 0.00 0.00 0.03 0.29 0.03 88.33

denovo428 0.00 0.00 0.03 0.29 0.03 88.36

denovo535 0.00 0.00 0.03 0.29 0.03 88.40

denovo1824 0.00 0.00 0.03 0.30 0.03 88.43

EU794074 0.00 0.00 0.03 0.30 0.03 88.46

denovo859 0.00 0.00 0.03 0.42 0.03 88.50

denovo1774 0.00 0.00 0.03 0.41 0.03 88.53

denovo1401 0.00 0.00 0.03 0.43 0.03 88.56

denovo1467 0.00 0.00 0.03 0.43 0.03 88.59

EU844911 0.00 0.00 0.03 0.43 0.03 88.62

JL812080 0.00 0.00 0.03 0.43 0.03 88.66

AJ867671 0.00 0.00 0.03 0.41 0.03 88.69

denovo198 0.00 0.00 0.03 0.30 0.03 88.72

denovo797 0.00 0.00 0.03 0.30 0.03 88.75

denovo1633 0.00 0.00 0.03 0.30 0.03 88.78

denovo1584 0.00 0.00 0.03 0.30 0.03 88.81

denovo1518 0.00 0.00 0.03 0.30 0.03 88.85

AB084524 0.00 0.00 0.03 0.30 0.03 88.88

FJ269073 0.00 0.00 0.03 0.30 0.03 88.91

denovo2022 0.00 0.00 0.03 0.30 0.03 88.94

AF332511 0.00 0.00 0.03 0.30 0.03 88.97

denovo1764 0.00 0.00 0.03 0.30 0.03 89.00

denovo1940 0.00 0.00 0.03 0.30 0.03 89.04

denovo195 0.00 0.00 0.03 0.30 0.03 89.07

denovo2011 0.00 0.00 0.03 0.30 0.03 89.10

denovo2052 0.00 0.00 0.03 0.30 0.03 89.13

denovo2058 0.00 0.00 0.03 0.30 0.03 89.16

denovo479 0.00 0.00 0.03 0.30 0.03 89.20

denovo522 0.00 0.00 0.03 0.30 0.03 89.23

denovo583 0.00 0.00 0.03 0.30 0.03 89.26

denovo760 0.00 0.00 0.03 0.30 0.03 89.29

denovo85 0.00 0.00 0.03 0.30 0.03 89.32

denovo998 0.00 0.00 0.03 0.30 0.03 89.35

DQ823929 0.00 0.00 0.03 0.30 0.03 89.39

EF020124 0.00 0.00 0.03 0.30 0.03 89.42

EF116592 0.00 0.00 0.03 0.30 0.03 89.45

EU373552 0.00 0.00 0.03 0.30 0.03 89.48

EU982444 0.00 0.00 0.03 0.30 0.03 89.51

FJ557898 0.00 0.00 0.03 0.30 0.03 89.54

FJ666157 0.00 0.00 0.03 0.30 0.03 89.58

FN401326 0.00 0.00 0.03 0.30 0.03 89.61

HQ856409 0.00 0.00 0.03 0.30 0.03 89.64

JQ311869 0.00 0.00 0.03 0.30 0.03 89.67

U87819 0.00 0.00 0.03 0.30 0.03 89.70

denovo486 0.00 0.00 0.03 0.30 0.03 89.73

GQ449231 0.00 0.00 0.03 0.30 0.03 89.77

JN397809 0.00 0.00 0.03 0.30 0.03 89.80

denovo1589 0.00 0.00 0.03 0.42 0.03 89.83

denovo1684 0.00 0.00 0.03 0.41 0.03 89.86

denovo1918 0.00 0.00 0.03 0.41 0.03 89.89

EU768401 0.00 0.00 0.03 0.41 0.03 89.92

FN794273 0.00 0.00 0.03 0.41 0.03 89.95

denovo1203 0.00 0.00 0.03 0.30 0.03 89.98

denovo648 0.00 0.00 0.03 0.30 0.03 90.01

Groups marine & mueller_hinton_dil

Average dissimilarity = 51.02

Group marine Group mueller_hinton_dil

Species Av.Abund Av.Abund Av.Diss Diss/SD Contrib% Cum.%

GQ118701 0.12 0.13 3.82 0.98 7.49 7.49

denovo1749 0.97 0.81 3.68 0.65 7.21 14.70

HE574879 0.02 0.07 1.71 0.81 3.35 18.05

FJ751910 0.00 0.06 1.28 0.29 2.50 20.55

AM709702 0.00 0.05 1.07 0.48 2.10 22.65

denovo1026 0.18 0.03 1.06 0.76 2.08 24.73

JF802167 0.00 0.05 1.04 0.29 2.04 26.76

denovo427 0.02 0.05 1.01 1.02 1.97 28.73

denovo189 0.04 0.03 0.92 1.11 1.80 30.54

denovo1146 0.04 0.03 0.89 1.16 1.75 32.29

denovo1484 0.19 0.02 0.89 0.66 1.75 34.04

denovo1017 0.25 0.01 0.89 0.46 1.74 35.79

denovo75 0.00 0.05 0.88 0.29 1.72 37.50

denovo547 0.01 0.04 0.82 1.00 1.61 39.12

EU846601 0.00 0.04 0.81 0.29 1.58 40.70

denovo552 0.01 0.03 0.80 0.77 1.56 42.26

JN874385 0.01 0.03 0.69 0.66 1.35 43.61

denovo109 0.02 0.02 0.66 1.07 1.29 44.90

denovo1905 0.25 0.00 0.64 0.32 1.25 46.15

denovo507 0.02 0.02 0.63 1.00 1.24 47.39

denovo1189 0.02 0.02 0.61 1.07 1.19 48.58

JF937422 0.18 0.01 0.59 0.43 1.15 49.73

denovo1479 0.00 0.03 0.57 0.66 1.12 50.85

denovo838 0.18 0.00 0.56 0.41 1.11 51.95

denovo1249 0.02 0.02 0.54 1.19 1.07 53.02

denovo119 0.01 0.02 0.52 0.83 1.02 54.03

denovo756 0.18 0.00 0.50 0.36 0.98 55.02

denovo324 0.18 0.00 0.50 0.36 0.98 56.00

denovo114 0.18 0.00 0.50 0.36 0.97 56.97

denovo1743 0.18 0.00 0.49 0.35 0.97 57.93

denovo1476 0.18 0.00 0.48 0.35 0.95 58.88

JN579972 0.00 0.02 0.47 0.42 0.92 59.80

denovo1791 0.18 0.00 0.47 0.33 0.91 60.72

denovo1322 0.18 0.00 0.45 0.32 0.88 61.60

denovo1565 0.18 0.00 0.42 0.30 0.83 62.43

denovo1640 0.18 0.00 0.42 0.30 0.83 63.26

denovo1785 0.18 0.00 0.42 0.30 0.83 64.08

GQ385289 0.18 0.00 0.42 0.30 0.83 64.91

denovo953 0.01 0.02 0.41 0.76 0.81 65.73

denovo828 0.00 0.01 0.36 0.44 0.70 66.42

denovo204 0.01 0.01 0.33 0.76 0.64 67.06

denovo141 0.01 0.01 0.32 1.02 0.63 67.70

denovo1085 0.00 0.01 0.28 0.98 0.55 68.25

JF802166 0.00 0.01 0.28 0.29 0.55 68.81

denovo1070 0.01 0.01 0.27 0.87 0.53 69.34

denovo1462 0.01 0.01 0.27 0.90 0.52 69.87

denovo1292 0.00 0.01 0.26 0.61 0.51 70.38

FJ957855 0.00 0.01 0.25 0.29 0.49 70.86

denovo1145 0.01 0.01 0.24 1.15 0.46 71.32

denovo293 0.00 0.01 0.22 0.34 0.43 71.75

FJ957708 0.00 0.01 0.21 0.29 0.41 72.17

denovo545 0.01 0.01 0.20 1.17 0.40 72.57

denovo415 0.01 0.00 0.20 1.00 0.40 72.96

denovo430 0.01 0.01 0.19 1.08 0.38 73.35

AJ633944 0.00 0.01 0.19 0.53 0.37 73.72

denovo1996 0.00 0.01 0.19 0.29 0.37 74.09

DQ860060 0.00 0.01 0.19 0.66 0.36 74.46

denovo1558 0.00 0.01 0.18 0.29 0.36 74.81

denovo1306 0.00 0.01 0.18 0.29 0.35 75.16

denovo709 0.00 0.01 0.18 0.93 0.35 75.50

AF286480 0.00 0.01 0.18 0.42 0.34 75.85

HQ616267 0.00 0.00 0.18 0.72 0.34 76.19

denovo1193 0.00 0.01 0.17 0.29 0.33 76.52

AY914065 0.00 0.01 0.16 0.57 0.31 76.84

denovo1842 0.01 0.00 0.15 0.80 0.30 77.13

denovo40 0.00 0.01 0.15 0.29 0.29 77.43

denovo1478 0.00 0.01 0.14 0.29 0.28 77.71

denovo198 0.00 0.01 0.13 0.29 0.26 77.97

denovo1390 0.00 0.01 0.13 0.68 0.26 78.23

HQ671075 0.01 0.00 0.13 0.30 0.25 78.48

denovo790 0.00 0.00 0.13 0.76 0.25 78.74

denovo1592 0.00 0.01 0.12 0.29 0.24 78.98

denovo325 0.00 0.01 0.12 0.29 0.24 79.23

X97093 0.00 0.00 0.12 0.51 0.24 79.47

HE574926 0.00 0.01 0.12 0.49 0.23 79.69

FJ169195 0.00 0.00 0.11 0.52 0.22 79.91

denovo1374 0.00 0.00 0.11 0.43 0.21 80.12

EU935300 0.00 0.00 0.11 0.43 0.21 80.33

denovo1579 0.00 0.00 0.10 0.82 0.20 80.53

GQ903461 0.00 0.01 0.10 0.29 0.20 80.73

FJ203135 0.00 0.01 0.10 0.29 0.20 80.93

denovo190 0.00 0.00 0.10 0.76 0.19 81.12

denovo1379 0.00 0.01 0.10 0.55 0.19 81.31

denovo605 0.00 0.00 0.09 0.41 0.18 81.50

denovo1789 0.00 0.00 0.09 0.40 0.18 81.68

denovo1757 0.00 0.00 0.09 0.35 0.17 81.85

denovo854 0.00 0.00 0.09 0.48 0.17 82.02

denovo1230 0.00 0.00 0.09 0.68 0.17 82.19

denovo853 0.00 0.00 0.09 0.61 0.17 82.36

denovo43 0.00 0.00 0.08 0.71 0.17 82.53

denovo792 0.00 0.00 0.08 0.29 0.16 82.69

denovo1983 0.00 0.00 0.08 0.53 0.16 82.84

denovo193 0.00 0.00 0.08 0.54 0.16 83.00

AY631057 0.00 0.00 0.08 0.29 0.15 83.15

denovo1436 0.00 0.00 0.08 0.36 0.15 83.31

EU536078 0.00 0.00 0.08 0.52 0.15 83.46

HE574895 0.00 0.00 0.08 0.29 0.15 83.61

denovo1321 0.00 0.00 0.08 0.42 0.15 83.76

denovo320 0.00 0.00 0.07 0.67 0.15 83.90

denovo1282 0.00 0.00 0.07 0.52 0.14 84.05

denovo1577 0.00 0.00 0.07 0.29 0.14 84.19

FJ826409 0.00 0.00 0.07 0.29 0.14 84.33

denovo1199 0.00 0.00 0.07 0.29 0.14 84.47

denovo1298 0.00 0.00 0.07 0.29 0.14 84.61

denovo1830 0.00 0.00 0.07 0.29 0.14 84.75

denovo1892 0.00 0.00 0.07 0.29 0.14 84.89

denovo481 0.00 0.00 0.07 0.29 0.14 85.03

denovo694 0.00 0.00 0.07 0.29 0.14 85.18

denovo946 0.00 0.00 0.07 0.29 0.14 85.32

DQ833490 0.00 0.00 0.07 0.29 0.14 85.46

HM243844 0.00 0.00 0.07 0.29 0.14 85.60

AB176201 0.00 0.00 0.07 0.53 0.14 85.74

denovo1244 0.00 0.00 0.07 0.29 0.14 85.88

denovo1701 0.00 0.00 0.07 0.68 0.14 86.02

denovo1888 0.00 0.00 0.07 0.53 0.14 86.16

GU584610 0.00 0.00 0.07 0.39 0.14 86.29

denovo7 0.00 0.00 0.07 0.61 0.14 86.43

denovo1088 0.00 0.00 0.07 0.55 0.13 86.56

denovo676 0.00 0.00 0.07 0.51 0.13 86.70

denovo499 0.00 0.00 0.07 0.29 0.13 86.83

denovo611 0.00 0.00 0.07 0.29 0.13 86.96

denovo719 0.00 0.00 0.07 0.48 0.13 87.09

denovo1694 0.00 0.00 0.06 0.50 0.13 87.21

denovo473 0.00 0.00 0.06 0.29 0.12 87.34

denovo1632 0.00 0.00 0.06 0.50 0.12 87.46

denovo1423 0.00 0.00 0.06 0.52 0.12 87.59

denovo1420 0.00 0.00 0.06 0.52 0.12 87.71

denovo1889 0.00 0.00 0.06 0.59 0.12 87.83

denovo613 0.00 0.00 0.06 0.60 0.12 87.95

denovo1459 0.00 0.00 0.06 0.54 0.12 88.07

AF252321 0.00 0.00 0.06 0.29 0.12 88.18

denovo1637 0.00 0.00 0.06 0.50 0.12 88.30

HQ326290 0.00 0.00 0.06 0.29 0.11 88.41

GU584786 0.00 0.00 0.06 0.52 0.11 88.53

denovo1753 0.00 0.00 0.06 0.51 0.11 88.63

denovo1832 0.00 0.00 0.06 0.59 0.11 88.74

denovo1338 0.00 0.00 0.06 0.29 0.11 88.85

denovo272 0.00 0.00 0.05 0.40 0.11 88.96

denovo520 0.00 0.00 0.05 0.29 0.10 89.06

GQ274045 0.00 0.00 0.05 0.41 0.10 89.16

denovo24 0.00 0.00 0.05 0.42 0.10 89.26

denovo692 0.00 0.00 0.05 0.51 0.10 89.36

denovo1403 0.00 0.00 0.05 0.52 0.10 89.46

denovo148 0.00 0.00 0.05 0.29 0.10 89.56

denovo143 0.00 0.00 0.05 0.29 0.10 89.65

denovo1621 0.00 0.00 0.05 0.40 0.09 89.75

denovo862 0.00 0.00 0.05 0.43 0.09 89.84

denovo1962 0.00 0.00 0.05 0.51 0.09 89.94

HM108471 0.00 0.00 0.05 0.52 0.09 90.03

Groups marine_dil & mueller_hinton_dil

Average dissimilarity = 82.91

Group marine_dil Group mueller_hinton_dil

Species Av.Abund Av.Abund Av.Diss Diss/SD Contrib% Cum.%

denovo1749 0.45 0.81 8.07 1.20 9.73 9.73

denovo75 0.15 0.05 3.20 0.49 3.86 13.59

denovo1026 0.17 0.03 2.65 0.92 3.20 16.79

JN579972 0.11 0.02 2.44 0.42 2.94 19.73

FJ624884 0.13 0.00 2.18 0.64 2.63 22.36

GQ118701 0.05 0.13 2.17 0.83 2.61 24.98

JN030555 0.09 0.00 1.90 0.32 2.30 27.27

JF937422 0.13 0.01 1.58 1.34 1.90 29.18

HE574879 0.03 0.07 1.26 0.83 1.52 30.70

FJ751910 0.00 0.06 0.94 0.30 1.13 31.83

AY739689 0.05 0.00 0.92 0.30 1.11 32.94

HQ245988 0.05 0.00 0.84 0.31 1.01 33.96

AM709702 0.01 0.05 0.82 0.51 0.99 34.94

JF802167 0.00 0.05 0.75 0.29 0.90 35.84

AY914065 0.06 0.01 0.74 1.22 0.90 36.74

denovo427 0.01 0.05 0.73 0.90 0.88 37.62

denovo1484 0.05 0.02 0.68 1.15 0.82 38.43

AB299573 0.04 0.00 0.67 0.31 0.81 39.25

HM595366 0.04 0.00 0.65 0.70 0.78 40.02

EU935300 0.05 0.00 0.62 1.28 0.75 40.77

EU846601 0.00 0.04 0.60 0.29 0.72 41.49

denovo1888 0.04 0.00 0.58 0.69 0.70 42.19

U81990 0.04 0.00 0.58 0.46 0.70 42.89

denovo552 0.01 0.03 0.58 0.78 0.70 43.58

GU940713 0.04 0.00 0.56 1.01 0.68 44.26

EU035954 0.04 0.00 0.56 0.76 0.67 44.93

denovo547 0.01 0.04 0.55 0.88 0.67 45.60

denovo1146 0.00 0.03 0.54 0.85 0.66 46.26

denovo1873 0.03 0.00 0.51 0.45 0.62 46.88

denovo189 0.00 0.03 0.50 0.77 0.61 47.49

JN874385 0.01 0.03 0.49 0.68 0.60 48.08

AF286480 0.02 0.01 0.48 0.73 0.57 48.66

HQ118426 0.03 0.00 0.45 0.64 0.55 49.20

denovo1273 0.03 0.00 0.44 1.49 0.54 49.74

DQ889875 0.04 0.00 0.44 0.58 0.53 50.27

AY631057 0.02 0.00 0.42 0.34 0.51 50.77

AM992178 0.02 0.00 0.40 0.30 0.49 51.26

denovo1720 0.02 0.00 0.40 0.73 0.49 51.75

denovo1479 0.00 0.03 0.40 0.63 0.48 52.23

AJ292596 0.02 0.00 0.39 0.57 0.47 52.71

AM935808 0.03 0.00 0.39 0.30 0.47 53.18

denovo109 0.00 0.02 0.38 0.85 0.46 53.63

denovo507 0.01 0.02 0.36 0.83 0.44 54.07

denovo1249 0.00 0.02 0.36 0.96 0.44 54.51

denovo1022 0.03 0.00 0.36 0.82 0.43 54.94

GU118526 0.03 0.00 0.36 0.49 0.43 55.37

denovo119 0.01 0.02 0.36 0.73 0.43 55.80

denovo1189 0.00 0.02 0.35 0.77 0.42 56.22

HQ721418 0.03 0.00 0.34 1.08 0.41 56.63

denovo792 0.02 0.00 0.34 0.49 0.41 57.04

denovo605 0.02 0.00 0.34 0.50 0.41 57.45

AB176201 0.03 0.00 0.33 1.04 0.40 57.85

FQ659744 0.01 0.00 0.32 0.30 0.39 58.24

denovo1848 0.02 0.00 0.32 0.30 0.38 58.62

X97093 0.02 0.00 0.28 0.93 0.34 58.96

denovo953 0.00 0.02 0.28 0.73 0.34 59.30

GU179554 0.02 0.00 0.28 0.91 0.34 59.64

FJ516885 0.01 0.00 0.28 0.39 0.34 59.97

denovo1547 0.02 0.00 0.27 0.54 0.33 60.31

AB477015 0.02 0.00 0.27 1.06 0.33 60.64

denovo1162 0.02 0.00 0.24 0.50 0.29 60.93

denovo2019 0.01 0.00 0.24 0.61 0.29 61.22

denovo828 0.00 0.01 0.24 0.42 0.29 61.50

denovo1842 0.01 0.00 0.24 0.85 0.29 61.79

AF252321 0.01 0.00 0.24 0.35 0.28 62.08

denovo1017 0.00 0.01 0.24 0.62 0.28 62.36

denovo545 0.00 0.01 0.22 1.23 0.27 62.63

denovo1923 0.01 0.00 0.22 0.63 0.27 62.89

denovo550 0.01 0.00 0.22 0.30 0.26 63.15

denovo204 0.01 0.01 0.22 0.76 0.26 63.42

JN868782 0.02 0.00 0.22 1.01 0.26 63.68

AJ347026 0.02 0.00 0.21 0.54 0.25 63.93

HQ616267 0.01 0.00 0.21 0.97 0.25 64.18

denovo1190 0.01 0.00 0.20 0.62 0.25 64.43

JF802166 0.00 0.01 0.20 0.29 0.24 64.67

EU160577 0.02 0.00 0.20 0.73 0.24 64.92

denovo862 0.01 0.00 0.20 0.45 0.24 65.16

FJ418924 0.02 0.00 0.20 0.54 0.24 65.39

denovo1306 0.00 0.01 0.20 0.44 0.24 65.63

denovo1070 0.01 0.01 0.20 0.87 0.24 65.86

denovo1085 0.00 0.01 0.19 0.89 0.24 66.10

denovo1263 0.01 0.00 0.19 0.30 0.23 66.33

denovo1182 0.02 0.00 0.19 0.58 0.23 66.56

denovo854 0.01 0.00 0.18 0.59 0.22 66.78

FJ957855 0.00 0.01 0.18 0.29 0.22 67.00

denovo1292 0.00 0.01 0.18 0.55 0.21 67.21

denovo822 0.01 0.00 0.18 0.50 0.21 67.43

denovo776 0.02 0.00 0.18 0.53 0.21 67.64

denovo1632 0.01 0.00 0.18 0.79 0.21 67.85

EU536078 0.01 0.00 0.18 0.67 0.21 68.06

denovo1577 0.01 0.00 0.17 0.70 0.21 68.27

denovo607 0.01 0.00 0.17 0.52 0.21 68.48

denovo141 0.00 0.01 0.16 0.66 0.19 68.67

AF170746 0.02 0.00 0.16 0.49 0.19 68.86

FJ957708 0.00 0.01 0.15 0.29 0.18 69.05

denovo1462 0.00 0.01 0.15 0.67 0.18 69.23

denovo1250 0.01 0.00 0.15 0.74 0.18 69.41

DQ860060 0.00 0.01 0.15 0.70 0.18 69.59

denovo40 0.00 0.01 0.15 0.41 0.18 69.76

denovo1996 0.00 0.01 0.14 0.29 0.17 69.93

GU118575 0.01 0.00 0.14 0.53 0.17 70.10

denovo1145 0.00 0.01 0.14 0.74 0.17 70.27

denovo1558 0.00 0.01 0.14 0.29 0.17 70.43

AJ633944 0.00 0.01 0.14 0.52 0.16 70.60

GU553034 0.01 0.00 0.14 0.34 0.16 70.76

HM108471 0.01 0.00 0.14 0.63 0.16 70.93

denovo1964 0.01 0.00 0.13 0.56 0.16 71.08

denovo61 0.01 0.00 0.13 0.64 0.15 71.24

EU803928 0.01 0.00 0.13 0.52 0.15 71.39

denovo98 0.01 0.00 0.13 0.54 0.15 71.54

denovo1284 0.01 0.00 0.13 0.55 0.15 71.70

JF937433 0.01 0.00 0.12 0.57 0.15 71.84

FJ892785 0.01 0.00 0.12 0.68 0.15 71.99

denovo198 0.00 0.01 0.12 0.36 0.15 72.14

GU118690 0.01 0.00 0.12 0.56 0.15 72.29

denovo1193 0.00 0.01 0.12 0.29 0.15 72.44

GQ263306 0.01 0.00 0.12 0.69 0.15 72.58

denovo293 0.00 0.01 0.12 0.29 0.15 72.73

GQ921403 0.01 0.00 0.12 0.30 0.14 72.87

denovo696 0.01 0.00 0.12 0.30 0.14 73.01

denovo663 0.01 0.00 0.12 0.55 0.14 73.15

denovo1420 0.01 0.00 0.12 0.66 0.14 73.29

DQ316817 0.01 0.00 0.12 0.30 0.14 73.43

denovo574 0.01 0.00 0.12 0.47 0.14 73.57

denovo430 0.00 0.01 0.11 0.74 0.14 73.71

denovo249 0.01 0.00 0.11 0.80 0.13 73.84

GQ903461 0.00 0.01 0.11 0.40 0.13 73.98

DQ889881 0.01 0.00 0.11 0.57 0.13 74.10

denovo1478 0.00 0.01 0.11 0.29 0.13 74.23

denovo499 0.00 0.00 0.11 0.41 0.13 74.36

denovo709 0.00 0.01 0.10 0.73 0.12 74.49

GU118606 0.01 0.00 0.10 0.57 0.12 74.61

FJ900573 0.01 0.00 0.10 0.57 0.12 74.73

EU463921 0.01 0.00 0.10 0.44 0.12 74.85

FJ203135 0.00 0.01 0.10 0.37 0.12 74.96

FJ557790 0.01 0.00 0.09 0.51 0.11 75.08

denovo1961 0.01 0.00 0.09 0.48 0.11 75.19

JF925031 0.01 0.00 0.09 0.69 0.11 75.30

GU305772 0.01 0.00 0.09 0.67 0.11 75.42

denovo1592 0.00 0.01 0.09 0.29 0.11 75.53

denovo325 0.00 0.01 0.09 0.29 0.11 75.64

FJ820465 0.01 0.00 0.09 0.44 0.11 75.75

denovo1757 0.00 0.00 0.09 0.49 0.11 75.86

FJ826409 0.00 0.00 0.09 0.47 0.11 75.97

denovo423 0.01 0.00 0.09 0.44 0.11 76.08

denovo917 0.00 0.00 0.09 0.34 0.11 76.18

denovo412 0.00 0.00 0.09 0.49 0.11 76.29

FJ802385 0.01 0.00 0.09 0.56 0.11 76.39

denovo528 0.01 0.00 0.09 0.34 0.10 76.50

denovo305 0.01 0.00 0.09 0.54 0.10 76.60

EF076171 0.01 0.00 0.08 0.63 0.10 76.70

denovo788 0.01 0.00 0.08 0.30 0.10 76.81

denovo415 0.00 0.00 0.08 0.80 0.10 76.91

denovo1480 0.00 0.00 0.08 0.39 0.10 77.01

AY959053 0.01 0.00 0.08 0.57 0.10 77.10

FJ381979 0.01 0.00 0.08 0.56 0.10 77.20

denovo1374 0.00 0.00 0.08 0.46 0.10 77.29

denovo756 0.00 0.00 0.08 0.61 0.09 77.39

denovo1390 0.00 0.01 0.08 0.60 0.09 77.48

HE574926 0.00 0.01 0.08 0.45 0.09 77.57

FJ999591 0.01 0.00 0.08 0.50 0.09 77.66

denovo114 0.00 0.00 0.08 0.61 0.09 77.76

EF018153 0.00 0.00 0.07 0.30 0.09 77.84

denovo744 0.00 0.00 0.07 0.48 0.09 77.93

denovo1590 0.01 0.00 0.07 0.55 0.09 78.02

denovo1379 0.00 0.01 0.07 0.56 0.09 78.11

denovo1898 0.00 0.00 0.07 0.49 0.09 78.19

HQ753432 0.01 0.00 0.07 0.53 0.09 78.28

denovo2003 0.01 0.00 0.07 0.44 0.09 78.37

denovo88 0.00 0.00 0.07 0.30 0.08 78.45

denovo790 0.00 0.00 0.07 0.54 0.08 78.53

FJ457296 0.00 0.00 0.07 0.30 0.08 78.61

denovo165 0.01 0.00 0.07 0.39 0.08 78.69

denovo1526 0.00 0.00 0.07 0.30 0.08 78.77

denovo308 0.00 0.00 0.07 0.56 0.08 78.85

denovo1705 0.00 0.00 0.07 0.30 0.08 78.93

denovo1414 0.00 0.00 0.06 0.30 0.08 79.01

denovo1753 0.00 0.00 0.06 0.45 0.08 79.08

denovo838 0.00 0.00 0.06 0.59 0.08 79.16

denovo1696 0.00 0.00 0.06 0.30 0.08 79.24

EF076172 0.01 0.00 0.06 0.54 0.07 79.31

FJ529262 0.01 0.00 0.06 0.52 0.07 79.39

denovo1585 0.00 0.00 0.06 0.43 0.07 79.46

denovo1093 0.00 0.00 0.06 0.30 0.07 79.53

HQ326290 0.00 0.00 0.06 0.40 0.07 79.61

denovo860 0.00 0.00 0.06 0.30 0.07 79.68

denovo659 0.00 0.00 0.06 0.53 0.07 79.75

denovo1703 0.00 0.00 0.06 0.43 0.07 79.82

denovo916 0.00 0.00 0.06 0.43 0.07 79.89

denovo1983 0.00 0.00 0.06 0.53 0.07 79.97

denovo103 0.00 0.00 0.06 0.30 0.07 80.04

AJ621576 0.01 0.00 0.06 0.55 0.07 80.11

denovo707 0.01 0.00 0.06 0.43 0.07 80.18

denovo1670 0.00 0.00 0.06 0.42 0.07 80.25

denovo193 0.00 0.00 0.06 0.54 0.07 80.32

HE574895 0.00 0.00 0.06 0.29 0.07 80.39

denovo1303 0.01 0.00 0.06 0.40 0.07 80.46

denovo1688 0.00 0.00 0.06 0.30 0.07 80.53

denovo324 0.00 0.00 0.06 0.55 0.07 80.60

HQ118340 0.00 0.00 0.06 0.53 0.07 80.67

denovo1555 0.00 0.00 0.06 0.40 0.07 80.73

denovo1321 0.00 0.00 0.06 0.43 0.07 80.80

HQ672216 0.00 0.00 0.06 0.42 0.07 80.87

FQ660217 0.00 0.00 0.06 0.44 0.07 80.94

EU817491 0.00 0.00 0.06 0.42 0.07 81.01

AJ583166 0.00 0.00 0.05 0.36 0.07 81.07

denovo521 0.00 0.00 0.05 0.30 0.07 81.14

denovo1282 0.00 0.00 0.05 0.52 0.07 81.20

denovo1199 0.00 0.00 0.05 0.29 0.06 81.27

denovo1298 0.00 0.00 0.05 0.29 0.06 81.33

denovo1830 0.00 0.00 0.05 0.29 0.06 81.39

denovo1892 0.00 0.00 0.05 0.29 0.06 81.46

denovo481 0.00 0.00 0.05 0.29 0.06 81.52

denovo694 0.00 0.00 0.05 0.29 0.06 81.59

denovo946 0.00 0.00 0.05 0.29 0.06 81.65

DQ833490 0.00 0.00 0.05 0.29 0.06 81.72

HM243844 0.00 0.00 0.05 0.29 0.06 81.78

denovo190 0.00 0.00 0.05 0.66 0.06 81.84

denovo1423 0.00 0.00 0.05 0.52 0.06 81.91

GU584610 0.00 0.00 0.05 0.40 0.06 81.97

DQ889935 0.00 0.00 0.05 0.44 0.06 82.03

JN411267 0.00 0.00 0.05 0.43 0.06 82.10

denovo1641 0.00 0.00 0.05 0.30 0.06 82.16

denovo1147 0.00 0.00 0.05 0.37 0.06 82.22

denovo767 0.00 0.00 0.05 0.30 0.06 82.28

denovo1341 0.00 0.00 0.05 0.30 0.06 82.34

denovo1082 0.00 0.00 0.05 0.30 0.06 82.40

denovo1556 0.00 0.00 0.05 0.30 0.06 82.46

denovo611 0.00 0.00 0.05 0.29 0.06 82.52

denovo1244 0.00 0.00 0.05 0.29 0.06 82.57

denovo1875 0.00 0.00 0.05 0.30 0.06 82.63

denovo1436 0.00 0.00 0.05 0.29 0.06 82.69

AJ863291 0.00 0.00 0.05 0.30 0.06 82.74

denovo1274 0.00 0.00 0.05 0.30 0.06 82.80

denovo1265 0.00 0.00 0.05 0.44 0.06 82.86

denovo898 0.00 0.00 0.05 0.44 0.06 82.91

denovo473 0.00 0.00 0.05 0.29 0.05 82.97

denovo1240 0.00 0.00 0.04 0.42 0.05 83.02

denovo1095 0.00 0.00 0.04 0.30 0.05 83.07

denovo7 0.00 0.00 0.04 0.54 0.05 83.13

denovo1459 0.00 0.00 0.04 0.55 0.05 83.18

denovo818 0.00 0.00 0.04 0.30 0.05 83.23

GU563744 0.00 0.00 0.04 0.43 0.05 83.28

denovo520 0.00 0.00 0.04 0.44 0.05 83.33

denovo543 0.00 0.00 0.04 0.41 0.05 83.38

denovo657 0.00 0.00 0.04 0.30 0.05 83.44

denovo1383 0.00 0.00 0.04 0.30 0.05 83.49

denovo225 0.00 0.00 0.04 0.30 0.05 83.54

EF153415 0.00 0.00 0.04 0.42 0.05 83.59

denovo1415 0.00 0.00 0.04 0.41 0.05 83.64

denovo727 0.00 0.00 0.04 0.44 0.05 83.69

denovo624 0.00 0.00 0.04 0.30 0.05 83.74

denovo28 0.00 0.00 0.04 0.30 0.05 83.79

denovo1299 0.00 0.00 0.04 0.30 0.05 83.84

denovo787 0.00 0.00 0.04 0.30 0.05 83.88

denovo918 0.00 0.00 0.04 0.30 0.05 83.93

FJ946588 0.00 0.00 0.04 0.30 0.05 83.98

FJ497578 0.00 0.00 0.04 0.44 0.05 84.03

FJ675001 0.01 0.00 0.04 0.30 0.05 84.08

denovo358 0.00 0.00 0.04 0.30 0.05 84.13

EU794092 0.00 0.00 0.04 0.43 0.05 84.18

denovo272 0.00 0.00 0.04 0.40 0.05 84.23

denovo1226 0.00 0.00 0.04 0.30 0.05 84.27

denovo853 0.00 0.00 0.04 0.55 0.05 84.32

denovo244 0.00 0.00 0.04 0.41 0.05 84.36

denovo819 0.00 0.00 0.04 0.30 0.05 84.41

FN666870 0.00 0.00 0.04 0.30 0.05 84.46

denovo769 0.00 0.00 0.04 0.44 0.05 84.50

denovo1743 0.00 0.00 0.04 0.40 0.05 84.55

denovo431 0.00 0.00 0.04 0.42 0.04 84.59

denovo24 0.00 0.00 0.04 0.42 0.04 84.64

JF808978 0.00 0.00 0.04 0.44 0.04 84.68

HQ143274 0.00 0.00 0.04 0.30 0.04 84.73

denovo1855 0.00 0.00 0.04 0.30 0.04 84.77

denovo66 0.00 0.00 0.04 0.30 0.04 84.81

denovo676 0.00 0.00 0.04 0.42 0.04 84.86

denovo1561 0.00 0.00 0.04 0.30 0.04 84.90

JF712668 0.00 0.00 0.04 0.52 0.04 84.94

denovo1243 0.00 0.00 0.04 0.30 0.04 84.99

denovo1412 0.00 0.00 0.04 0.30 0.04 85.03

denovo1014 0.00 0.00 0.04 0.30 0.04 85.07

denovo993 0.00 0.00 0.04 0.30 0.04 85.11

denovo1139 0.00 0.00 0.04 0.30 0.04 85.16

denovo1443 0.00 0.00 0.04 0.30 0.04 85.20

denovo1911 0.00 0.00 0.04 0.30 0.04 85.24

denovo1937 0.00 0.00 0.04 0.30 0.04 85.28

denovo1998 0.00 0.00 0.04 0.30 0.04 85.33

denovo565 0.00 0.00 0.04 0.30 0.04 85.37

denovo921 0.00 0.00 0.04 0.30 0.04 85.41

denovo143 0.00 0.00 0.04 0.29 0.04 85.45

denovo797 0.00 0.00 0.04 0.39 0.04 85.50

denovo6 0.00 0.00 0.04 0.30 0.04 85.54

FJ808721 0.00 0.00 0.03 0.43 0.04 85.58

denovo148 0.00 0.00 0.03 0.29 0.04 85.62

denovo1237 0.00 0.00 0.03 0.30 0.04 85.66

denovo1856 0.00 0.00 0.03 0.30 0.04 85.70

denovo236 0.00 0.00 0.03 0.30 0.04 85.75

denovo94 0.00 0.00 0.03 0.30 0.04 85.79

denovo104 0.00 0.00 0.03 0.43 0.04 85.83

denovo631 0.00 0.00 0.03 0.30 0.04 85.87

denovo1881 0.00 0.00 0.03 0.41 0.04 85.91

denovo1523 0.00 0.00 0.03 0.29 0.04 85.95

JN544140 0.00 0.00 0.03 0.44 0.04 85.99

denovo1775 0.00 0.00 0.03 0.30 0.04 86.03

denovo260 0.00 0.00 0.03 0.30 0.04 86.07

GU584786 0.00 0.00 0.03 0.43 0.04 86.11

denovo804 0.00 0.00 0.03 0.41 0.04 86.15

denovo432 0.00 0.00 0.03 0.41 0.04 86.19

denovo1694 0.00 0.00 0.03 0.41 0.04 86.23

HQ616309 0.00 0.00 0.03 0.30 0.04 86.27

denovo1637 0.00 0.00 0.03 0.42 0.04 86.31

DQ129268 0.00 0.00 0.03 0.42 0.04 86.35

denovo708 0.00 0.00 0.03 0.29 0.04 86.38

denovo1944 0.00 0.00 0.03 0.41 0.04 86.42

denovo492 0.00 0.00 0.03 0.41 0.04 86.46

denovo1740 0.00 0.00 0.03 0.41 0.04 86.50

denovo1930 0.00 0.00 0.03 0.41 0.04 86.53

GU118588 0.00 0.00 0.03 0.30 0.04 86.57

EU776368 0.00 0.00 0.03 0.30 0.04 86.61

denovo1457 0.00 0.00 0.03 0.44 0.04 86.65

denovo1391 0.00 0.00 0.03 0.44 0.04 86.68

denovo827 0.00 0.00 0.03 0.30 0.04 86.72

denovo2018 0.00 0.00 0.03 0.30 0.04 86.76

denovo1396 0.00 0.00 0.03 0.30 0.04 86.79

denovo1839 0.00 0.00 0.03 0.29 0.04 86.83

denovo2047 0.00 0.00 0.03 0.30 0.04 86.86

denovo1899 0.00 0.00 0.03 0.29 0.04 86.90

denovo1230 0.00 0.00 0.03 0.41 0.04 86.93

denovo660 0.00 0.00 0.03 0.41 0.04 86.97

denovo1936 0.00 0.00 0.03 0.30 0.04 87.00

denovo600 0.00 0.00 0.03 0.30 0.03 87.04

denovo1845 0.00 0.00 0.03 0.30 0.03 87.07

denovo977 0.00 0.00 0.03 0.30 0.03 87.11

denovo1643 0.00 0.00 0.03 0.30 0.03 87.14

denovo593 0.00 0.00 0.03 0.30 0.03 87.18

denovo1110 0.00 0.00 0.03 0.30 0.03 87.21

denovo1141 0.00 0.00 0.03 0.30 0.03 87.25

denovo1227 0.00 0.00 0.03 0.30 0.03 87.28

denovo1254 0.00 0.00 0.03 0.30 0.03 87.32

denovo1264 0.00 0.00 0.03 0.30 0.03 87.35

denovo1309 0.00 0.00 0.03 0.30 0.03 87.39

denovo1340 0.00 0.00 0.03 0.30 0.03 87.42

denovo1407 0.00 0.00 0.03 0.30 0.03 87.46

denovo142 0.00 0.00 0.03 0.30 0.03 87.49

denovo1521 0.00 0.00 0.03 0.30 0.03 87.52

denovo1674 0.00 0.00 0.03 0.30 0.03 87.56

denovo1727 0.00 0.00 0.03 0.30 0.03 87.59

denovo1751 0.00 0.00 0.03 0.30 0.03 87.63

denovo1877 0.00 0.00 0.03 0.30 0.03 87.66

denovo1999 0.00 0.00 0.03 0.30 0.03 87.70

denovo25 0.00 0.00 0.03 0.30 0.03 87.73

denovo468 0.00 0.00 0.03 0.30 0.03 87.77

denovo603 0.00 0.00 0.03 0.30 0.03 87.80

denovo618 0.00 0.00 0.03 0.30 0.03 87.84

denovo717 0.00 0.00 0.03 0.30 0.03 87.87

GU941113 0.00 0.00 0.03 0.30 0.03 87.91

denovo385 0.00 0.00 0.03 0.30 0.03 87.94

denovo728 0.00 0.00 0.03 0.30 0.03 87.97

denovo692 0.00 0.00 0.03 0.42 0.03 88.01

HQ757459 0.00 0.00 0.03 0.30 0.03 88.04

denovo1824 0.00 0.00 0.03 0.30 0.03 88.08

EU794074 0.00 0.00 0.03 0.30 0.03 88.11

denovo1653 0.00 0.00 0.03 0.43 0.03 88.14

denovo32 0.00 0.00 0.03 0.43 0.03 88.18

FJ957596 0.00 0.00 0.03 0.44 0.03 88.21

denovo846 0.00 0.00 0.03 0.29 0.03 88.24

denovo1385 0.00 0.00 0.03 0.30 0.03 88.28

denovo161 0.00 0.00 0.03 0.30 0.03 88.31

denovo332 0.00 0.00 0.03 0.30 0.03 88.34

denovo428 0.00 0.00 0.03 0.30 0.03 88.37

denovo535 0.00 0.00 0.03 0.30 0.03 88.41

denovo1476 0.00 0.00 0.03 0.43 0.03 88.44

denovo1962 0.00 0.00 0.03 0.43 0.03 88.47

denovo613 0.00 0.00 0.03 0.43 0.03 88.50

denovo1401 0.00 0.00 0.03 0.43 0.03 88.53

denovo1467 0.00 0.00 0.03 0.43 0.03 88.57

EU844911 0.00 0.00 0.03 0.43 0.03 88.60

JL812080 0.00 0.00 0.03 0.43 0.03 88.63

denovo486 0.00 0.00 0.03 0.30 0.03 88.66

GQ449231 0.00 0.00 0.03 0.30 0.03 88.69

JN397809 0.00 0.00 0.03 0.30 0.03 88.72

denovo1713 0.00 0.00 0.03 0.42 0.03 88.75

denovo850 0.00 0.00 0.03 0.42 0.03 88.79

denovo1633 0.00 0.00 0.03 0.30 0.03 88.82

HQ323455 0.00 0.00 0.03 0.30 0.03 88.85

denovo1584 0.00 0.00 0.03 0.30 0.03 88.88

denovo1518 0.00 0.00 0.03 0.30 0.03 88.91

AB084524 0.00 0.00 0.03 0.30 0.03 88.94

denovo1491 0.00 0.00 0.03 0.30 0.03 88.97

FJ269073 0.00 0.00 0.03 0.30 0.03 89.00

denovo2022 0.00 0.00 0.03 0.30 0.03 89.03

AF332511 0.00 0.00 0.03 0.30 0.03 89.06

denovo1764 0.00 0.00 0.03 0.30 0.03 89.09

denovo1940 0.00 0.00 0.03 0.30 0.03 89.13

denovo195 0.00 0.00 0.03 0.30 0.03 89.16

denovo2011 0.00 0.00 0.03 0.30 0.03 89.19

denovo2052 0.00 0.00 0.03 0.30 0.03 89.22

denovo2058 0.00 0.00 0.03 0.30 0.03 89.25

denovo479 0.00 0.00 0.03 0.30 0.03 89.28

denovo522 0.00 0.00 0.03 0.30 0.03 89.31

denovo583 0.00 0.00 0.03 0.30 0.03 89.34

denovo760 0.00 0.00 0.03 0.30 0.03 89.37

denovo85 0.00 0.00 0.03 0.30 0.03 89.40

denovo998 0.00 0.00 0.03 0.30 0.03 89.43

DQ823929 0.00 0.00 0.03 0.30 0.03 89.47

EF020124 0.00 0.00 0.03 0.30 0.03 89.50

EF116592 0.00 0.00 0.03 0.30 0.03 89.53

EU373552 0.00 0.00 0.03 0.30 0.03 89.56

EU982444 0.00 0.00 0.03 0.30 0.03 89.59

FJ557898 0.00 0.00 0.03 0.30 0.03 89.62

FJ666157 0.00 0.00 0.03 0.30 0.03 89.65

FN401326 0.00 0.00 0.03 0.30 0.03 89.68

HQ856409 0.00 0.00 0.03 0.30 0.03 89.71

JQ311869 0.00 0.00 0.03 0.30 0.03 89.74

U87819 0.00 0.00 0.03 0.30 0.03 89.77

denovo158 0.00 0.00 0.03 0.41 0.03 89.81

denovo1827 0.00 0.00 0.03 0.43 0.03 89.84

denovo194 0.00 0.00 0.03 0.43 0.03 89.87

denovo1403 0.00 0.00 0.02 0.43 0.03 89.90

denovo571 0.00 0.00 0.02 0.29 0.03 89.93

FJ214667 0.00 0.00 0.02 0.29 0.03 89.96

denovo1621 0.00 0.00 0.02 0.29 0.03 89.99

denovo1801 0.00 0.00 0.02 0.42 0.03 90.02

Groups mueller_hinton & mueller_hinton_dil

Average dissimilarity = 69.46

Group mueller_hinton Group mueller_hinton_dil

Species Av.Abund Av.Abund Av.Diss Diss/SD Contrib% Cum.%

denovo1749 0.60 0.81 8.99 0.97 12.94 12.94

GQ118701 0.03 0.13 3.09 0.81 4.45 17.39

JN579972 0.08 0.02 2.41 0.37 3.47 20.86

FJ516885 0.08 0.00 2.37 0.30 3.41 24.27

HE574879 0.07 0.07 2.24 0.81 3.23 27.50

HM595366 0.13 0.00 2.24 0.58 3.23 30.73

denovo1026 0.12 0.03 2.19 0.87 3.15 33.89

denovo1888 0.12 0.00 2.04 0.59 2.94 36.82

FJ751910 0.00 0.06 1.26 0.30 1.81 38.63

U81990 0.07 0.00 1.25 0.57 1.79 40.42

JF802167 0.01 0.05 1.15 0.34 1.65 42.07

denovo552 0.03 0.03 1.01 0.78 1.46 43.53

denovo427 0.01 0.05 1.01 0.95 1.45 44.99

AM709702 0.00 0.05 1.00 0.46 1.44 46.43

denovo75 0.00 0.05 0.87 0.30 1.25 47.68

denovo1484 0.04 0.02 0.87 0.98 1.25 48.92

EU846601 0.00 0.04 0.80 0.30 1.15 50.07

denovo547 0.01 0.04 0.79 0.93 1.14 51.22

denovo605 0.04 0.00 0.79 0.48 1.14 52.35

denovo1146 0.00 0.03 0.76 0.93 1.10 53.45

denovo189 0.00 0.03 0.72 0.84 1.03 54.48

JN874385 0.01 0.03 0.67 0.64 0.97 55.45

denovo862 0.03 0.00 0.61 0.48 0.88 56.33

denovo1479 0.00 0.03 0.54 0.66 0.78 57.11

JF937422 0.03 0.01 0.54 1.27 0.78 57.89

denovo109 0.00 0.02 0.53 0.85 0.77 58.66

denovo1961 0.03 0.00 0.51 0.58 0.73 59.39

denovo119 0.01 0.02 0.50 0.76 0.72 60.11

denovo507 0.00 0.02 0.50 0.78 0.72 60.83

denovo1249 0.00 0.02 0.50 1.05 0.72 61.54

denovo1189 0.00 0.02 0.50 0.84 0.71 62.26

denovo1243 0.03 0.00 0.48 0.55 0.69 62.94

denovo953 0.00 0.02 0.39 0.69 0.56 63.50

AB176201 0.02 0.00 0.39 0.64 0.56 64.06

denovo1022 0.02 0.00 0.31 0.42 0.44 64.50

denovo828 0.00 0.01 0.30 0.37 0.43 64.93

EU536078 0.01 0.00 0.28 0.61 0.40 65.33

JF802166 0.00 0.01 0.28 0.30 0.40 65.73

AF286480 0.01 0.01 0.27 0.57 0.40 66.13

denovo1085 0.00 0.01 0.27 0.93 0.39 66.52

denovo1017 0.00 0.01 0.27 0.60 0.39 66.91

denovo545 0.01 0.01 0.27 1.26 0.38 67.29

HQ323455 0.01 0.00 0.26 0.30 0.37 67.67

denovo1292 0.00 0.01 0.25 0.60 0.36 68.03

denovo854 0.01 0.00 0.25 0.49 0.36 68.38

denovo114 0.01 0.00 0.25 0.53 0.36 68.74

denovo1129 0.01 0.00 0.24 0.57 0.35 69.09

FJ957855 0.00 0.01 0.24 0.30 0.35 69.44

denovo1070 0.01 0.01 0.24 0.76 0.35 69.79

denovo141 0.00 0.01 0.23 0.67 0.33 70.12

denovo1703 0.01 0.00 0.23 0.50 0.33 70.45

denovo1462 0.00 0.01 0.22 0.74 0.32 70.77

AY914065 0.01 0.01 0.22 0.88 0.32 71.09

denovo1412 0.01 0.00 0.22 0.43 0.31 71.40

denovo204 0.00 0.01 0.21 0.55 0.31 71.71

denovo1753 0.01 0.00 0.21 0.35 0.31 72.02

denovo1558 0.00 0.01 0.21 0.35 0.30 72.32

denovo1374 0.01 0.00 0.21 0.44 0.30 72.62

FJ957708 0.00 0.01 0.21 0.30 0.30 72.92

denovo1273 0.01 0.00 0.21 0.47 0.30 73.22

denovo1145 0.00 0.01 0.20 0.76 0.29 73.50

denovo927 0.01 0.00 0.20 0.43 0.28 73.79

denovo1996 0.00 0.01 0.19 0.30 0.27 74.06

denovo293 0.00 0.01 0.18 0.29 0.26 74.32

denovo1491 0.01 0.00 0.18 0.30 0.26 74.59

FJ849072 0.01 0.00 0.18 0.30 0.26 74.85

denovo415 0.01 0.00 0.18 0.98 0.26 75.11

denovo1096 0.01 0.00 0.18 0.42 0.25 75.36

denovo1306 0.00 0.01 0.17 0.30 0.25 75.61

DQ860060 0.00 0.01 0.17 0.62 0.25 75.86

denovo95 0.01 0.00 0.17 0.36 0.24 76.11

denovo1193 0.00 0.01 0.17 0.30 0.24 76.35

AJ633944 0.00 0.01 0.16 0.47 0.23 76.58

denovo430 0.00 0.01 0.16 0.75 0.23 76.82

denovo709 0.00 0.01 0.16 0.79 0.23 77.05

EU935300 0.00 0.00 0.16 0.66 0.23 77.28

denovo1873 0.00 0.00 0.15 0.48 0.22 77.49

HM108471 0.01 0.00 0.15 0.55 0.21 77.71

AF170746 0.01 0.00 0.15 0.34 0.21 77.92

denovo40 0.00 0.01 0.15 0.30 0.21 78.13

JF937433 0.01 0.00 0.15 0.47 0.21 78.34

denovo1478 0.00 0.01 0.14 0.30 0.21 78.55

denovo767 0.01 0.00 0.14 0.44 0.20 78.75

HE574926 0.00 0.01 0.13 0.55 0.19 78.95

denovo1446 0.01 0.00 0.13 0.43 0.19 79.14

denovo198 0.00 0.01 0.13 0.30 0.19 79.33

denovo1921 0.01 0.00 0.13 0.33 0.19 79.52

GU940713 0.01 0.00 0.13 0.30 0.19 79.70

denovo916 0.01 0.00 0.13 0.30 0.19 79.89

denovo1570 0.01 0.00 0.13 0.30 0.19 80.08

denovo2056 0.01 0.00 0.13 0.30 0.19 80.26

denovo757 0.01 0.00 0.13 0.30 0.19 80.45

JQ032050 0.01 0.00 0.13 0.30 0.19 80.64

denovo1577 0.00 0.00 0.13 0.42 0.18 80.82

GQ903461 0.00 0.01 0.13 0.37 0.18 81.00

denovo2029 0.01 0.00 0.13 0.42 0.18 81.18

denovo1341 0.01 0.00 0.12 0.42 0.18 81.36

denovo1592 0.00 0.01 0.12 0.30 0.18 81.54

denovo325 0.00 0.01 0.12 0.30 0.18 81.72

HQ616267 0.00 0.00 0.12 0.48 0.17 81.89

denovo1390 0.00 0.01 0.12 0.69 0.17 82.06

denovo2003 0.01 0.00 0.12 0.43 0.17 82.23

FJ203135 0.00 0.01 0.11 0.34 0.16 82.39

denovo838 0.00 0.00 0.11 0.83 0.16 82.55

AB294294 0.00 0.00 0.11 0.37 0.15 82.70

denovo6 0.00 0.00 0.10 0.29 0.15 82.85

denovo790 0.00 0.00 0.10 0.62 0.15 83.00

denovo706 0.00 0.00 0.10 0.35 0.14 83.14

denovo1379 0.00 0.01 0.10 0.56 0.14 83.28

denovo792 0.00 0.00 0.10 0.35 0.14 83.41

denovo190 0.00 0.00 0.09 0.71 0.13 83.55

GQ433928 0.00 0.00 0.09 0.29 0.13 83.68

denovo1757 0.00 0.00 0.09 0.36 0.12 83.81

denovo1842 0.00 0.00 0.08 0.63 0.12 83.93

HQ326290 0.00 0.00 0.08 0.40 0.12 84.05

denovo679 0.00 0.00 0.08 0.36 0.12 84.17

denovo639 0.00 0.00 0.08 0.30 0.12 84.29

FN396956 0.00 0.00 0.08 0.30 0.12 84.41

denovo1500 0.00 0.00 0.08 0.30 0.12 84.52

denovo1983 0.00 0.00 0.08 0.54 0.11 84.64

denovo193 0.00 0.00 0.08 0.55 0.11 84.75

AY631057 0.00 0.00 0.08 0.30 0.11 84.86

denovo324 0.00 0.00 0.08 0.56 0.11 84.97

denovo1436 0.00 0.00 0.08 0.37 0.11 85.08

HE574895 0.00 0.00 0.08 0.30 0.11 85.19

denovo1619 0.00 0.00 0.08 0.42 0.11 85.30

denovo1420 0.00 0.00 0.08 0.62 0.11 85.41

denovo1321 0.00 0.00 0.07 0.44 0.11 85.52

denovo1282 0.00 0.00 0.07 0.53 0.10 85.62

X97093 0.00 0.00 0.07 0.30 0.10 85.72

FJ826409 0.00 0.00 0.07 0.30 0.10 85.83

denovo1199 0.00 0.00 0.07 0.30 0.10 85.93

denovo1298 0.00 0.00 0.07 0.30 0.10 86.03

denovo1830 0.00 0.00 0.07 0.30 0.10 86.14

denovo1892 0.00 0.00 0.07 0.30 0.10 86.24

denovo481 0.00 0.00 0.07 0.30 0.10 86.34

denovo694 0.00 0.00 0.07 0.30 0.10 86.44

denovo946 0.00 0.00 0.07 0.30 0.10 86.55

DQ833490 0.00 0.00 0.07 0.30 0.10 86.65

HM243844 0.00 0.00 0.07 0.30 0.10 86.75

AJ240982 0.00 0.00 0.07 0.29 0.10 86.85

denovo1244 0.00 0.00 0.07 0.29 0.10 86.95

GU584610 0.00 0.00 0.07 0.40 0.10 87.05

denovo7 0.00 0.00 0.07 0.63 0.10 87.15

denovo756 0.00 0.00 0.07 0.52 0.10 87.25

denovo499 0.00 0.00 0.07 0.30 0.10 87.34

denovo611 0.00 0.00 0.07 0.30 0.10 87.44

GU179554 0.00 0.00 0.07 0.54 0.09 87.53

denovo87 0.00 0.00 0.06 0.41 0.09 87.62

denovo1579 0.00 0.00 0.06 0.62 0.09 87.72

denovo473 0.00 0.00 0.06 0.30 0.09 87.81

denovo143 0.00 0.00 0.06 0.38 0.09 87.89

denovo657 0.00 0.00 0.06 0.30 0.09 87.98

denovo1132 0.00 0.00 0.06 0.30 0.09 88.07

HQ245988 0.00 0.00 0.06 0.51 0.09 88.16

denovo676 0.00 0.00 0.06 0.51 0.09 88.25

denovo1131 0.00 0.00 0.06 0.29 0.09 88.33

denovo1923 0.00 0.00 0.06 0.40 0.09 88.42

denovo1459 0.00 0.00 0.06 0.56 0.09 88.50

AF252321 0.00 0.00 0.06 0.30 0.08 88.59

JF712668 0.00 0.00 0.06 0.51 0.08 88.67

denovo815 0.00 0.00 0.06 0.41 0.08 88.75

denovo853 0.00 0.00 0.06 0.56 0.08 88.83

HQ588359 0.00 0.00 0.05 0.29 0.08 88.91

denovo1743 0.00 0.00 0.05 0.41 0.08 88.99

denovo1899 0.00 0.00 0.05 0.40 0.08 89.07

denovo272 0.00 0.00 0.05 0.41 0.08 89.14

denovo1480 0.00 0.00 0.05 0.30 0.08 89.22

denovo990 0.00 0.00 0.05 0.30 0.08 89.29

denovo24 0.00 0.00 0.05 0.43 0.07 89.37

denovo1423 0.00 0.00 0.05 0.44 0.07 89.44

denovo1632 0.00 0.00 0.05 0.42 0.07 89.51

denovo148 0.00 0.00 0.05 0.30 0.07 89.58

denovo1832 0.00 0.00 0.05 0.51 0.07 89.65

denovo34 0.00 0.00 0.05 0.29 0.07 89.71

denovo850 0.00 0.00 0.05 0.52 0.07 89.78

denovo1621 0.00 0.00 0.05 0.40 0.07 89.85

denovo1637 0.00 0.00 0.05 0.42 0.07 89.91

denovo871 0.00 0.00 0.05 0.42 0.07 89.98

HQ326257 0.00 0.00 0.05 0.29 0.07 90.04

Groups marine & mucin

Average dissimilarity = 33.64

Group marine Group mucin

Species Av.Abund Av.Abund Av.Diss Diss/SD Contrib% Cum.%

GQ118701 0.12 0.07 3.23 1.00 9.60 9.60

denovo1026 0.18 0.02 1.02 0.76 3.04 12.64

denovo1017 0.25 0.01 0.98 0.50 2.92 15.56

HE574879 0.02 0.02 0.92 0.83 2.73 18.30

denovo189 0.04 0.02 0.87 1.20 2.59 20.89

denovo1146 0.04 0.02 0.86 1.27 2.57 23.46

denovo1484 0.19 0.02 0.83 0.62 2.48 25.94

JF937422 0.18 0.01 0.70 0.51 2.07 28.01

denovo1749 0.97 0.99 0.68 0.72 2.02 30.03

denovo838 0.18 0.01 0.65 0.47 1.92 31.95

denovo1905 0.25 0.00 0.64 0.32 1.90 33.85

denovo427 0.02 0.02 0.62 1.04 1.83 35.68

denovo109 0.02 0.01 0.61 1.04 1.83 37.51

denovo756 0.18 0.01 0.61 0.43 1.80 39.32

denovo507 0.02 0.01 0.60 1.11 1.78 41.10

JN874385 0.01 0.02 0.58 0.75 1.72 42.81

denovo1249 0.02 0.02 0.58 1.29 1.71 44.52

denovo547 0.01 0.01 0.57 0.90 1.71 46.23

denovo1189 0.02 0.02 0.57 1.18 1.71 47.94

denovo1743 0.18 0.00 0.55 0.39 1.63 49.57

denovo1476 0.18 0.00 0.51 0.36 1.52 51.09

denovo1791 0.18 0.00 0.48 0.34 1.44 52.52

denovo1322 0.18 0.00 0.46 0.32 1.37 53.90

denovo324 0.18 0.00 0.46 0.32 1.36 55.25

denovo114 0.18 0.00 0.45 0.32 1.35 56.60

denovo552 0.01 0.01 0.44 0.78 1.32 57.92

denovo1558 0.00 0.02 0.43 0.43 1.29 59.21

denovo1565 0.18 0.00 0.43 0.30 1.28 60.49

denovo1640 0.18 0.00 0.43 0.30 1.28 61.77

denovo1785 0.18 0.00 0.43 0.30 1.28 63.06

GQ385289 0.18 0.00 0.43 0.30 1.28 64.34

denovo141 0.01 0.01 0.35 1.09 1.04 65.37

denovo204 0.01 0.01 0.33 0.95 0.99 66.36

denovo119 0.01 0.01 0.31 0.85 0.93 67.29

denovo1479 0.00 0.01 0.29 0.67 0.86 68.16

denovo1070 0.01 0.00 0.28 0.84 0.85 69.00

denovo415 0.01 0.01 0.27 1.22 0.79 69.79

denovo953 0.01 0.01 0.26 0.75 0.78 70.58

denovo828 0.00 0.01 0.24 0.42 0.70 71.28

denovo1145 0.01 0.01 0.23 1.20 0.67 71.95

denovo1462 0.01 0.00 0.21 0.79 0.62 72.57

denovo430 0.01 0.01 0.20 1.12 0.60 73.17

denovo545 0.01 0.01 0.20 1.22 0.60 73.77

AM709702 0.00 0.00 0.20 0.44 0.59 74.36

denovo1842 0.01 0.00 0.18 0.78 0.53 74.89

denovo1085 0.00 0.00 0.18 0.76 0.52 75.41

HQ671075 0.01 0.00 0.17 0.34 0.51 75.92

denovo1579 0.00 0.00 0.17 0.95 0.50 76.42

FJ169195 0.00 0.00 0.17 0.66 0.49 76.91

denovo709 0.00 0.00 0.15 0.79 0.46 77.36

HQ616267 0.00 0.00 0.15 0.83 0.44 77.80

denovo198 0.00 0.00 0.14 0.29 0.42 78.22

denovo1789 0.00 0.00 0.14 0.49 0.41 78.63

denovo1292 0.00 0.00 0.13 0.59 0.38 79.01

DQ860060 0.00 0.00 0.13 0.60 0.37 79.38

denovo294 0.00 0.00 0.11 0.42 0.34 79.72

denovo777 0.00 0.00 0.11 0.68 0.34 80.06

denovo1632 0.00 0.00 0.11 0.80 0.32 80.38

EF613488 0.00 0.00 0.11 0.41 0.32 80.70

denovo1880 0.00 0.00 0.10 0.29 0.30 81.00

denovo43 0.00 0.00 0.10 0.78 0.30 81.30

denovo320 0.00 0.00 0.10 0.73 0.29 81.58

HE574926 0.00 0.00 0.09 0.46 0.27 81.86

denovo172 0.00 0.00 0.09 0.53 0.26 82.11

denovo190 0.00 0.00 0.09 0.53 0.25 82.37

denovo1420 0.00 0.00 0.09 0.61 0.25 82.62

denovo1701 0.00 0.00 0.09 0.69 0.25 82.88

HM108471 0.00 0.00 0.08 0.48 0.25 83.13

X97093 0.00 0.00 0.08 0.63 0.25 83.37

denovo1088 0.00 0.00 0.08 0.56 0.25 83.62

denovo790 0.00 0.00 0.08 0.63 0.24 83.86

denovo1230 0.00 0.00 0.07 0.56 0.22 84.08

denovo293 0.00 0.00 0.07 0.52 0.21 84.29

denovo457 0.00 0.00 0.07 0.38 0.21 84.50

denovo1338 0.00 0.00 0.07 0.30 0.20 84.69

EU536078 0.00 0.00 0.06 0.54 0.19 84.89

denovo520 0.00 0.00 0.06 0.30 0.19 85.08

denovo1832 0.00 0.00 0.06 0.53 0.19 85.26

denovo729 0.00 0.00 0.06 0.42 0.19 85.45

denovo719 0.00 0.00 0.06 0.40 0.18 85.63

AY914065 0.00 0.00 0.06 0.52 0.18 85.81

denovo1390 0.00 0.00 0.06 0.38 0.18 85.98

denovo1898 0.00 0.00 0.06 0.52 0.17 86.15

denovo183 0.00 0.00 0.06 0.30 0.17 86.32

denovo815 0.00 0.00 0.06 0.52 0.17 86.49

denovo1517 0.00 0.00 0.06 0.41 0.16 86.65

denovo854 0.00 0.00 0.05 0.44 0.16 86.81

denovo312 0.00 0.00 0.05 0.42 0.16 86.96

denovo1062 0.00 0.00 0.05 0.52 0.15 87.12

HQ721418 0.00 0.00 0.05 0.42 0.15 87.27

denovo60 0.00 0.00 0.05 0.41 0.15 87.42

JN177591 0.00 0.00 0.05 0.29 0.15 87.56

DQ396132 0.00 0.00 0.05 0.30 0.14 87.71

AJ633944 0.00 0.00 0.05 0.30 0.14 87.85

denovo676 0.00 0.00 0.05 0.41 0.14 87.99

denovo1489 0.00 0.00 0.05 0.41 0.14 88.13

denovo1889 0.00 0.00 0.05 0.44 0.14 88.26

EU935300 0.00 0.00 0.05 0.41 0.14 88.40

AF170746 0.00 0.00 0.04 0.43 0.13 88.53

denovo346 0.00 0.00 0.04 0.44 0.13 88.66

denovo853 0.00 0.00 0.04 0.30 0.13 88.79

denovo7 0.00 0.00 0.04 0.41 0.13 88.92

GU305772 0.00 0.00 0.04 0.41 0.13 89.05

DQ446109 0.00 0.00 0.04 0.29 0.13 89.18

denovo1820 0.00 0.00 0.04 0.42 0.13 89.30

denovo1291 0.00 0.00 0.04 0.29 0.13 89.43

AY922243 0.00 0.00 0.04 0.30 0.13 89.56

denovo1850 0.00 0.00 0.04 0.30 0.13 89.68

denovo1694 0.00 0.00 0.04 0.40 0.13 89.81

GU940713 0.00 0.00 0.04 0.42 0.12 89.93

denovo1801 0.00 0.00 0.04 0.42 0.12 90.05

Groups marine_dil & mucin

Average dissimilarity = 80.93

Group marine_dil Group mucin

Species Av.Abund Av.Abund Av.Diss Diss/SD Contrib% Cum.%

denovo1749 0.45 0.99 10.94 1.40 13.52 13.52

denovo75 0.15 0.00 3.24 0.43 4.01 17.53

denovo1026 0.17 0.02 2.97 0.91 3.67 21.20

JN579972 0.11 0.00 2.63 0.38 3.25 24.46

FJ624884 0.13 0.00 2.48 0.64 3.06 27.52

JN030555 0.09 0.00 2.25 0.32 2.78 30.29

JF937422 0.13 0.01 1.76 1.41 2.18 32.47

GQ118701 0.05 0.07 1.30 1.16 1.61 34.08

AY739689 0.05 0.00 1.05 0.30 1.30 35.38

HQ245988 0.05 0.00 0.95 0.31 1.17 36.55

AY914065 0.06 0.00 0.86 1.30 1.06 37.61

AB299573 0.04 0.00 0.76 0.31 0.94 38.55

denovo1484 0.05 0.02 0.76 1.20 0.93 39.48

HM595366 0.04 0.00 0.73 0.71 0.90 40.39

HE574879 0.03 0.02 0.71 0.70 0.88 41.26

EU935300 0.05 0.00 0.69 1.32 0.86 42.12

U81990 0.04 0.00 0.66 0.47 0.81 42.93

denovo1888 0.04 0.00 0.66 0.68 0.81 43.74

EU035954 0.04 0.00 0.62 0.77 0.77 44.51

GU940713 0.04 0.00 0.62 1.01 0.76 45.27

denovo1873 0.03 0.00 0.57 0.44 0.71 45.98

HQ118426 0.03 0.00 0.51 0.65 0.63 46.61

denovo1273 0.03 0.00 0.50 1.53 0.62 47.23

AF286480 0.02 0.00 0.50 0.64 0.61 47.84

DQ889875 0.04 0.00 0.49 0.60 0.61 48.45

AM992178 0.02 0.00 0.49 0.31 0.60 49.05

denovo1720 0.02 0.00 0.46 0.72 0.57 49.62

denovo1146 0.00 0.02 0.45 1.03 0.56 50.18

AJ292596 0.02 0.00 0.45 0.57 0.55 50.73

AM935808 0.03 0.00 0.43 0.30 0.53 51.26

AY631057 0.02 0.00 0.43 0.30 0.53 51.79

denovo427 0.01 0.02 0.43 0.99 0.53 52.32

denovo1022 0.03 0.00 0.40 0.84 0.50 52.81

GU118526 0.03 0.00 0.39 0.49 0.49 53.30

AB176201 0.03 0.00 0.39 1.07 0.48 53.78

denovo189 0.00 0.02 0.38 0.98 0.47 54.25

FQ659744 0.01 0.00 0.38 0.30 0.47 54.73

HQ721418 0.03 0.00 0.38 1.11 0.47 55.20

JN874385 0.01 0.02 0.36 0.75 0.45 55.65

denovo1848 0.02 0.00 0.35 0.30 0.44 56.08

denovo605 0.02 0.00 0.35 0.45 0.43 56.51

denovo792 0.02 0.00 0.35 0.43 0.43 56.94

denovo1249 0.00 0.02 0.34 1.09 0.43 57.37

FJ516885 0.01 0.00 0.31 0.37 0.39 57.76

GU179554 0.02 0.00 0.31 0.93 0.38 58.14

denovo552 0.01 0.01 0.31 0.72 0.38 58.52

AB477015 0.02 0.00 0.31 1.08 0.38 58.90

denovo1547 0.02 0.00 0.30 0.54 0.37 59.27

denovo547 0.01 0.01 0.30 0.72 0.37 59.64

X97093 0.02 0.00 0.29 0.92 0.36 60.00

denovo1558 0.00 0.02 0.28 0.43 0.35 60.35

denovo1189 0.00 0.02 0.28 0.94 0.35 60.70

denovo507 0.01 0.01 0.28 1.03 0.35 61.05

denovo1017 0.00 0.01 0.28 0.81 0.34 61.39

denovo1162 0.02 0.00 0.28 0.53 0.34 61.73

denovo109 0.00 0.01 0.27 1.00 0.33 62.07

denovo1842 0.01 0.00 0.27 0.84 0.33 62.40

denovo2019 0.01 0.00 0.27 0.60 0.33 62.73

denovo550 0.01 0.00 0.25 0.30 0.31 63.04

denovo545 0.00 0.01 0.25 1.80 0.31 63.34

JN868782 0.02 0.00 0.24 1.03 0.30 63.65

denovo1923 0.01 0.00 0.24 0.61 0.30 63.95

denovo1190 0.01 0.00 0.23 0.62 0.29 64.23

AJ347026 0.02 0.00 0.23 0.54 0.29 64.52

AM709702 0.01 0.00 0.23 0.80 0.28 64.80

AF252321 0.01 0.00 0.23 0.30 0.28 65.08

EU160577 0.02 0.00 0.23 0.74 0.28 65.36

denovo1263 0.01 0.00 0.22 0.30 0.27 65.64

FJ418924 0.02 0.00 0.22 0.54 0.27 65.90

denovo1632 0.01 0.00 0.21 0.89 0.25 66.16

denovo776 0.02 0.00 0.20 0.56 0.25 66.41

denovo838 0.00 0.01 0.20 1.86 0.25 66.66

HQ616267 0.01 0.00 0.20 1.00 0.25 66.92

denovo119 0.01 0.01 0.20 0.63 0.25 67.17

denovo822 0.01 0.00 0.20 0.50 0.25 67.41

denovo204 0.01 0.01 0.20 0.90 0.25 67.66

denovo862 0.01 0.00 0.20 0.39 0.24 67.90

denovo1070 0.01 0.00 0.20 0.85 0.24 68.15

denovo607 0.01 0.00 0.20 0.53 0.24 68.39

denovo1182 0.02 0.00 0.20 0.54 0.24 68.63

EU536078 0.01 0.00 0.19 0.64 0.24 68.87

denovo854 0.01 0.00 0.19 0.54 0.23 69.10

denovo415 0.00 0.01 0.18 1.27 0.23 69.33

AF170746 0.02 0.00 0.17 0.50 0.21 69.55

denovo1250 0.01 0.00 0.17 0.75 0.21 69.75

denovo1479 0.00 0.01 0.16 0.68 0.20 69.95

HM108471 0.01 0.00 0.16 0.69 0.20 70.15

denovo141 0.00 0.01 0.16 0.74 0.20 70.35

denovo1577 0.01 0.00 0.16 0.64 0.19 70.55

denovo953 0.00 0.01 0.16 0.70 0.19 70.74

GU118575 0.01 0.00 0.15 0.53 0.19 70.93

GU553034 0.01 0.00 0.15 0.34 0.19 71.12

EU803928 0.01 0.00 0.15 0.57 0.18 71.30

denovo1964 0.01 0.00 0.15 0.57 0.18 71.48

denovo828 0.00 0.01 0.14 0.38 0.18 71.66

denovo61 0.01 0.00 0.14 0.64 0.17 71.84

denovo756 0.00 0.01 0.14 0.68 0.17 72.01

GQ921403 0.01 0.00 0.14 0.30 0.17 72.18

denovo98 0.01 0.00 0.14 0.54 0.17 72.35

denovo1284 0.01 0.00 0.14 0.56 0.17 72.52

FJ892785 0.01 0.00 0.14 0.69 0.17 72.69

denovo1420 0.01 0.00 0.14 0.72 0.17 72.86

JF937433 0.01 0.00 0.14 0.54 0.17 73.03

GU118690 0.01 0.00 0.13 0.56 0.17 73.19

GQ263306 0.01 0.00 0.13 0.69 0.17 73.36

denovo696 0.01 0.00 0.13 0.30 0.16 73.52

denovo663 0.01 0.00 0.13 0.56 0.16 73.69

DQ316817 0.01 0.00 0.13 0.30 0.16 73.85

denovo574 0.01 0.00 0.13 0.47 0.16 74.01

denovo1145 0.00 0.01 0.12 0.89 0.15 74.16

denovo249 0.01 0.00 0.12 0.80 0.15 74.31

DQ889881 0.01 0.00 0.12 0.57 0.14 74.46

denovo198 0.00 0.00 0.11 0.36 0.14 74.60

EU463921 0.01 0.00 0.11 0.45 0.14 74.73

GU118606 0.01 0.00 0.11 0.57 0.14 74.87

DQ860060 0.00 0.00 0.11 0.61 0.14 75.01

FJ900573 0.01 0.00 0.11 0.57 0.13 75.14

GU305772 0.01 0.00 0.11 0.73 0.13 75.27

denovo430 0.00 0.01 0.11 0.76 0.13 75.40

FJ557790 0.01 0.00 0.11 0.51 0.13 75.53

FJ820465 0.01 0.00 0.10 0.44 0.13 75.66

denovo412 0.00 0.00 0.10 0.49 0.13 75.79

denovo423 0.01 0.00 0.10 0.44 0.12 75.91

JF925031 0.01 0.00 0.10 0.64 0.12 76.03

denovo1961 0.01 0.00 0.10 0.44 0.12 76.16

FJ802385 0.01 0.00 0.10 0.56 0.12 76.27

denovo528 0.01 0.00 0.10 0.34 0.12 76.39

denovo305 0.01 0.00 0.09 0.54 0.12 76.51

EF076171 0.01 0.00 0.09 0.64 0.12 76.62

denovo917 0.00 0.00 0.09 0.30 0.12 76.74

denovo788 0.01 0.00 0.09 0.30 0.12 76.85

denovo1480 0.00 0.00 0.09 0.40 0.11 76.97

denovo1898 0.00 0.00 0.09 0.57 0.11 77.08

FJ381979 0.01 0.00 0.09 0.57 0.11 77.19

AY959053 0.01 0.00 0.09 0.57 0.11 77.30

denovo1306 0.00 0.00 0.09 0.42 0.11 77.41

denovo1085 0.00 0.00 0.09 0.60 0.11 77.52

FJ999591 0.01 0.00 0.08 0.50 0.10 77.62

EF018153 0.00 0.00 0.08 0.30 0.10 77.72

denovo1696 0.00 0.00 0.08 0.35 0.10 77.82

denovo1590 0.01 0.00 0.08 0.56 0.10 77.92

HQ753432 0.01 0.00 0.08 0.53 0.10 78.02

denovo88 0.00 0.00 0.08 0.30 0.10 78.12

FJ457296 0.00 0.00 0.08 0.30 0.10 78.21

denovo1526 0.00 0.00 0.08 0.30 0.10 78.31

denovo744 0.00 0.00 0.08 0.43 0.09 78.40

denovo2003 0.01 0.00 0.08 0.44 0.09 78.50

denovo499 0.00 0.00 0.08 0.30 0.09 78.59

denovo1579 0.00 0.00 0.08 0.60 0.09 78.68

denovo659 0.00 0.00 0.08 0.60 0.09 78.78

AJ621576 0.01 0.00 0.07 0.64 0.09 78.87

denovo294 0.00 0.00 0.07 0.42 0.09 78.96

denovo1705 0.00 0.00 0.07 0.30 0.09 79.05

denovo308 0.00 0.00 0.07 0.57 0.09 79.14

denovo165 0.01 0.00 0.07 0.39 0.09 79.23

denovo1414 0.00 0.00 0.07 0.30 0.09 79.32

denovo1743 0.00 0.00 0.07 0.64 0.09 79.41

denovo1093 0.00 0.00 0.07 0.30 0.09 79.50

denovo1556 0.00 0.00 0.07 0.39 0.09 79.59

denovo860 0.00 0.00 0.07 0.30 0.09 79.68

denovo1585 0.00 0.00 0.07 0.43 0.09 79.76

denovo777 0.00 0.00 0.07 0.62 0.08 79.85

EF076172 0.01 0.00 0.07 0.54 0.08 79.93

denovo1688 0.00 0.00 0.07 0.30 0.08 80.01

FJ529262 0.01 0.00 0.07 0.52 0.08 80.10

denovo1462 0.00 0.00 0.07 0.51 0.08 80.18

denovo1703 0.00 0.00 0.07 0.44 0.08 80.26

denovo916 0.00 0.00 0.07 0.44 0.08 80.35

denovo103 0.00 0.00 0.07 0.30 0.08 80.43

denovo1670 0.00 0.00 0.07 0.43 0.08 80.51

denovo1880 0.00 0.00 0.07 0.29 0.08 80.59

HQ672216 0.00 0.00 0.06 0.43 0.08 80.67

denovo521 0.00 0.00 0.06 0.30 0.08 80.75

FQ660217 0.00 0.00 0.06 0.45 0.08 80.83

HQ118340 0.00 0.00 0.06 0.53 0.08 80.91

FJ826409 0.00 0.00 0.06 0.57 0.08 80.99

denovo1303 0.01 0.00 0.06 0.40 0.08 81.07

EU817491 0.00 0.00 0.06 0.43 0.08 81.14

denovo1555 0.00 0.00 0.06 0.40 0.08 81.22

denovo707 0.01 0.00 0.06 0.44 0.08 81.30

AJ583166 0.00 0.00 0.06 0.36 0.07 81.37

JN411267 0.00 0.00 0.06 0.44 0.07 81.45

FJ169195 0.00 0.00 0.06 0.50 0.07 81.52

denovo1641 0.00 0.00 0.06 0.30 0.07 81.59

DQ889935 0.00 0.00 0.06 0.44 0.07 81.66

denovo767 0.00 0.00 0.06 0.30 0.07 81.73

denovo1341 0.00 0.00 0.06 0.30 0.07 81.80

denovo172 0.00 0.00 0.06 0.54 0.07 81.87

denovo1292 0.00 0.00 0.06 0.43 0.07 81.94

denovo1147 0.00 0.00 0.06 0.37 0.07 82.01

AJ863291 0.00 0.00 0.06 0.30 0.07 82.08

denovo1274 0.00 0.00 0.06 0.30 0.07 82.15

denovo1082 0.00 0.00 0.06 0.30 0.07 82.22

denovo1875 0.00 0.00 0.05 0.30 0.07 82.28

denovo40 0.00 0.00 0.05 0.42 0.07 82.35

denovo1265 0.00 0.00 0.05 0.44 0.07 82.41

JN177591 0.00 0.00 0.05 0.41 0.06 82.48

denovo898 0.00 0.00 0.05 0.44 0.06 82.54

HE574926 0.00 0.00 0.05 0.39 0.06 82.61

denovo818 0.00 0.00 0.05 0.30 0.06 82.67

denovo1240 0.00 0.00 0.05 0.43 0.06 82.73

denovo114 0.00 0.00 0.05 0.45 0.06 82.79

denovo1095 0.00 0.00 0.05 0.30 0.06 82.85

HQ616309 0.00 0.00 0.05 0.40 0.06 82.91

denovo1753 0.00 0.00 0.05 0.30 0.06 82.97

denovo624 0.00 0.00 0.05 0.30 0.06 83.03

denovo520 0.00 0.00 0.05 0.45 0.06 83.09

denovo657 0.00 0.00 0.05 0.30 0.06 83.15

denovo727 0.00 0.00 0.05 0.44 0.06 83.21

denovo358 0.00 0.00 0.05 0.30 0.06 83.27

denovo1383 0.00 0.00 0.05 0.30 0.06 83.33

denovo225 0.00 0.00 0.05 0.30 0.06 83.39

FJ497578 0.00 0.00 0.05 0.44 0.06 83.45

denovo543 0.00 0.00 0.05 0.41 0.06 83.51

GU563744 0.00 0.00 0.05 0.43 0.06 83.56

denovo1415 0.00 0.00 0.05 0.41 0.06 83.62

denovo1789 0.00 0.00 0.05 0.42 0.06 83.68

EF153415 0.00 0.00 0.05 0.41 0.06 83.73

GQ903461 0.00 0.00 0.05 0.30 0.06 83.79

denovo1226 0.00 0.00 0.05 0.30 0.06 83.85

denovo819 0.00 0.00 0.05 0.30 0.06 83.90

denovo28 0.00 0.00 0.05 0.30 0.06 83.96

denovo1299 0.00 0.00 0.05 0.30 0.06 84.02

denovo787 0.00 0.00 0.05 0.30 0.06 84.07

denovo918 0.00 0.00 0.05 0.30 0.06 84.13

FJ946588 0.00 0.00 0.05 0.30 0.06 84.18

FN666870 0.00 0.00 0.04 0.30 0.05 84.24

EU794092 0.00 0.00 0.04 0.44 0.05 84.29

denovo244 0.00 0.00 0.04 0.42 0.05 84.34

FJ675001 0.01 0.00 0.04 0.30 0.05 84.40

JF808978 0.00 0.00 0.04 0.44 0.05 84.45

denovo1561 0.00 0.00 0.04 0.30 0.05 84.50

denovo709 0.00 0.00 0.04 0.40 0.05 84.55

denovo431 0.00 0.00 0.04 0.42 0.05 84.61

denovo6 0.00 0.00 0.04 0.30 0.05 84.66

denovo729 0.00 0.00 0.04 0.42 0.05 84.71

denovo769 0.00 0.00 0.04 0.44 0.05 84.76

denovo1401 0.00 0.00 0.04 0.52 0.05 84.81

HQ326290 0.00 0.00 0.04 0.40 0.05 84.86

HQ143274 0.00 0.00 0.04 0.30 0.05 84.91

denovo1855 0.00 0.00 0.04 0.30 0.05 84.96

denovo66 0.00 0.00 0.04 0.30 0.05 85.01

denovo1757 0.00 0.00 0.04 0.39 0.05 85.06

denovo104 0.00 0.00 0.04 0.44 0.05 85.11

denovo1243 0.00 0.00 0.04 0.30 0.05 85.16

denovo1412 0.00 0.00 0.04 0.30 0.05 85.21

denovo1014 0.00 0.00 0.04 0.30 0.05 85.26

denovo993 0.00 0.00 0.04 0.30 0.05 85.30

denovo1139 0.00 0.00 0.04 0.30 0.05 85.35

denovo1443 0.00 0.00 0.04 0.30 0.05 85.40

denovo1911 0.00 0.00 0.04 0.30 0.05 85.45

denovo1937 0.00 0.00 0.04 0.30 0.05 85.50

denovo1998 0.00 0.00 0.04 0.30 0.05 85.55

denovo565 0.00 0.00 0.04 0.30 0.05 85.60

denovo921 0.00 0.00 0.04 0.30 0.05 85.65

denovo1775 0.00 0.00 0.04 0.30 0.05 85.70

denovo260 0.00 0.00 0.04 0.30 0.05 85.75

denovo608 0.00 0.00 0.04 0.42 0.05 85.79

FJ808721 0.00 0.00 0.04 0.43 0.05 85.84

denovo1237 0.00 0.00 0.04 0.30 0.05 85.89

denovo1856 0.00 0.00 0.04 0.30 0.05 85.94

denovo236 0.00 0.00 0.04 0.30 0.05 85.99

denovo94 0.00 0.00 0.04 0.30 0.05 86.03

denovo631 0.00 0.00 0.04 0.30 0.05 86.08

EF613488 0.00 0.00 0.04 0.29 0.05 86.13

denovo648 0.00 0.00 0.04 0.40 0.05 86.18

JN544140 0.00 0.00 0.04 0.45 0.04 86.22

denovo1730 0.00 0.00 0.04 0.41 0.04 86.26

denovo804 0.00 0.00 0.04 0.40 0.04 86.31

denovo432 0.00 0.00 0.04 0.40 0.04 86.35

denovo1457 0.00 0.00 0.04 0.44 0.04 86.40

denovo1476 0.00 0.00 0.03 0.42 0.04 86.44

denovo1930 0.00 0.00 0.03 0.41 0.04 86.48

denovo1936 0.00 0.00 0.03 0.30 0.04 86.53

denovo312 0.00 0.00 0.03 0.42 0.04 86.57

denovo827 0.00 0.00 0.03 0.30 0.04 86.61

denovo2018 0.00 0.00 0.03 0.30 0.04 86.65

denovo1396 0.00 0.00 0.03 0.30 0.04 86.69

denovo457 0.00 0.00 0.03 0.29 0.04 86.74

denovo385 0.00 0.00 0.03 0.30 0.04 86.78

denovo728 0.00 0.00 0.03 0.30 0.04 86.82

GU118588 0.00 0.00 0.03 0.30 0.04 86.86

EU776368 0.00 0.00 0.03 0.30 0.04 86.90

denovo1391 0.00 0.00 0.03 0.44 0.04 86.94

denovo1374 0.00 0.00 0.03 0.30 0.04 86.98

denovo600 0.00 0.00 0.03 0.30 0.04 87.02

denovo1845 0.00 0.00 0.03 0.30 0.04 87.06

denovo977 0.00 0.00 0.03 0.30 0.04 87.10

denovo1643 0.00 0.00 0.03 0.30 0.04 87.14

denovo593 0.00 0.00 0.03 0.30 0.04 87.18

denovo1110 0.00 0.00 0.03 0.30 0.04 87.22

denovo1141 0.00 0.00 0.03 0.30 0.04 87.26

denovo1227 0.00 0.00 0.03 0.30 0.04 87.30

denovo1254 0.00 0.00 0.03 0.30 0.04 87.34

denovo1264 0.00 0.00 0.03 0.30 0.04 87.38

denovo1309 0.00 0.00 0.03 0.30 0.04 87.42

denovo1340 0.00 0.00 0.03 0.30 0.04 87.46

denovo1407 0.00 0.00 0.03 0.30 0.04 87.50

denovo142 0.00 0.00 0.03 0.30 0.04 87.53

denovo1521 0.00 0.00 0.03 0.30 0.04 87.57

denovo1674 0.00 0.00 0.03 0.30 0.04 87.61

denovo1727 0.00 0.00 0.03 0.30 0.04 87.65

denovo1751 0.00 0.00 0.03 0.30 0.04 87.69

denovo1877 0.00 0.00 0.03 0.30 0.04 87.73

denovo1999 0.00 0.00 0.03 0.30 0.04 87.77

denovo25 0.00 0.00 0.03 0.30 0.04 87.81

denovo468 0.00 0.00 0.03 0.30 0.04 87.85

denovo603 0.00 0.00 0.03 0.30 0.04 87.89

denovo618 0.00 0.00 0.03 0.30 0.04 87.93

denovo717 0.00 0.00 0.03 0.30 0.04 87.97

GU941113 0.00 0.00 0.03 0.30 0.04 88.01

denovo1385 0.00 0.00 0.03 0.30 0.04 88.05

denovo161 0.00 0.00 0.03 0.30 0.04 88.09

denovo332 0.00 0.00 0.03 0.30 0.04 88.13

denovo428 0.00 0.00 0.03 0.30 0.04 88.17

denovo535 0.00 0.00 0.03 0.30 0.04 88.21

FJ957596 0.00 0.00 0.03 0.44 0.04 88.25

denovo2047 0.00 0.00 0.03 0.30 0.04 88.28

HQ757459 0.00 0.00 0.03 0.30 0.04 88.32

denovo1824 0.00 0.00 0.03 0.30 0.04 88.36

EU794074 0.00 0.00 0.03 0.30 0.04 88.40

GQ433928 0.00 0.00 0.03 0.42 0.04 88.43

denovo797 0.00 0.00 0.03 0.30 0.04 88.47

denovo1633 0.00 0.00 0.03 0.30 0.04 88.50

HQ323455 0.00 0.00 0.03 0.30 0.04 88.54

denovo1584 0.00 0.00 0.03 0.30 0.04 88.57

denovo1518 0.00 0.00 0.03 0.30 0.04 88.61

AB084524 0.00 0.00 0.03 0.30 0.04 88.64

denovo1491 0.00 0.00 0.03 0.30 0.04 88.68

FJ269073 0.00 0.00 0.03 0.30 0.04 88.72

denovo2022 0.00 0.00 0.03 0.30 0.04 88.75

AF332511 0.00 0.00 0.03 0.30 0.04 88.79

denovo1764 0.00 0.00 0.03 0.30 0.04 88.82

denovo1940 0.00 0.00 0.03 0.30 0.04 88.86

denovo195 0.00 0.00 0.03 0.30 0.04 88.89

denovo2011 0.00 0.00 0.03 0.30 0.04 88.93

denovo2052 0.00 0.00 0.03 0.30 0.04 88.96

denovo2058 0.00 0.00 0.03 0.30 0.04 89.00

denovo479 0.00 0.00 0.03 0.30 0.04 89.03

denovo522 0.00 0.00 0.03 0.30 0.04 89.07

denovo583 0.00 0.00 0.03 0.30 0.04 89.11

denovo760 0.00 0.00 0.03 0.30 0.04 89.14

denovo85 0.00 0.00 0.03 0.30 0.04 89.18

denovo998 0.00 0.00 0.03 0.30 0.04 89.21

DQ823929 0.00 0.00 0.03 0.30 0.04 89.25

EF020124 0.00 0.00 0.03 0.30 0.04 89.28

EF116592 0.00 0.00 0.03 0.30 0.04 89.32

EU373552 0.00 0.00 0.03 0.30 0.04 89.35

EU982444 0.00 0.00 0.03 0.30 0.04 89.39

FJ557898 0.00 0.00 0.03 0.30 0.04 89.43

FJ666157 0.00 0.00 0.03 0.30 0.04 89.46

FN401326 0.00 0.00 0.03 0.30 0.04 89.50

HQ856409 0.00 0.00 0.03 0.30 0.04 89.53

JQ311869 0.00 0.00 0.03 0.30 0.04 89.57

U87819 0.00 0.00 0.03 0.30 0.04 89.60

AJ867671 0.00 0.00 0.03 0.41 0.04 89.64

denovo1467 0.00 0.00 0.03 0.43 0.04 89.67

EU844911 0.00 0.00 0.03 0.43 0.04 89.71

JL812080 0.00 0.00 0.03 0.43 0.04 89.74

denovo1820 0.00 0.00 0.03 0.42 0.03 89.78

DQ446109 0.00 0.00 0.03 0.29 0.03 89.81

denovo1291 0.00 0.00 0.03 0.29 0.03 89.85

denovo486 0.00 0.00 0.03 0.30 0.03 89.88

GQ449231 0.00 0.00 0.03 0.30 0.03 89.91

JN397809 0.00 0.00 0.03 0.30 0.03 89.95

denovo1203 0.00 0.00 0.03 0.30 0.03 89.98

denovo690 0.00 0.00 0.03 0.30 0.03 90.02

Groups mueller_hinton & mucin

Average dissimilarity = 59.24

Group mueller_hinton Group mucin

Species Av.Abund Av.Abund Av.Diss Diss/SD Contrib% Cum.%

denovo1749 0.60 0.99 9.76 0.82 16.48 16.48

FJ516885 0.08 0.00 2.88 0.30 4.86 21.34

HM595366 0.13 0.00 2.55 0.58 4.30 25.64

denovo1026 0.12 0.02 2.47 0.88 4.18 29.82

JN579972 0.08 0.00 2.47 0.32 4.17 33.99

denovo1888 0.12 0.00 2.30 0.59 3.88 37.87

GQ118701 0.03 0.07 1.96 1.14 3.31 41.18

HE574879 0.07 0.02 1.77 0.64 3.00 44.17

U81990 0.07 0.00 1.41 0.57 2.39 46.56

denovo1484 0.04 0.02 0.97 1.00 1.64 48.20

denovo605 0.04 0.00 0.82 0.43 1.38 49.58

denovo552 0.03 0.01 0.77 0.61 1.30 50.88

JF937422 0.03 0.01 0.68 1.57 1.15 52.03

denovo862 0.03 0.00 0.65 0.45 1.10 53.13

denovo427 0.01 0.02 0.65 0.98 1.09 54.22

denovo1146 0.00 0.02 0.63 1.13 1.07 55.29

JN874385 0.01 0.02 0.57 0.69 0.96 56.25

denovo1961 0.03 0.00 0.57 0.57 0.96 57.20

denovo189 0.00 0.02 0.55 1.06 0.93 58.14

denovo1243 0.03 0.00 0.54 0.56 0.91 59.05

denovo547 0.01 0.01 0.52 0.78 0.88 59.93

denovo1249 0.00 0.02 0.49 1.19 0.82 60.75

AB176201 0.02 0.00 0.44 0.61 0.74 61.50

denovo1558 0.00 0.02 0.44 0.47 0.74 62.24

denovo1189 0.00 0.02 0.41 1.03 0.70 62.94

denovo507 0.00 0.01 0.40 1.00 0.67 63.60

denovo109 0.00 0.01 0.39 1.00 0.66 64.27

denovo1022 0.02 0.00 0.36 0.43 0.61 64.88

denovo1017 0.00 0.01 0.35 0.84 0.60 65.48

denovo119 0.01 0.01 0.31 0.73 0.53 66.00

EU536078 0.01 0.00 0.31 0.59 0.53 66.53

HQ323455 0.01 0.00 0.30 0.30 0.50 67.03

denovo545 0.01 0.01 0.29 1.64 0.49 67.52

denovo1129 0.01 0.00 0.28 0.57 0.47 67.99

denovo854 0.01 0.00 0.27 0.43 0.45 68.44

denovo1703 0.01 0.00 0.26 0.51 0.44 68.88

denovo415 0.01 0.01 0.25 1.35 0.43 69.31

denovo1412 0.01 0.00 0.25 0.43 0.42 69.72

denovo1479 0.00 0.01 0.25 0.70 0.41 70.14

denovo114 0.01 0.00 0.24 0.43 0.41 70.55

denovo1273 0.01 0.00 0.24 0.48 0.40 70.95

denovo953 0.00 0.01 0.24 0.63 0.40 71.35

denovo141 0.00 0.01 0.24 0.75 0.40 71.75

denovo838 0.00 0.01 0.23 1.48 0.38 72.13

denovo927 0.01 0.00 0.23 0.44 0.38 72.51

denovo1070 0.01 0.00 0.22 0.67 0.37 72.88

denovo1753 0.01 0.00 0.21 0.30 0.35 73.24

denovo1491 0.01 0.00 0.21 0.30 0.35 73.59

FJ849072 0.01 0.00 0.21 0.30 0.35 73.95

denovo204 0.00 0.01 0.20 0.79 0.34 74.29

denovo1096 0.01 0.00 0.20 0.42 0.34 74.63

HM108471 0.01 0.00 0.20 0.62 0.33 74.96

denovo1145 0.00 0.01 0.18 0.92 0.31 75.27

GU940713 0.01 0.00 0.18 0.38 0.31 75.58

AY914065 0.01 0.00 0.18 0.84 0.30 75.88

AF170746 0.01 0.00 0.17 0.35 0.29 76.16

denovo1374 0.01 0.00 0.17 0.30 0.29 76.45

JF802167 0.01 0.00 0.17 0.34 0.28 76.73

denovo756 0.00 0.01 0.17 0.58 0.28 77.01

denovo95 0.01 0.00 0.16 0.30 0.28 77.29

AF286480 0.01 0.00 0.16 0.38 0.28 77.57

denovo1873 0.00 0.00 0.16 0.42 0.27 77.84

denovo767 0.01 0.00 0.16 0.44 0.27 78.11

denovo828 0.00 0.01 0.16 0.29 0.27 78.38

denovo430 0.00 0.01 0.16 0.78 0.27 78.64

JF937433 0.01 0.00 0.15 0.43 0.26 78.90

denovo1446 0.01 0.00 0.15 0.43 0.26 79.16

denovo916 0.01 0.00 0.15 0.30 0.25 79.41

denovo1570 0.01 0.00 0.15 0.30 0.25 79.66

denovo2056 0.01 0.00 0.15 0.30 0.25 79.91

denovo757 0.01 0.00 0.15 0.30 0.25 80.16

JQ032050 0.01 0.00 0.15 0.30 0.25 80.41

denovo2029 0.01 0.00 0.14 0.42 0.24 80.65

denovo1341 0.01 0.00 0.14 0.42 0.24 80.89

denovo1085 0.00 0.00 0.14 0.61 0.23 81.13

denovo1579 0.00 0.00 0.14 0.81 0.23 81.36

denovo2003 0.01 0.00 0.14 0.44 0.23 81.59

denovo198 0.00 0.00 0.13 0.29 0.23 81.81

denovo1462 0.00 0.00 0.13 0.60 0.23 82.04

denovo1921 0.01 0.00 0.13 0.30 0.23 82.27

AB294294 0.00 0.00 0.13 0.38 0.22 82.48

denovo6 0.00 0.00 0.13 0.30 0.21 82.69

GQ433928 0.00 0.00 0.13 0.35 0.21 82.91

denovo294 0.00 0.00 0.12 0.49 0.21 83.12

EU935300 0.00 0.00 0.12 0.71 0.20 83.32

HE574926 0.00 0.00 0.12 0.50 0.20 83.51

DQ860060 0.00 0.00 0.11 0.52 0.19 83.70

denovo1743 0.00 0.00 0.11 0.66 0.18 83.88

denovo1292 0.00 0.00 0.11 0.53 0.18 84.05

denovo679 0.00 0.00 0.10 0.37 0.17 84.23

denovo777 0.00 0.00 0.10 0.63 0.17 84.40

denovo1632 0.00 0.00 0.10 0.79 0.17 84.57

denovo709 0.00 0.00 0.10 0.50 0.17 84.73

denovo1880 0.00 0.00 0.10 0.29 0.16 84.90

denovo1420 0.00 0.00 0.10 0.69 0.16 85.06

denovo706 0.00 0.00 0.09 0.30 0.16 85.22

denovo639 0.00 0.00 0.09 0.30 0.16 85.38

FN396956 0.00 0.00 0.09 0.30 0.16 85.54

denovo1500 0.00 0.00 0.09 0.30 0.16 85.70

denovo1619 0.00 0.00 0.09 0.43 0.15 85.85

denovo1842 0.00 0.00 0.09 0.53 0.15 86.00

AJ240982 0.00 0.00 0.08 0.30 0.14 86.14

denovo172 0.00 0.00 0.08 0.55 0.14 86.28

HQ616267 0.00 0.00 0.08 0.51 0.14 86.42

denovo729 0.00 0.00 0.08 0.52 0.13 86.56

denovo1577 0.00 0.00 0.08 0.30 0.13 86.69

GU179554 0.00 0.00 0.08 0.55 0.13 86.82

AM709702 0.00 0.00 0.07 0.38 0.12 86.95

denovo1131 0.00 0.00 0.07 0.30 0.12 87.07

denovo815 0.00 0.00 0.07 0.50 0.12 87.19

denovo657 0.00 0.00 0.07 0.30 0.12 87.31

denovo1132 0.00 0.00 0.07 0.30 0.12 87.42

denovo190 0.00 0.00 0.07 0.42 0.12 87.54

HQ588359 0.00 0.00 0.07 0.30 0.11 87.65

denovo87 0.00 0.00 0.06 0.39 0.11 87.76

DQ889875 0.00 0.00 0.06 0.53 0.10 87.86

denovo1480 0.00 0.00 0.06 0.30 0.10 87.96

denovo990 0.00 0.00 0.06 0.30 0.10 88.06

FJ169195 0.00 0.00 0.06 0.40 0.10 88.16

EF613488 0.00 0.00 0.06 0.29 0.10 88.26

denovo34 0.00 0.00 0.06 0.30 0.10 88.35

HQ326290 0.00 0.00 0.06 0.40 0.10 88.45

HQ245988 0.00 0.00 0.06 0.43 0.09 88.54

denovo1791 0.00 0.00 0.06 0.41 0.09 88.63

denovo871 0.00 0.00 0.06 0.42 0.09 88.73

HQ326257 0.00 0.00 0.05 0.30 0.09 88.82

denovo43 0.00 0.00 0.05 0.52 0.09 88.91

denovo1476 0.00 0.00 0.05 0.43 0.09 88.99

denovo312 0.00 0.00 0.05 0.43 0.09 89.08

JN030555 0.00 0.00 0.05 0.42 0.09 89.16

HQ721418 0.00 0.00 0.05 0.41 0.08 89.25

denovo457 0.00 0.00 0.05 0.29 0.08 89.33

denovo1150 0.00 0.00 0.05 0.30 0.08 89.42

denovo1923 0.00 0.00 0.05 0.30 0.08 89.50

denovo2018 0.00 0.00 0.05 0.30 0.08 89.58

AB176169 0.00 0.00 0.05 0.30 0.08 89.66

denovo147 0.00 0.00 0.05 0.30 0.08 89.75

denovo199 0.00 0.00 0.05 0.30 0.08 89.83

EU539909 0.00 0.00 0.05 0.30 0.08 89.91

denovo1832 0.00 0.00 0.05 0.42 0.08 89.99

denovo859 0.00 0.00 0.05 0.44 0.08 90.07

Groups mueller_hinton_dil & mucin

Average dissimilarity = 46.74

Group mueller_hinton_dil Group mucin

Species Av.Abund Av.Abund Av.Diss Diss/SD Contrib% Cum.%

denovo1749 0.81 0.99 4.01 0.67 8.57 8.57

GQ118701 0.13 0.07 3.65 1.04 7.82 16.39

HE574879 0.07 0.02 1.83 0.81 3.92 20.31

FJ751910 0.06 0.00 1.39 0.30 2.98 23.28

JF802167 0.05 0.00 1.13 0.30 2.42 25.71

denovo427 0.05 0.02 1.12 1.11 2.40 28.11

AM709702 0.05 0.00 1.12 0.48 2.40 30.51

denovo75 0.05 0.00 0.97 0.31 2.08 32.59

JN874385 0.03 0.02 0.90 0.85 1.92 34.51

denovo1146 0.03 0.02 0.89 1.25 1.91 36.42

denovo547 0.04 0.01 0.89 1.02 1.90 38.32

EU846601 0.04 0.00 0.88 0.30 1.88 40.20

denovo189 0.03 0.02 0.87 1.17 1.86 42.06

denovo1026 0.03 0.02 0.86 1.28 1.84 43.90

denovo552 0.03 0.01 0.86 0.81 1.84 45.74

denovo1479 0.03 0.01 0.63 0.75 1.36 47.10

denovo1189 0.02 0.02 0.61 1.14 1.31 48.40

denovo507 0.02 0.01 0.60 1.11 1.29 49.70

denovo109 0.02 0.01 0.60 1.11 1.28 50.98

denovo1249 0.02 0.02 0.57 1.29 1.21 52.19

denovo1558 0.01 0.02 0.54 0.54 1.16 53.35

denovo1484 0.02 0.02 0.54 1.05 1.16 54.51

JN579972 0.02 0.00 0.54 0.47 1.15 55.66

denovo119 0.02 0.01 0.53 0.81 1.14 56.80

denovo1017 0.01 0.01 0.45 0.93 0.97 57.78

denovo953 0.02 0.01 0.45 0.83 0.96 58.74

denovo828 0.01 0.01 0.45 0.48 0.96 59.70

denovo141 0.01 0.01 0.33 0.99 0.71 60.41

denovo1085 0.01 0.00 0.31 1.04 0.67 61.07

JF802166 0.01 0.00 0.31 0.30 0.66 61.73

denovo1292 0.01 0.00 0.30 0.67 0.63 62.36

denovo204 0.01 0.01 0.28 0.71 0.59 62.96

FJ957855 0.01 0.00 0.27 0.30 0.58 63.53

denovo1462 0.01 0.00 0.27 0.87 0.57 64.11

denovo198 0.01 0.00 0.26 0.43 0.55 64.66

denovo1145 0.01 0.01 0.25 1.14 0.54 65.19

denovo545 0.01 0.01 0.25 1.94 0.53 65.72

denovo293 0.01 0.00 0.23 0.34 0.50 66.22

FJ957708 0.01 0.00 0.23 0.30 0.49 66.71

denovo838 0.00 0.01 0.23 1.57 0.49 67.20

denovo415 0.00 0.01 0.22 1.21 0.48 67.68

denovo430 0.01 0.01 0.22 1.02 0.46 68.14

DQ860060 0.01 0.00 0.21 0.70 0.45 68.60

denovo1996 0.01 0.00 0.21 0.30 0.44 69.04

JF937422 0.01 0.01 0.20 0.76 0.44 69.48

denovo1070 0.01 0.00 0.20 0.76 0.43 69.90

denovo1306 0.01 0.00 0.19 0.30 0.41 70.31

AF286480 0.01 0.00 0.19 0.44 0.41 70.72

denovo1193 0.01 0.00 0.18 0.30 0.39 71.12

denovo709 0.01 0.00 0.18 0.85 0.39 71.50

AJ633944 0.01 0.00 0.18 0.48 0.38 71.89

denovo756 0.00 0.01 0.17 0.66 0.37 72.26

denovo40 0.01 0.00 0.16 0.30 0.35 72.61

AY914065 0.01 0.00 0.16 0.55 0.35 72.95

HE574926 0.01 0.00 0.16 0.61 0.35 73.30

denovo1478 0.01 0.00 0.16 0.30 0.34 73.64

denovo1592 0.01 0.00 0.14 0.30 0.29 73.93

denovo325 0.01 0.00 0.14 0.30 0.29 74.22

denovo1743 0.00 0.00 0.13 0.77 0.28 74.49

denovo1390 0.01 0.00 0.12 0.68 0.26 74.76

denovo1632 0.00 0.00 0.12 0.87 0.25 75.01

denovo1379 0.01 0.00 0.11 0.63 0.25 75.25

EU536078 0.00 0.00 0.11 0.75 0.24 75.50

denovo1579 0.00 0.00 0.11 0.67 0.24 75.74

EU935300 0.00 0.00 0.11 0.43 0.24 75.98

denovo790 0.00 0.00 0.11 0.62 0.24 76.22

GQ903461 0.01 0.00 0.11 0.30 0.24 76.46

FJ203135 0.01 0.00 0.11 0.30 0.24 76.69

FJ826409 0.00 0.00 0.11 0.42 0.23 76.92

denovo1842 0.00 0.00 0.10 0.69 0.22 77.15

denovo294 0.00 0.00 0.10 0.43 0.22 77.37

denovo605 0.00 0.00 0.10 0.43 0.22 77.59

denovo190 0.00 0.00 0.10 0.73 0.22 77.80

denovo1420 0.00 0.00 0.10 0.71 0.21 78.02

denovo324 0.00 0.00 0.10 0.65 0.21 78.23

denovo1374 0.00 0.00 0.10 0.37 0.21 78.44

X97093 0.00 0.00 0.10 0.37 0.21 78.65

HQ616267 0.00 0.00 0.10 0.37 0.21 78.86

denovo1757 0.00 0.00 0.10 0.36 0.20 79.06

denovo777 0.00 0.00 0.10 0.64 0.20 79.26

denovo1880 0.00 0.00 0.09 0.29 0.20 79.46

denovo792 0.00 0.00 0.09 0.30 0.19 79.65

denovo1282 0.00 0.00 0.09 0.60 0.19 79.84

denovo1983 0.00 0.00 0.09 0.55 0.19 80.03

denovo172 0.00 0.00 0.09 0.61 0.19 80.21

denovo193 0.00 0.00 0.09 0.56 0.19 80.40

AY631057 0.00 0.00 0.09 0.30 0.18 80.58

denovo1888 0.00 0.00 0.08 0.63 0.18 80.76

HE574895 0.00 0.00 0.08 0.30 0.18 80.94

denovo1321 0.00 0.00 0.08 0.44 0.18 81.11

HQ326290 0.00 0.00 0.08 0.37 0.17 81.28

denovo1577 0.00 0.00 0.08 0.30 0.17 81.45

denovo1199 0.00 0.00 0.08 0.30 0.17 81.62

denovo1298 0.00 0.00 0.08 0.30 0.17 81.79

denovo1830 0.00 0.00 0.08 0.30 0.17 81.96

denovo1892 0.00 0.00 0.08 0.30 0.17 82.12

denovo481 0.00 0.00 0.08 0.30 0.17 82.29

denovo694 0.00 0.00 0.08 0.30 0.17 82.46

denovo946 0.00 0.00 0.08 0.30 0.17 82.63

DQ833490 0.00 0.00 0.08 0.30 0.17 82.80

HM243844 0.00 0.00 0.08 0.30 0.17 82.96

AB176201 0.00 0.00 0.08 0.56 0.17 83.13

denovo7 0.00 0.00 0.08 0.63 0.17 83.30

denovo1244 0.00 0.00 0.08 0.30 0.17 83.47

denovo1476 0.00 0.00 0.08 0.62 0.17 83.63

GU584610 0.00 0.00 0.08 0.41 0.16 83.79

denovo729 0.00 0.00 0.07 0.52 0.16 83.95

denovo457 0.00 0.00 0.07 0.41 0.16 84.11

denovo499 0.00 0.00 0.07 0.30 0.16 84.27

denovo611 0.00 0.00 0.07 0.30 0.16 84.43

denovo1459 0.00 0.00 0.07 0.64 0.16 84.58

denovo473 0.00 0.00 0.07 0.30 0.15 84.73

denovo1436 0.00 0.00 0.07 0.30 0.15 84.88

denovo676 0.00 0.00 0.07 0.52 0.15 85.02

HM595366 0.00 0.00 0.07 0.63 0.14 85.16

denovo114 0.00 0.00 0.07 0.44 0.14 85.31

AF252321 0.00 0.00 0.07 0.30 0.14 85.45

denovo815 0.00 0.00 0.06 0.51 0.14 85.58

denovo1801 0.00 0.00 0.06 0.61 0.14 85.72

denovo853 0.00 0.00 0.06 0.57 0.13 85.85

denovo312 0.00 0.00 0.06 0.52 0.13 85.98

denovo1694 0.00 0.00 0.06 0.50 0.13 86.11

denovo272 0.00 0.00 0.06 0.42 0.13 86.23

HM108471 0.00 0.00 0.06 0.38 0.12 86.36

denovo1944 0.00 0.00 0.06 0.52 0.12 86.48

denovo43 0.00 0.00 0.06 0.51 0.12 86.60

denovo24 0.00 0.00 0.06 0.44 0.12 86.72

denovo1118 0.00 0.00 0.06 0.40 0.12 86.84

denovo492 0.00 0.00 0.06 0.51 0.12 86.96

denovo1423 0.00 0.00 0.05 0.45 0.12 87.08

FJ169195 0.00 0.00 0.05 0.40 0.12 87.19

denovo1291 0.00 0.00 0.05 0.40 0.12 87.31

denovo854 0.00 0.00 0.05 0.30 0.11 87.42

denovo148 0.00 0.00 0.05 0.30 0.11 87.54

denovo1962 0.00 0.00 0.05 0.53 0.11 87.65

denovo143 0.00 0.00 0.05 0.30 0.11 87.77

EF613488 0.00 0.00 0.05 0.29 0.11 87.88

denovo862 0.00 0.00 0.05 0.44 0.11 87.99

denovo1839 0.00 0.00 0.05 0.30 0.11 88.10

denovo1637 0.00 0.00 0.05 0.43 0.11 88.21

denovo1754 0.00 0.00 0.05 0.41 0.11 88.32

denovo210 0.00 0.00 0.05 0.41 0.11 88.42

denovo1832 0.00 0.00 0.05 0.52 0.11 88.53

denovo1523 0.00 0.00 0.05 0.30 0.10 88.63

GU584786 0.00 0.00 0.05 0.44 0.10 88.73

denovo708 0.00 0.00 0.05 0.30 0.10 88.83

denovo1898 0.00 0.00 0.05 0.52 0.10 88.93

denovo887 0.00 0.00 0.05 0.42 0.10 89.04

denovo1062 0.00 0.00 0.05 0.52 0.10 89.14

denovo1346 0.00 0.00 0.05 0.52 0.10 89.23

denovo1740 0.00 0.00 0.05 0.42 0.10 89.33

denovo872 0.00 0.00 0.04 0.42 0.10 89.43

JN177591 0.00 0.00 0.04 0.29 0.10 89.52

denovo1959 0.00 0.00 0.04 0.40 0.09 89.62

denovo1655 0.00 0.00 0.04 0.43 0.09 89.71

denovo1653 0.00 0.00 0.04 0.44 0.09 89.81

denovo32 0.00 0.00 0.04 0.44 0.09 89.90

denovo1759 0.00 0.00 0.04 0.52 0.09 89.99

denovo1899 0.00 0.00 0.04 0.30 0.09 90.08

**(C)**

Groups A (agar) & F (a filter on top of agar)

Average dissimilarity = 63.16

Group A Group F

Species Av.Abund Av.Abund Av.Diss Diss/SD Contrib% Cum.%

denovo1749 0.75 0.78 6.72 0.80 10.64 10.64

GQ118701 0.09 0.08 2.44 0.81 3.87 14.50

denovo1026 0.07 0.14 2.11 0.81 3.34 17.84

JN579972 0.05 0.04 1.83 0.32 2.90 20.74

denovo75 0.03 0.05 1.42 0.30 2.25 22.99

HE574879 0.05 0.04 1.40 0.67 2.21 25.20

HM595366 0.04 0.03 1.11 0.41 1.76 26.97

FJ516885 0.00 0.03 1.06 0.20 1.68 28.64

denovo1888 0.04 0.02 1.01 0.42 1.60 30.24

JF937422 0.04 0.10 0.96 0.78 1.51 31.75

denovo1484 0.03 0.10 0.86 0.82 1.36 33.11

FJ624884 0.03 0.02 0.84 0.35 1.33 34.44

JN030555 0.00 0.03 0.78 0.20 1.24 35.68

U81990 0.03 0.01 0.70 0.42 1.11 36.79

denovo427 0.02 0.02 0.66 0.85 1.05 37.84

denovo552 0.02 0.02 0.64 0.65 1.01 38.84

denovo1146 0.02 0.02 0.63 0.91 0.99 39.83

denovo189 0.02 0.02 0.60 0.84 0.95 40.79

denovo547 0.01 0.02 0.55 0.80 0.87 41.66

AM709702 0.01 0.02 0.51 0.37 0.81 42.46

denovo1017 0.01 0.11 0.51 0.39 0.81 43.27

JN874385 0.01 0.01 0.50 0.62 0.79 44.06

FJ751910 0.02 0.00 0.49 0.18 0.78 44.84

JF802167 0.02 0.00 0.45 0.20 0.71 45.55

denovo605 0.02 0.01 0.43 0.36 0.68 46.23

denovo109 0.01 0.01 0.43 0.80 0.68 46.90

denovo1249 0.01 0.01 0.42 1.00 0.67 47.57

denovo507 0.01 0.01 0.42 0.81 0.66 48.24

denovo1189 0.01 0.01 0.41 0.84 0.64 48.88

AY914065 0.02 0.01 0.37 0.73 0.59 49.47

AY739689 0.02 0.00 0.37 0.18 0.59 50.06

HQ245988 0.02 0.00 0.36 0.20 0.57 50.63

denovo119 0.01 0.01 0.35 0.70 0.55 51.18

denovo838 0.00 0.08 0.32 0.36 0.50 51.68

denovo1479 0.01 0.01 0.31 0.54 0.50 52.18

denovo862 0.01 0.01 0.31 0.34 0.49 52.67

EU846601 0.00 0.02 0.31 0.18 0.49 53.15

EU935300 0.01 0.01 0.30 0.70 0.47 53.62

denovo114 0.00 0.07 0.29 0.31 0.46 54.09

denovo756 0.00 0.07 0.28 0.30 0.44 54.53

AB176201 0.01 0.01 0.28 0.60 0.44 54.96

AF286480 0.01 0.01 0.27 0.49 0.43 55.39

AB299573 0.00 0.02 0.27 0.19 0.43 55.82

GU940713 0.01 0.01 0.27 0.54 0.42 56.24

denovo953 0.01 0.01 0.27 0.64 0.42 56.66

denovo1022 0.01 0.01 0.26 0.46 0.41 57.07

denovo1873 0.00 0.01 0.26 0.33 0.41 57.48

denovo1905 0.00 0.10 0.26 0.20 0.40 57.88

denovo204 0.01 0.01 0.24 0.73 0.39 58.27

denovo1558 0.01 0.00 0.24 0.34 0.38 58.65

denovo1070 0.01 0.01 0.23 0.79 0.37 59.02

denovo1273 0.01 0.01 0.23 0.62 0.37 59.39

denovo1961 0.01 0.00 0.23 0.38 0.37 59.75

denovo1743 0.00 0.07 0.23 0.26 0.37 60.12

EU035954 0.01 0.01 0.22 0.41 0.35 60.47

denovo545 0.01 0.01 0.22 1.23 0.35 60.82

denovo141 0.01 0.01 0.22 0.74 0.35 61.16

denovo1476 0.00 0.07 0.22 0.24 0.34 61.50

denovo828 0.00 0.01 0.21 0.36 0.33 61.83

denovo1791 0.00 0.07 0.21 0.23 0.33 62.16

denovo324 0.00 0.07 0.21 0.23 0.33 62.49

denovo1243 0.01 0.00 0.20 0.33 0.31 62.80

DQ889875 0.01 0.00 0.19 0.37 0.30 63.10

denovo415 0.01 0.01 0.19 0.98 0.30 63.40

EU536078 0.00 0.01 0.19 0.52 0.30 63.70

AY631057 0.01 0.00 0.18 0.21 0.29 63.99

denovo1322 0.00 0.07 0.18 0.20 0.28 64.27

denovo854 0.01 0.00 0.18 0.46 0.28 64.55

HQ118426 0.01 0.00 0.17 0.35 0.28 64.83

AM992178 0.00 0.01 0.17 0.19 0.27 65.10

denovo1842 0.01 0.00 0.17 0.71 0.27 65.37

denovo1085 0.00 0.00 0.17 0.71 0.27 65.64

denovo1565 0.00 0.07 0.17 0.19 0.27 65.90

denovo1640 0.00 0.07 0.17 0.19 0.27 66.17

denovo1785 0.00 0.07 0.17 0.19 0.27 66.44

GQ385289 0.00 0.07 0.17 0.19 0.27 66.70

denovo1720 0.00 0.01 0.17 0.41 0.26 66.97

denovo1145 0.01 0.00 0.16 0.83 0.26 67.23

denovo1462 0.00 0.00 0.16 0.65 0.26 67.48

HQ721418 0.01 0.01 0.16 0.61 0.25 67.73

denovo792 0.01 0.00 0.16 0.31 0.25 67.98

AM935808 0.00 0.01 0.16 0.18 0.25 68.23

AJ292596 0.00 0.00 0.15 0.32 0.24 68.47

HQ616267 0.00 0.01 0.15 0.73 0.24 68.71

X97093 0.01 0.01 0.15 0.59 0.24 68.94

denovo1292 0.00 0.00 0.14 0.48 0.22 69.17

GU118526 0.01 0.00 0.14 0.28 0.22 69.39

denovo430 0.00 0.00 0.14 0.76 0.22 69.60

HM108471 0.01 0.00 0.14 0.57 0.21 69.82

DQ860060 0.00 0.00 0.13 0.59 0.21 70.03

AF170746 0.01 0.00 0.13 0.38 0.21 70.24

FQ659744 0.00 0.01 0.13 0.18 0.21 70.44

GU179554 0.01 0.00 0.13 0.54 0.20 70.65

denovo1848 0.01 0.00 0.13 0.18 0.20 70.85

denovo1547 0.01 0.00 0.12 0.34 0.19 71.03

denovo709 0.00 0.00 0.12 0.65 0.18 71.21

denovo1632 0.00 0.00 0.12 0.64 0.18 71.40

AB477015 0.01 0.00 0.12 0.55 0.18 71.58

HQ323455 0.00 0.01 0.11 0.20 0.18 71.76

denovo198 0.00 0.00 0.11 0.28 0.18 71.94

denovo1753 0.00 0.01 0.11 0.27 0.18 72.11

denovo1703 0.01 0.00 0.11 0.35 0.18 72.29

denovo1162 0.01 0.00 0.11 0.33 0.17 72.47

JF802166 0.01 0.00 0.11 0.18 0.17 72.64

denovo1374 0.00 0.00 0.11 0.31 0.17 72.80

denovo1923 0.00 0.00 0.11 0.40 0.17 72.97

AF252321 0.01 0.00 0.10 0.22 0.16 73.13

denovo1577 0.00 0.00 0.10 0.42 0.16 73.30

JF937433 0.00 0.00 0.10 0.41 0.16 73.46

denovo1412 0.00 0.00 0.10 0.29 0.16 73.62

denovo1306 0.00 0.00 0.10 0.25 0.15 73.77

denovo2019 0.00 0.00 0.10 0.35 0.15 73.92

denovo1129 0.00 0.00 0.09 0.32 0.15 74.07

denovo1420 0.00 0.00 0.09 0.62 0.15 74.22

denovo293 0.00 0.00 0.09 0.25 0.15 74.37

FJ957855 0.00 0.00 0.09 0.18 0.15 74.52

JN868782 0.00 0.00 0.09 0.52 0.15 74.66

denovo1579 0.00 0.00 0.09 0.65 0.14 74.81

EU160577 0.00 0.00 0.09 0.45 0.14 74.95

denovo550 0.01 0.00 0.09 0.18 0.14 75.09

HE574926 0.00 0.00 0.09 0.44 0.14 75.22

AJ633944 0.00 0.00 0.08 0.35 0.13 75.36

denovo1491 0.00 0.00 0.08 0.21 0.13 75.49

AJ347026 0.01 0.00 0.08 0.31 0.13 75.62

FJ957708 0.00 0.00 0.08 0.18 0.13 75.75

denovo927 0.00 0.00 0.08 0.25 0.13 75.87

denovo1190 0.00 0.00 0.08 0.34 0.12 76.00

denovo1263 0.00 0.00 0.08 0.18 0.12 76.12

denovo607 0.00 0.00 0.08 0.34 0.12 76.24

denovo1182 0.01 0.00 0.08 0.33 0.12 76.37

denovo776 0.00 0.00 0.08 0.33 0.12 76.49

FJ418924 0.00 0.00 0.08 0.30 0.12 76.61

denovo40 0.00 0.00 0.07 0.23 0.12 76.73

denovo767 0.00 0.00 0.07 0.31 0.12 76.84

denovo916 0.00 0.00 0.07 0.25 0.12 76.96

FJ849072 0.00 0.00 0.07 0.18 0.12 77.08

denovo1996 0.00 0.00 0.07 0.18 0.11 77.19

denovo2003 0.00 0.00 0.07 0.35 0.11 77.31

FJ169195 0.00 0.00 0.07 0.43 0.11 77.42

denovo822 0.00 0.00 0.07 0.29 0.11 77.53

denovo1096 0.00 0.00 0.07 0.25 0.11 77.64

denovo95 0.00 0.00 0.07 0.21 0.11 77.75

denovo1341 0.00 0.00 0.07 0.31 0.11 77.86

denovo6 0.00 0.00 0.07 0.27 0.11 77.96

denovo61 0.00 0.00 0.07 0.43 0.11 78.07

denovo190 0.00 0.00 0.07 0.51 0.11 78.17

denovo1390 0.00 0.00 0.07 0.46 0.10 78.28

denovo790 0.00 0.00 0.07 0.51 0.10 78.38

GQ903461 0.00 0.00 0.06 0.28 0.10 78.48

denovo1193 0.00 0.00 0.06 0.18 0.10 78.59

GU118575 0.00 0.00 0.06 0.34 0.10 78.69

HQ671075 0.00 0.00 0.06 0.20 0.10 78.78

EU803928 0.00 0.00 0.06 0.34 0.09 78.87

denovo1789 0.00 0.00 0.06 0.34 0.09 78.97

denovo1250 0.00 0.00 0.06 0.39 0.09 79.06

GU305772 0.00 0.00 0.06 0.50 0.09 79.15

FJ826409 0.00 0.00 0.06 0.34 0.09 79.23

FJ892785 0.00 0.00 0.06 0.41 0.09 79.32

denovo1478 0.00 0.00 0.05 0.18 0.09 79.41

GU553034 0.00 0.00 0.05 0.21 0.09 79.49

FJ203135 0.00 0.00 0.05 0.25 0.09 79.58

HQ326290 0.00 0.00 0.05 0.35 0.08 79.66

denovo1921 0.00 0.00 0.05 0.20 0.08 79.75

denovo43 0.00 0.00 0.05 0.53 0.08 79.83

denovo1446 0.00 0.00 0.05 0.25 0.08 79.91

denovo499 0.00 0.00 0.05 0.26 0.08 80.00

denovo1480 0.00 0.00 0.05 0.30 0.08 80.08

denovo1570 0.00 0.00 0.05 0.18 0.08 80.16

denovo2056 0.00 0.00 0.05 0.18 0.08 80.24

denovo757 0.00 0.00 0.05 0.18 0.08 80.33

JQ032050 0.00 0.00 0.05 0.18 0.08 80.41

denovo1898 0.00 0.00 0.05 0.43 0.08 80.49

GQ433928 0.00 0.00 0.05 0.24 0.08 80.57

denovo1964 0.00 0.00 0.05 0.31 0.08 80.65

denovo2029 0.00 0.00 0.05 0.24 0.08 80.73

denovo98 0.00 0.00 0.05 0.31 0.08 80.81

denovo1284 0.00 0.00 0.05 0.32 0.08 80.88

denovo249 0.00 0.00 0.05 0.45 0.08 80.96

GU118606 0.00 0.00 0.05 0.37 0.08 81.04

GQ921403 0.00 0.00 0.05 0.18 0.08 81.11

denovo1592 0.00 0.00 0.05 0.18 0.08 81.19

denovo325 0.00 0.00 0.05 0.18 0.08 81.26

GQ263306 0.00 0.00 0.05 0.38 0.07 81.34

GU118690 0.00 0.00 0.05 0.32 0.07 81.41

denovo294 0.00 0.00 0.05 0.29 0.07 81.49

denovo696 0.00 0.00 0.05 0.18 0.07 81.56

DQ316817 0.00 0.00 0.05 0.18 0.07 81.64

denovo1757 0.00 0.00 0.05 0.29 0.07 81.71

denovo574 0.00 0.00 0.05 0.27 0.07 81.78

denovo663 0.00 0.00 0.05 0.31 0.07 81.85

denovo815 0.00 0.00 0.04 0.40 0.07 81.92

denovo777 0.00 0.00 0.04 0.39 0.07 81.99

AB294294 0.00 0.00 0.04 0.22 0.07 82.06

denovo1619 0.00 0.00 0.04 0.34 0.07 82.13

denovo7 0.00 0.00 0.04 0.45 0.07 82.19

EF076171 0.00 0.00 0.04 0.40 0.07 82.26

DQ889881 0.00 0.00 0.04 0.32 0.07 82.32

denovo1832 0.00 0.00 0.04 0.45 0.07 82.39

denovo1379 0.00 0.00 0.04 0.35 0.06 82.45

denovo676 0.00 0.00 0.04 0.41 0.06 82.52

JF925031 0.00 0.00 0.04 0.40 0.06 82.58

denovo657 0.00 0.00 0.04 0.25 0.06 82.65

denovo706 0.00 0.00 0.04 0.21 0.06 82.71

denovo729 0.00 0.00 0.04 0.36 0.06 82.77

EF613488 0.00 0.00 0.04 0.25 0.06 82.83

denovo320 0.00 0.00 0.04 0.45 0.06 82.90

FJ900573 0.00 0.00 0.04 0.32 0.06 82.96

EU463921 0.00 0.00 0.04 0.26 0.06 83.02

denovo917 0.00 0.00 0.04 0.22 0.06 83.08

denovo457 0.00 0.00 0.04 0.29 0.06 83.14

denovo520 0.00 0.00 0.04 0.28 0.06 83.20

AY959053 0.00 0.00 0.04 0.37 0.06 83.26

denovo1230 0.00 0.00 0.04 0.40 0.06 83.32

denovo1436 0.00 0.00 0.04 0.27 0.06 83.38

denovo1880 0.00 0.00 0.04 0.18 0.06 83.44

FJ557790 0.00 0.00 0.04 0.29 0.06 83.49

AJ240982 0.00 0.00 0.04 0.22 0.06 83.55

denovo172 0.00 0.00 0.04 0.35 0.06 83.61

FJ820465 0.00 0.00 0.04 0.26 0.06 83.67

denovo1423 0.00 0.00 0.04 0.38 0.06 83.72

denovo423 0.00 0.00 0.03 0.26 0.06 83.78

denovo412 0.00 0.00 0.03 0.28 0.05 83.83

denovo853 0.00 0.00 0.03 0.34 0.05 83.89

denovo528 0.00 0.00 0.03 0.20 0.05 83.94

FJ802385 0.00 0.00 0.03 0.32 0.05 83.99

FJ457296 0.00 0.00 0.03 0.22 0.05 84.05

denovo788 0.00 0.00 0.03 0.18 0.05 84.10

denovo639 0.00 0.00 0.03 0.18 0.05 84.15

FN396956 0.00 0.00 0.03 0.18 0.05 84.21

denovo305 0.00 0.00 0.03 0.31 0.05 84.26

denovo679 0.00 0.00 0.03 0.22 0.05 84.31

denovo87 0.00 0.00 0.03 0.30 0.05 84.36

denovo1500 0.00 0.00 0.03 0.18 0.05 84.42

denovo1282 0.00 0.00 0.03 0.35 0.05 84.47

denovo1694 0.00 0.00 0.03 0.35 0.05 84.52

denovo744 0.00 0.00 0.03 0.29 0.05 84.57

denovo1696 0.00 0.00 0.03 0.22 0.05 84.62

AJ621576 0.00 0.00 0.03 0.37 0.05 84.67

FJ381979 0.00 0.00 0.03 0.32 0.05 84.72

denovo659 0.00 0.00 0.03 0.35 0.05 84.76

JF712668 0.00 0.00 0.03 0.35 0.05 84.81

FJ497578 0.00 0.00 0.03 0.30 0.05 84.86

denovo1556 0.00 0.00 0.03 0.25 0.05 84.91

FJ999591 0.00 0.00 0.03 0.28 0.05 84.95

denovo1983 0.00 0.00 0.03 0.31 0.05 85.00

EF018153 0.00 0.00 0.03 0.18 0.05 85.05

HE574895 0.00 0.00 0.03 0.18 0.05 85.09

denovo193 0.00 0.00 0.03 0.31 0.05 85.14

denovo2018 0.00 0.00 0.03 0.25 0.05 85.19

denovo1590 0.00 0.00 0.03 0.31 0.04 85.23

denovo1321 0.00 0.00 0.03 0.26 0.04 85.28

HQ753432 0.00 0.00 0.03 0.30 0.04 85.32

denovo88 0.00 0.00 0.03 0.18 0.04 85.36

denovo719 0.00 0.00 0.03 0.29 0.04 85.41

denovo1199 0.00 0.00 0.03 0.18 0.04 85.45

denovo1298 0.00 0.00 0.03 0.18 0.04 85.49

denovo1830 0.00 0.00 0.03 0.18 0.04 85.54

denovo1892 0.00 0.00 0.03 0.18 0.04 85.58

denovo481 0.00 0.00 0.03 0.18 0.04 85.62

denovo694 0.00 0.00 0.03 0.18 0.04 85.67

denovo946 0.00 0.00 0.03 0.18 0.04 85.71

DQ833490 0.00 0.00 0.03 0.18 0.04 85.75

HM243844 0.00 0.00 0.03 0.18 0.04 85.80

denovo1962 0.00 0.00 0.03 0.37 0.04 85.84

denovo1701 0.00 0.00 0.03 0.36 0.04 85.88

denovo1088 0.00 0.00 0.03 0.31 0.04 85.93

denovo1459 0.00 0.00 0.03 0.36 0.04 85.97

denovo1889 0.00 0.00 0.03 0.36 0.04 86.01

denovo165 0.00 0.00 0.03 0.23 0.04 86.05

denovo1244 0.00 0.00 0.03 0.18 0.04 86.09

denovo1526 0.00 0.00 0.03 0.18 0.04 86.14

denovo1705 0.00 0.00 0.03 0.18 0.04 86.18

denovo1621 0.00 0.00 0.03 0.30 0.04 86.22

denovo1801 0.00 0.00 0.03 0.36 0.04 86.26

GU584610 0.00 0.00 0.03 0.24 0.04 86.30

denovo613 0.00 0.00 0.03 0.36 0.04 86.34

JN177591 0.00 0.00 0.03 0.24 0.04 86.38

denovo1014 0.00 0.00 0.03 0.26 0.04 86.42

denovo993 0.00 0.00 0.03 0.26 0.04 86.46

denovo1414 0.00 0.00 0.03 0.18 0.04 86.51

denovo1062 0.00 0.00 0.03 0.36 0.04 86.55

denovo611 0.00 0.00 0.03 0.18 0.04 86.59

denovo308 0.00 0.00 0.03 0.32 0.04 86.63

denovo28 0.00 0.00 0.02 0.25 0.04 86.67

DQ396132 0.00 0.00 0.02 0.24 0.04 86.71

denovo312 0.00 0.00 0.02 0.31 0.04 86.75

denovo1131 0.00 0.00 0.02 0.18 0.04 86.78

denovo143 0.00 0.00 0.02 0.23 0.04 86.82

denovo1585 0.00 0.00 0.02 0.26 0.04 86.86

denovo1093 0.00 0.00 0.02 0.18 0.04 86.90

denovo1132 0.00 0.00 0.02 0.18 0.04 86.94

denovo860 0.00 0.00 0.02 0.18 0.04 86.98

denovo1637 0.00 0.00 0.02 0.31 0.04 87.02

EF076172 0.00 0.00 0.02 0.31 0.04 87.05

denovo473 0.00 0.00 0.02 0.18 0.04 87.09

denovo1581 0.00 0.00 0.02 0.31 0.04 87.13

FJ529262 0.00 0.00 0.02 0.30 0.04 87.17

denovo103 0.00 0.00 0.02 0.18 0.04 87.21

denovo812 0.00 0.00 0.02 0.29 0.04 87.24

denovo1688 0.00 0.00 0.02 0.18 0.04 87.28

EF153415 0.00 0.00 0.02 0.31 0.04 87.32

AY922243 0.00 0.00 0.02 0.25 0.04 87.35

GU584786 0.00 0.00 0.02 0.32 0.04 87.39

denovo1303 0.00 0.00 0.02 0.24 0.04 87.43

denovo1670 0.00 0.00 0.02 0.25 0.04 87.46

denovo707 0.00 0.00 0.02 0.26 0.04 87.50

denovo1338 0.00 0.00 0.02 0.18 0.04 87.53

denovo1291 0.00 0.00 0.02 0.24 0.04 87.57

HQ672216 0.00 0.00 0.02 0.25 0.04 87.61

denovo1555 0.00 0.00 0.02 0.24 0.04 87.64

HQ588359 0.00 0.00 0.02 0.18 0.04 87.68

HQ118340 0.00 0.00 0.02 0.30 0.04 87.71

AJ867671 0.00 0.00 0.02 0.32 0.04 87.75

EU817491 0.00 0.00 0.02 0.25 0.04 87.78

FQ660217 0.00 0.00 0.02 0.26 0.03 87.82

denovo690 0.00 0.00 0.02 0.26 0.03 87.85

GQ274045 0.00 0.00 0.02 0.26 0.03 87.89

denovo521 0.00 0.00 0.02 0.18 0.03 87.92

denovo1944 0.00 0.00 0.02 0.31 0.03 87.95

HQ616309 0.00 0.00 0.02 0.25 0.03 87.99

AJ583166 0.00 0.00 0.02 0.22 0.03 88.02

denovo1899 0.00 0.00 0.02 0.24 0.03 88.06

denovo1118 0.00 0.00 0.02 0.25 0.03 88.09

denovo692 0.00 0.00 0.02 0.31 0.03 88.12

denovo990 0.00 0.00 0.02 0.18 0.03 88.16

JF808978 0.00 0.00 0.02 0.32 0.03 88.19

denovo1561 0.00 0.00 0.02 0.24 0.03 88.22

AB176169 0.00 0.00 0.02 0.22 0.03 88.26

denovo492 0.00 0.00 0.02 0.30 0.03 88.29

denovo1641 0.00 0.00 0.02 0.18 0.03 88.32

denovo1517 0.00 0.00 0.02 0.25 0.03 88.35

denovo1403 0.00 0.00 0.02 0.31 0.03 88.39

denovo272 0.00 0.00 0.02 0.25 0.03 88.42

denovo1823 0.00 0.00 0.02 0.32 0.03 88.45

denovo1452 0.00 0.00 0.02 0.32 0.03 88.48

DQ889935 0.00 0.00 0.02 0.26 0.03 88.51

FJ957596 0.00 0.00 0.02 0.30 0.03 88.55

denovo1147 0.00 0.00 0.02 0.22 0.03 88.58

JN411267 0.00 0.00 0.02 0.25 0.03 88.61

FJ808721 0.00 0.00 0.02 0.32 0.03 88.64

denovo1082 0.00 0.00 0.02 0.18 0.03 88.67

denovo1690 0.00 0.00 0.02 0.25 0.03 88.70

denovo1754 0.00 0.00 0.02 0.25 0.03 88.74

denovo1827 0.00 0.00 0.02 0.32 0.03 88.77

denovo771 0.00 0.00 0.02 0.25 0.03 88.80

denovo60 0.00 0.00 0.02 0.25 0.03 88.83

denovo850 0.00 0.00 0.02 0.31 0.03 88.86

denovo1875 0.00 0.00 0.02 0.18 0.03 88.89

denovo24 0.00 0.00 0.02 0.25 0.03 88.92

denovo183 0.00 0.00 0.02 0.18 0.03 88.95

denovo1346 0.00 0.00 0.02 0.31 0.03 88.98

denovo608 0.00 0.00 0.02 0.26 0.03 89.01

AJ863291 0.00 0.00 0.02 0.18 0.03 89.04

denovo1274 0.00 0.00 0.02 0.18 0.03 89.07

denovo184 0.00 0.00 0.02 0.31 0.03 89.10

denovo887 0.00 0.00 0.02 0.26 0.03 89.13

denovo34 0.00 0.00 0.02 0.18 0.03 89.16

denovo1401 0.00 0.00 0.02 0.30 0.03 89.19

denovo210 0.00 0.00 0.02 0.25 0.03 89.22

denovo148 0.00 0.00 0.02 0.18 0.03 89.25

HQ326257 0.00 0.00 0.02 0.18 0.03 89.28

denovo872 0.00 0.00 0.02 0.25 0.03 89.30

denovo682 0.00 0.00 0.02 0.25 0.03 89.33

denovo1917 0.00 0.00 0.02 0.25 0.03 89.36

denovo898 0.00 0.00 0.02 0.26 0.03 89.39

denovo1265 0.00 0.00 0.02 0.26 0.03 89.42

denovo871 0.00 0.00 0.02 0.24 0.03 89.45

denovo1095 0.00 0.00 0.02 0.18 0.03 89.48

denovo1759 0.00 0.00 0.02 0.31 0.03 89.51

denovo1240 0.00 0.00 0.02 0.25 0.03 89.53

denovo1489 0.00 0.00 0.02 0.26 0.03 89.56

denovo1655 0.00 0.00 0.02 0.26 0.03 89.59

denovo1103 0.00 0.00 0.02 0.24 0.03 89.62

denovo818 0.00 0.00 0.02 0.18 0.03 89.64

denovo147 0.00 0.00 0.02 0.18 0.03 89.67

denovo199 0.00 0.00 0.02 0.18 0.03 89.70

EU539909 0.00 0.00 0.02 0.18 0.03 89.72

denovo1203 0.00 0.00 0.02 0.26 0.03 89.75

denovo636 0.00 0.00 0.02 0.24 0.03 89.78

denovo1441 0.00 0.00 0.02 0.32 0.03 89.81

denovo1523 0.00 0.00 0.02 0.18 0.03 89.83

denovo1839 0.00 0.00 0.02 0.18 0.03 89.86

denovo543 0.00 0.00 0.02 0.25 0.03 89.89

denovo1150 0.00 0.00 0.02 0.18 0.03 89.91

denovo1959 0.00 0.00 0.02 0.25 0.03 89.94

denovo1383 0.00 0.00 0.02 0.18 0.03 89.97

denovo225 0.00 0.00 0.02 0.18 0.03 89.99

denovo727 0.00 0.00 0.02 0.26 0.03 90.02

**(D)**

Groups Aplysina & Corticium

Average dissimilarity = 73.50

Group Aplysina Group Corticium

Species Av.Abund Av.Abund Av.Diss Diss/SD Contrib% Cum.%

denovo1749 0.77 0.63 7.91 0.94 10.77 10.77

denovo1026 0.16 0.15 2.85 1.03 3.88 14.64

JN579972 0.00 0.13 2.71 0.42 3.69 18.34

HE574879 0.13 0.00 2.47 1.13 3.36 21.70

GQ118701 0.02 0.12 2.41 0.74 3.28 24.98

denovo75 0.00 0.12 2.09 0.39 2.84 27.83

HM595366 0.10 0.00 1.70 0.54 2.31 30.13

denovo1888 0.09 0.00 1.54 0.54 2.09 32.23

FJ516885 0.00 0.06 1.50 0.25 2.04 34.27

FJ624884 0.08 0.00 1.27 0.44 1.73 36.00

denovo552 0.05 0.00 1.07 1.00 1.46 37.46

U81990 0.06 0.00 1.03 0.53 1.40 38.86

denovo427 0.05 0.01 1.01 1.15 1.37 40.23

denovo547 0.04 0.00 0.91 1.26 1.24 41.48

denovo1484 0.03 0.14 0.91 0.81 1.24 42.72

JF937422 0.03 0.13 0.86 0.76 1.17 43.88

JN874385 0.04 0.00 0.85 0.95 1.16 45.04

FJ751910 0.00 0.04 0.69 0.23 0.94 45.98

AM709702 0.04 0.00 0.69 0.41 0.94 46.92

denovo1017 0.00 0.16 0.67 0.44 0.92 47.83

JF802167 0.00 0.03 0.64 0.26 0.88 48.71

denovo605 0.04 0.00 0.64 0.45 0.86 49.58

denovo119 0.03 0.00 0.62 1.18 0.84 50.42

denovo189 0.00 0.03 0.59 0.77 0.81 51.22

denovo1146 0.00 0.03 0.56 0.77 0.76 51.98

AY739689 0.00 0.03 0.53 0.23 0.72 52.71

HQ245988 0.00 0.03 0.52 0.25 0.70 53.41

denovo1479 0.02 0.00 0.47 0.67 0.63 54.04

denovo953 0.02 0.00 0.46 0.99 0.63 54.67

denovo862 0.03 0.00 0.45 0.41 0.61 55.28

EU846601 0.00 0.03 0.44 0.23 0.60 55.88

AF286480 0.00 0.02 0.42 0.70 0.57 56.45

denovo114 0.00 0.11 0.42 0.37 0.57 57.02

denovo109 0.00 0.02 0.42 0.72 0.57 57.60

denovo1070 0.02 0.00 0.42 1.46 0.57 58.16

AB299573 0.03 0.00 0.40 0.23 0.54 58.71

denovo507 0.00 0.02 0.39 0.71 0.53 59.24

denovo756 0.00 0.11 0.38 0.34 0.51 59.75

denovo1249 0.00 0.02 0.37 0.84 0.51 60.26

denovo838 0.00 0.11 0.36 0.33 0.49 60.75

denovo1558 0.02 0.00 0.36 0.43 0.49 61.23

denovo1189 0.00 0.02 0.36 0.71 0.49 61.72

denovo1905 0.00 0.15 0.36 0.23 0.49 62.21

denovo1961 0.02 0.00 0.35 0.49 0.48 62.69

denovo1873 0.00 0.02 0.35 0.40 0.48 63.17

denovo1791 0.00 0.11 0.31 0.28 0.42 63.59

denovo204 0.00 0.01 0.30 0.85 0.41 64.00

denovo1243 0.02 0.00 0.30 0.43 0.41 64.41

GU940713 0.01 0.01 0.30 0.55 0.41 64.82

denovo1022 0.00 0.02 0.30 0.48 0.40 65.22

denovo1476 0.00 0.11 0.29 0.27 0.40 65.62

denovo1743 0.00 0.11 0.29 0.27 0.40 66.02

denovo324 0.00 0.11 0.29 0.27 0.40 66.41

denovo1462 0.01 0.00 0.28 1.00 0.38 66.80

EU536078 0.00 0.01 0.28 0.70 0.38 67.17

AB176201 0.00 0.01 0.28 0.56 0.38 67.55

denovo1085 0.01 0.00 0.27 1.02 0.37 67.92

denovo1322 0.00 0.11 0.27 0.24 0.36 68.29

denovo1842 0.01 0.00 0.26 1.06 0.36 68.64

AY631057 0.00 0.02 0.26 0.27 0.35 69.00

HQ118426 0.02 0.00 0.26 0.43 0.35 69.35

denovo1273 0.01 0.01 0.26 0.63 0.35 69.69

denovo1565 0.00 0.11 0.25 0.23 0.34 70.04

denovo1640 0.00 0.11 0.25 0.23 0.34 70.38

denovo1785 0.00 0.11 0.25 0.23 0.34 70.72

GQ385289 0.00 0.11 0.25 0.23 0.34 71.07

AY914065 0.01 0.01 0.25 0.77 0.34 71.41

AM992178 0.00 0.01 0.24 0.24 0.33 71.74

AJ292596 0.01 0.00 0.23 0.41 0.32 72.05

denovo792 0.00 0.01 0.23 0.40 0.31 72.37

EU935300 0.01 0.01 0.22 0.66 0.30 72.67

denovo1720 0.01 0.00 0.21 0.45 0.29 72.96

denovo1292 0.01 0.00 0.21 0.60 0.28 73.24

denovo545 0.01 0.01 0.20 1.21 0.27 73.52

DQ860060 0.01 0.00 0.20 0.79 0.27 73.79

denovo141 0.00 0.01 0.18 0.67 0.25 74.03

denovo709 0.01 0.00 0.18 0.87 0.25 74.28

denovo1703 0.01 0.00 0.17 0.45 0.23 74.51

denovo415 0.01 0.00 0.17 0.92 0.22 74.74

HQ323455 0.00 0.01 0.16 0.25 0.22 74.96

denovo1923 0.01 0.00 0.16 0.53 0.22 75.18

denovo198 0.01 0.00 0.16 0.35 0.22 75.40

denovo1753 0.00 0.01 0.16 0.34 0.22 75.62

denovo1577 0.00 0.01 0.16 0.57 0.21 75.83

JF802166 0.00 0.01 0.15 0.23 0.21 76.04

denovo1420 0.01 0.00 0.15 0.88 0.21 76.24

denovo854 0.00 0.01 0.15 0.55 0.21 76.45

denovo1412 0.01 0.00 0.15 0.36 0.20 76.65

AF252321 0.00 0.01 0.15 0.28 0.20 76.85

denovo2019 0.01 0.00 0.15 0.45 0.20 77.05

JF937433 0.01 0.00 0.15 0.52 0.20 77.25

denovo1129 0.01 0.00 0.14 0.41 0.20 77.45

denovo1306 0.00 0.01 0.14 0.32 0.19 77.64

HE574926 0.01 0.00 0.14 0.59 0.19 77.82

FJ957855 0.00 0.01 0.13 0.23 0.18 78.01

HQ721418 0.01 0.00 0.13 0.61 0.18 78.19

AJ633944 0.01 0.00 0.13 0.45 0.18 78.36

denovo1145 0.00 0.01 0.13 0.69 0.17 78.54

X97093 0.01 0.00 0.13 0.57 0.17 78.71

HM108471 0.00 0.00 0.13 0.56 0.17 78.88

HQ616267 0.00 0.00 0.13 0.61 0.17 79.06

denovo550 0.00 0.01 0.13 0.23 0.17 79.23

denovo293 0.00 0.00 0.12 0.28 0.17 79.40

denovo1190 0.01 0.00 0.12 0.44 0.16 79.56

denovo1491 0.00 0.01 0.12 0.26 0.16 79.73

denovo927 0.01 0.00 0.12 0.32 0.16 79.88

denovo607 0.00 0.01 0.11 0.44 0.16 80.04

FJ957708 0.00 0.01 0.11 0.23 0.16 80.20

denovo767 0.01 0.00 0.11 0.40 0.15 80.35

denovo1263 0.00 0.01 0.11 0.23 0.15 80.50

denovo190 0.01 0.00 0.11 0.72 0.15 80.65

denovo790 0.00 0.00 0.11 0.72 0.15 80.80

denovo40 0.00 0.01 0.11 0.30 0.15 80.94

denovo916 0.00 0.00 0.11 0.32 0.15 81.09

FJ849072 0.00 0.01 0.11 0.23 0.14 81.23

denovo822 0.01 0.00 0.10 0.36 0.14 81.38

denovo1996 0.00 0.01 0.10 0.23 0.14 81.52

denovo1096 0.01 0.00 0.10 0.31 0.14 81.66

denovo1341 0.01 0.00 0.10 0.39 0.14 81.80

denovo95 0.01 0.00 0.10 0.26 0.14 81.94

denovo1632 0.00 0.00 0.10 0.51 0.13 82.07

JN868782 0.00 0.00 0.10 0.55 0.13 82.20

denovo430 0.00 0.00 0.10 0.64 0.13 82.33

GQ903461 0.00 0.01 0.09 0.35 0.13 82.46

denovo1193 0.00 0.00 0.09 0.23 0.12 82.58

EU035954 0.00 0.01 0.09 0.49 0.12 82.71

HQ671075 0.00 0.00 0.09 0.25 0.12 82.82

denovo1390 0.00 0.00 0.08 0.51 0.12 82.94

AF170746 0.00 0.00 0.08 0.25 0.11 83.05

denovo1480 0.00 0.00 0.08 0.38 0.11 83.16

denovo1478 0.00 0.00 0.08 0.23 0.11 83.27

denovo1446 0.00 0.00 0.08 0.32 0.11 83.37

denovo1921 0.00 0.00 0.08 0.25 0.11 83.48

AB477015 0.00 0.00 0.08 0.44 0.11 83.59

denovo1964 0.00 0.00 0.08 0.40 0.10 83.69

denovo1570 0.00 0.00 0.07 0.23 0.10 83.79

denovo2056 0.00 0.00 0.07 0.23 0.10 83.89

denovo757 0.00 0.00 0.07 0.23 0.10 83.99

JQ032050 0.00 0.00 0.07 0.23 0.10 84.10

denovo499 0.00 0.00 0.07 0.33 0.10 84.20

denovo1579 0.00 0.00 0.07 0.51 0.10 84.30

denovo2029 0.00 0.00 0.07 0.31 0.10 84.40

denovo1898 0.00 0.00 0.07 0.56 0.10 84.50

FJ418924 0.00 0.00 0.07 0.27 0.10 84.60

denovo294 0.00 0.00 0.07 0.36 0.10 84.70

denovo828 0.00 0.00 0.07 0.32 0.10 84.79

GU553034 0.00 0.00 0.07 0.23 0.09 84.89

denovo663 0.00 0.00 0.07 0.40 0.09 84.98

DQ316817 0.00 0.00 0.07 0.22 0.09 85.08

denovo1592 0.00 0.00 0.07 0.23 0.09 85.17

denovo325 0.00 0.00 0.07 0.23 0.09 85.26

GU179554 0.00 0.00 0.07 0.40 0.09 85.36

denovo696 0.00 0.00 0.07 0.23 0.09 85.45

GQ921403 0.00 0.00 0.07 0.22 0.09 85.54

denovo7 0.00 0.00 0.07 0.61 0.09 85.63

denovo1832 0.00 0.00 0.07 0.60 0.09 85.72

FJ203135 0.00 0.00 0.06 0.25 0.09 85.81

denovo1379 0.00 0.00 0.06 0.46 0.09 85.89

denovo1757 0.00 0.00 0.06 0.34 0.08 85.98

denovo729 0.00 0.00 0.06 0.47 0.08 86.06

denovo657 0.00 0.00 0.06 0.32 0.08 86.14

AB294294 0.00 0.00 0.06 0.28 0.08 86.23

EF613488 0.00 0.00 0.06 0.32 0.08 86.31

denovo43 0.00 0.00 0.06 0.58 0.08 86.39

denovo706 0.00 0.00 0.06 0.26 0.08 86.47

denovo1230 0.00 0.00 0.06 0.53 0.08 86.55

EU463921 0.00 0.00 0.06 0.32 0.08 86.63

FJ826409 0.00 0.00 0.06 0.32 0.08 86.70

denovo520 0.00 0.00 0.06 0.36 0.08 86.78

denovo1436 0.00 0.00 0.06 0.33 0.08 86.86

denovo172 0.00 0.00 0.06 0.45 0.08 86.93

denovo1880 0.00 0.00 0.05 0.22 0.07 87.01

denovo676 0.00 0.00 0.05 0.47 0.07 87.08

denovo1423 0.00 0.00 0.05 0.48 0.07 87.15

denovo1374 0.00 0.00 0.05 0.30 0.07 87.22

EU160577 0.00 0.00 0.05 0.34 0.07 87.29

denovo412 0.00 0.00 0.05 0.36 0.07 87.36

GU305772 0.00 0.00 0.05 0.51 0.07 87.43

FJ457296 0.00 0.00 0.05 0.27 0.07 87.49

denovo1282 0.00 0.00 0.05 0.45 0.07 87.56

denovo639 0.00 0.00 0.05 0.22 0.07 87.63

FN396956 0.00 0.00 0.05 0.22 0.07 87.69

denovo1694 0.00 0.00 0.05 0.45 0.07 87.76

denovo2003 0.00 0.00 0.05 0.22 0.06 87.82

denovo1500 0.00 0.00 0.05 0.22 0.06 87.89

denovo679 0.00 0.00 0.05 0.27 0.06 87.95

denovo87 0.00 0.00 0.05 0.36 0.06 88.02

denovo744 0.00 0.00 0.05 0.38 0.06 88.08

denovo1696 0.00 0.00 0.05 0.28 0.06 88.14

denovo1983 0.00 0.00 0.04 0.39 0.06 88.20

JF712668 0.00 0.00 0.04 0.46 0.06 88.26

HQ326290 0.00 0.00 0.04 0.38 0.06 88.32

EF018153 0.00 0.00 0.04 0.22 0.06 88.38

HE574895 0.00 0.00 0.04 0.22 0.06 88.44

denovo2018 0.00 0.00 0.04 0.32 0.06 88.50

denovo1321 0.00 0.00 0.04 0.32 0.06 88.56

denovo1459 0.00 0.00 0.04 0.47 0.06 88.61

FJ557790 0.00 0.00 0.04 0.30 0.06 88.67

denovo777 0.00 0.00 0.04 0.37 0.06 88.73

denovo1088 0.00 0.00 0.04 0.39 0.06 88.78

denovo719 0.00 0.00 0.04 0.37 0.05 88.84

denovo88 0.00 0.00 0.04 0.23 0.05 88.89

denovo1199 0.00 0.00 0.04 0.23 0.05 88.95

denovo1298 0.00 0.00 0.04 0.23 0.05 89.00

denovo1830 0.00 0.00 0.04 0.23 0.05 89.05

denovo1892 0.00 0.00 0.04 0.23 0.05 89.11

denovo481 0.00 0.00 0.04 0.23 0.05 89.16

denovo694 0.00 0.00 0.04 0.23 0.05 89.21

denovo946 0.00 0.00 0.04 0.23 0.05 89.27

DQ833490 0.00 0.00 0.04 0.23 0.05 89.32

HM243844 0.00 0.00 0.04 0.23 0.05 89.37

JN177591 0.00 0.00 0.04 0.30 0.05 89.43

GU584610 0.00 0.00 0.04 0.30 0.05 89.48

denovo1014 0.00 0.00 0.04 0.32 0.05 89.53

denovo993 0.00 0.00 0.04 0.32 0.05 89.59

denovo320 0.00 0.00 0.04 0.44 0.05 89.64

denovo6 0.00 0.00 0.04 0.33 0.05 89.69

denovo312 0.00 0.00 0.04 0.39 0.05 89.74

denovo1889 0.00 0.00 0.04 0.46 0.05 89.80

denovo1414 0.00 0.00 0.04 0.22 0.05 89.85

denovo1705 0.00 0.00 0.04 0.23 0.05 89.90

denovo1526 0.00 0.00 0.04 0.22 0.05 89.95

denovo611 0.00 0.00 0.04 0.23 0.05 90.00

denovo1585 0.00 0.00 0.04 0.32 0.05 90.05

Groups Aplysina & Petrosia

Average dissimilarity = 60.95

Group Aplysina Group Petrosia

Species Av.Abund Av.Abund Av.Diss Diss/SD Contrib% Cum.%

denovo1749 0.77 0.89 4.68 0.79 7.68 7.68

denovo1026 0.16 0.01 2.96 0.95 4.86 12.53

HE574879 0.13 0.00 2.68 1.14 4.39 16.93

GQ118701 0.02 0.10 2.16 0.97 3.55 20.47

HM595366 0.10 0.00 1.82 0.54 2.98 23.46

denovo1888 0.09 0.00 1.65 0.55 2.71 26.17

FJ624884 0.08 0.00 1.36 0.44 2.22 28.39

denovo552 0.05 0.00 1.17 1.02 1.93 30.32

U81990 0.06 0.00 1.11 0.53 1.83 32.15

JN030555 0.00 0.05 1.11 0.24 1.81 33.96

denovo427 0.05 0.01 1.09 1.15 1.78 35.74

denovo547 0.04 0.00 1.02 1.30 1.67 37.41

JN874385 0.04 0.00 0.94 0.96 1.53 38.95

JF937422 0.03 0.06 0.93 0.86 1.52 40.47

AM709702 0.04 0.01 0.78 0.44 1.28 41.74

denovo119 0.03 0.00 0.68 1.20 1.11 42.86

denovo605 0.04 0.00 0.67 0.44 1.10 43.96

denovo1484 0.03 0.02 0.67 1.24 1.10 45.06

denovo1146 0.00 0.03 0.64 1.04 1.05 46.11

denovo1479 0.02 0.00 0.51 0.71 0.84 46.95

denovo953 0.02 0.00 0.51 1.01 0.83 47.78

denovo862 0.03 0.00 0.50 0.43 0.81 48.60

denovo189 0.00 0.02 0.49 0.90 0.81 49.41

denovo1070 0.02 0.00 0.46 1.48 0.75 50.16

denovo1249 0.00 0.02 0.43 1.12 0.71 50.87

AY914065 0.01 0.03 0.43 0.76 0.70 51.57

AB299573 0.03 0.00 0.43 0.23 0.70 52.27

denovo1558 0.02 0.00 0.40 0.43 0.65 52.92

denovo1189 0.00 0.02 0.38 0.92 0.63 53.55

denovo1961 0.02 0.00 0.38 0.49 0.63 54.17

denovo507 0.00 0.02 0.35 0.89 0.57 54.74

EU935300 0.01 0.02 0.34 0.76 0.57 55.31

denovo109 0.00 0.01 0.34 0.90 0.55 55.86

denovo1243 0.02 0.00 0.33 0.43 0.53 56.40

denovo1462 0.01 0.00 0.31 1.02 0.51 56.90

denovo828 0.00 0.01 0.30 0.46 0.49 57.39

denovo1085 0.01 0.00 0.29 1.05 0.48 57.88

DQ889875 0.00 0.02 0.29 0.48 0.47 58.35

denovo1842 0.01 0.00 0.29 1.06 0.47 58.82

HQ118426 0.02 0.00 0.28 0.45 0.46 59.29

denovo1720 0.01 0.00 0.26 0.52 0.43 59.71

AJ292596 0.01 0.00 0.25 0.41 0.41 60.12

EU035954 0.00 0.02 0.25 0.40 0.41 60.53

denovo1292 0.01 0.00 0.23 0.63 0.38 60.91

AM935808 0.00 0.02 0.23 0.23 0.37 61.28

denovo545 0.01 0.01 0.22 1.31 0.37 61.65

denovo1273 0.01 0.01 0.21 0.72 0.35 61.99

GU118526 0.00 0.02 0.21 0.36 0.34 62.34

AB176201 0.00 0.01 0.21 0.65 0.34 62.68

denovo415 0.01 0.01 0.20 1.11 0.34 63.01

DQ860060 0.01 0.00 0.20 0.74 0.33 63.35

denovo141 0.00 0.01 0.20 0.73 0.33 63.68

denovo709 0.01 0.00 0.20 0.90 0.32 64.00

GU940713 0.01 0.01 0.19 0.65 0.31 64.31

GU179554 0.00 0.01 0.19 0.71 0.31 64.62

denovo1145 0.00 0.01 0.19 0.94 0.30 64.92

FQ659744 0.00 0.01 0.19 0.22 0.30 65.23

denovo1848 0.00 0.01 0.18 0.23 0.30 65.53

denovo1703 0.01 0.00 0.18 0.46 0.30 65.83

HQ721418 0.01 0.01 0.18 0.65 0.29 66.12

denovo1547 0.00 0.02 0.18 0.44 0.29 66.41

denovo1923 0.01 0.00 0.18 0.54 0.29 66.70

X97093 0.01 0.01 0.17 0.69 0.29 66.99

denovo198 0.01 0.00 0.17 0.32 0.27 67.26

denovo838 0.00 0.01 0.17 1.17 0.27 67.53

denovo1162 0.00 0.01 0.17 0.43 0.27 67.80

denovo1420 0.01 0.00 0.16 0.91 0.27 68.07

denovo1412 0.01 0.00 0.16 0.36 0.26 68.34

denovo2019 0.01 0.00 0.16 0.45 0.26 68.60

denovo1129 0.01 0.00 0.16 0.41 0.26 68.85

AB477015 0.00 0.01 0.15 0.63 0.25 69.10

denovo1632 0.00 0.00 0.15 0.75 0.25 69.35

HE574926 0.01 0.00 0.15 0.59 0.25 69.60

denovo430 0.00 0.01 0.15 0.83 0.25 69.84

HQ616267 0.00 0.01 0.15 0.81 0.24 70.08

denovo1017 0.00 0.01 0.14 0.53 0.23 70.32

AJ633944 0.01 0.00 0.14 0.46 0.23 70.55

denovo204 0.00 0.00 0.13 0.61 0.22 70.77

denovo1190 0.01 0.00 0.13 0.44 0.21 70.98

denovo927 0.01 0.00 0.13 0.32 0.21 71.19

HM108471 0.00 0.01 0.12 0.46 0.21 71.39

denovo854 0.00 0.01 0.12 0.32 0.20 71.59

AJ347026 0.00 0.01 0.12 0.39 0.20 71.79

denovo767 0.01 0.00 0.12 0.40 0.20 71.99

denovo190 0.01 0.00 0.12 0.72 0.20 72.19

denovo790 0.00 0.00 0.12 0.73 0.19 72.38

JF937433 0.01 0.00 0.12 0.40 0.19 72.58

AF170746 0.00 0.01 0.12 0.45 0.19 72.77

EU160577 0.00 0.01 0.12 0.49 0.19 72.96

denovo1182 0.00 0.01 0.11 0.43 0.19 73.14

denovo776 0.00 0.01 0.11 0.43 0.19 73.33

denovo822 0.01 0.00 0.11 0.36 0.18 73.52

denovo1579 0.00 0.00 0.11 0.76 0.18 73.70

denovo1096 0.01 0.00 0.11 0.31 0.18 73.88

denovo95 0.01 0.00 0.11 0.27 0.18 74.06

denovo1341 0.01 0.00 0.11 0.39 0.18 74.25

denovo2003 0.00 0.01 0.11 0.43 0.18 74.42

FJ169195 0.00 0.00 0.11 0.57 0.18 74.60

denovo1022 0.00 0.01 0.11 0.41 0.17 74.77

denovo1374 0.00 0.00 0.11 0.29 0.17 74.95

denovo1390 0.00 0.00 0.10 0.58 0.17 75.11

HQ671075 0.00 0.00 0.10 0.25 0.16 75.27

GU118575 0.00 0.01 0.10 0.44 0.16 75.43

denovo1480 0.00 0.00 0.09 0.38 0.14 75.57

denovo1789 0.00 0.00 0.09 0.44 0.14 75.71

denovo1446 0.00 0.00 0.08 0.32 0.14 75.85

denovo1921 0.00 0.00 0.08 0.25 0.14 75.99

denovo1964 0.00 0.00 0.08 0.40 0.14 76.12

denovo294 0.00 0.00 0.08 0.36 0.13 76.25

denovo2029 0.00 0.00 0.08 0.31 0.13 76.39

denovo61 0.00 0.01 0.08 0.45 0.13 76.51

FJ203135 0.00 0.00 0.07 0.27 0.12 76.63

denovo1898 0.00 0.00 0.07 0.52 0.12 76.76

denovo663 0.00 0.00 0.07 0.40 0.12 76.88

EU803928 0.00 0.01 0.07 0.38 0.12 77.00

denovo98 0.00 0.01 0.07 0.40 0.12 77.12

DQ316817 0.00 0.00 0.07 0.22 0.12 77.24

denovo7 0.00 0.00 0.07 0.61 0.12 77.36

denovo1284 0.00 0.01 0.07 0.40 0.12 77.48

denovo1832 0.00 0.00 0.07 0.60 0.12 77.60

GU118606 0.00 0.01 0.07 0.48 0.12 77.72

GQ433928 0.00 0.00 0.07 0.31 0.12 77.84

GU118690 0.00 0.01 0.07 0.41 0.12 77.96

FJ892785 0.00 0.00 0.07 0.46 0.11 78.07

denovo1379 0.00 0.00 0.07 0.46 0.11 78.18

denovo574 0.00 0.01 0.07 0.35 0.11 78.29

denovo729 0.00 0.00 0.07 0.47 0.11 78.40

EF613488 0.00 0.00 0.07 0.32 0.11 78.51

denovo657 0.00 0.00 0.07 0.32 0.11 78.62

denovo1743 0.00 0.00 0.07 0.51 0.11 78.73

denovo1230 0.00 0.00 0.06 0.54 0.11 78.83

denovo676 0.00 0.00 0.06 0.52 0.11 78.94

denovo706 0.00 0.00 0.06 0.27 0.10 79.04

DQ889881 0.00 0.01 0.06 0.41 0.10 79.15

denovo520 0.00 0.00 0.06 0.36 0.10 79.25

EU463921 0.00 0.00 0.06 0.33 0.10 79.35

denovo815 0.00 0.00 0.06 0.50 0.10 79.45

HQ326290 0.00 0.00 0.06 0.38 0.10 79.55

denovo172 0.00 0.00 0.06 0.46 0.10 79.65

denovo1880 0.00 0.00 0.06 0.22 0.10 79.75

denovo1436 0.00 0.00 0.06 0.34 0.10 79.85

GU305772 0.00 0.00 0.06 0.47 0.10 79.95

denovo249 0.00 0.00 0.06 0.55 0.10 80.05

denovo6 0.00 0.00 0.06 0.22 0.10 80.15

JN868782 0.00 0.00 0.06 0.41 0.10 80.24

FJ900573 0.00 0.01 0.06 0.41 0.10 80.34

AY959053 0.00 0.00 0.06 0.47 0.09 80.43

denovo1423 0.00 0.00 0.06 0.48 0.09 80.52

denovo917 0.00 0.00 0.05 0.27 0.09 80.61

FJ457296 0.00 0.00 0.05 0.28 0.09 80.70

denovo1282 0.00 0.00 0.05 0.45 0.09 80.79

denovo756 0.00 0.00 0.05 0.47 0.09 80.88

denovo639 0.00 0.00 0.05 0.22 0.09 80.96

FN396956 0.00 0.00 0.05 0.22 0.09 81.05

denovo1694 0.00 0.00 0.05 0.45 0.09 81.14

AJ240982 0.00 0.00 0.05 0.28 0.09 81.22

denovo1250 0.00 0.00 0.05 0.34 0.09 81.31

FJ418924 0.00 0.00 0.05 0.40 0.09 81.40

denovo423 0.00 0.00 0.05 0.32 0.09 81.48

denovo457 0.00 0.00 0.05 0.37 0.09 81.57

denovo1500 0.00 0.00 0.05 0.22 0.08 81.65

FJ802385 0.00 0.00 0.05 0.40 0.08 81.74

denovo528 0.00 0.00 0.05 0.26 0.08 81.82

denovo305 0.00 0.00 0.05 0.40 0.08 81.90

GQ263306 0.00 0.00 0.05 0.40 0.08 81.98

denovo788 0.00 0.00 0.05 0.23 0.08 82.06

denovo1983 0.00 0.00 0.05 0.40 0.08 82.14

FJ381979 0.00 0.00 0.05 0.40 0.08 82.22

EF018153 0.00 0.00 0.05 0.22 0.08 82.30

HE574895 0.00 0.00 0.05 0.22 0.08 82.37

denovo2018 0.00 0.00 0.05 0.32 0.08 82.45

denovo1088 0.00 0.00 0.05 0.40 0.08 82.52

denovo1321 0.00 0.00 0.05 0.32 0.08 82.60

denovo1459 0.00 0.00 0.05 0.48 0.07 82.67

denovo1556 0.00 0.00 0.05 0.30 0.07 82.75

denovo193 0.00 0.00 0.04 0.39 0.07 82.82

JN177591 0.00 0.00 0.04 0.30 0.07 82.89

FJ497578 0.00 0.00 0.04 0.38 0.07 82.96

denovo324 0.00 0.00 0.04 0.40 0.07 83.03

denovo1590 0.00 0.00 0.04 0.40 0.07 83.10

denovo320 0.00 0.00 0.04 0.44 0.07 83.17

denovo87 0.00 0.00 0.04 0.31 0.07 83.24

denovo312 0.00 0.00 0.04 0.39 0.07 83.31

GU584610 0.00 0.00 0.04 0.30 0.07 83.38

denovo1014 0.00 0.00 0.04 0.32 0.07 83.45

denovo993 0.00 0.00 0.04 0.32 0.07 83.52

HQ753432 0.00 0.00 0.04 0.37 0.07 83.59

denovo1414 0.00 0.00 0.04 0.22 0.07 83.65

JF925031 0.00 0.00 0.04 0.49 0.07 83.72

denovo1585 0.00 0.00 0.04 0.32 0.06 83.78

denovo165 0.00 0.00 0.04 0.29 0.06 83.85

denovo1621 0.00 0.00 0.04 0.37 0.06 83.91

denovo1132 0.00 0.00 0.04 0.22 0.06 83.97

denovo473 0.00 0.00 0.04 0.22 0.06 84.04

GU584786 0.00 0.00 0.04 0.41 0.06 84.10

denovo916 0.00 0.00 0.04 0.32 0.06 84.16

denovo103 0.00 0.00 0.04 0.22 0.06 84.22

denovo1801 0.00 0.00 0.04 0.45 0.06 84.28

denovo1244 0.00 0.00 0.04 0.22 0.06 84.35

denovo1670 0.00 0.00 0.04 0.31 0.06 84.41

AY922243 0.00 0.00 0.04 0.31 0.06 84.47

denovo1338 0.00 0.00 0.04 0.22 0.06 84.53

EF076172 0.00 0.00 0.04 0.40 0.06 84.59

FJ529262 0.00 0.00 0.04 0.38 0.06 84.65

denovo1619 0.00 0.00 0.04 0.29 0.06 84.70

DQ396132 0.00 0.00 0.04 0.31 0.06 84.76

denovo28 0.00 0.00 0.04 0.31 0.06 84.82

denovo75 0.00 0.00 0.04 0.31 0.06 84.88

GQ274045 0.00 0.00 0.04 0.32 0.06 84.94

denovo692 0.00 0.00 0.03 0.40 0.06 85.00

denovo1899 0.00 0.00 0.03 0.30 0.06 85.05

denovo1093 0.00 0.00 0.03 0.22 0.06 85.11

denovo1517 0.00 0.00 0.03 0.32 0.06 85.17

denovo690 0.00 0.00 0.03 0.32 0.06 85.22

denovo1131 0.00 0.00 0.03 0.22 0.06 85.28

denovo707 0.00 0.00 0.03 0.32 0.06 85.34

denovo492 0.00 0.00 0.03 0.39 0.06 85.39

denovo1581 0.00 0.00 0.03 0.39 0.06 85.45

denovo1303 0.00 0.00 0.03 0.30 0.06 85.50

denovo990 0.00 0.00 0.03 0.22 0.05 85.56

denovo43 0.00 0.00 0.03 0.38 0.05 85.61

denovo1555 0.00 0.00 0.03 0.30 0.05 85.67

denovo1688 0.00 0.00 0.03 0.22 0.05 85.72

denovo1944 0.00 0.00 0.03 0.38 0.05 85.77

AB176169 0.00 0.00 0.03 0.27 0.05 85.83

denovo777 0.00 0.00 0.03 0.39 0.05 85.88

denovo1827 0.00 0.00 0.03 0.40 0.05 85.93

FQ660217 0.00 0.00 0.03 0.33 0.05 85.99

denovo850 0.00 0.00 0.03 0.40 0.05 86.04

denovo1291 0.00 0.00 0.03 0.30 0.05 86.09

denovo1754 0.00 0.00 0.03 0.32 0.05 86.14

denovo24 0.00 0.00 0.03 0.32 0.05 86.19

denovo521 0.00 0.00 0.03 0.22 0.05 86.24

HQ588359 0.00 0.00 0.03 0.22 0.05 86.29

denovo210 0.00 0.00 0.03 0.32 0.05 86.34

denovo659 0.00 0.00 0.03 0.37 0.05 86.40

denovo1875 0.00 0.00 0.03 0.22 0.05 86.45

denovo272 0.00 0.00 0.03 0.30 0.05 86.50

denovo608 0.00 0.00 0.03 0.32 0.05 86.55

DQ889935 0.00 0.00 0.03 0.33 0.05 86.59

denovo1759 0.00 0.00 0.03 0.39 0.05 86.64

denovo682 0.00 0.00 0.03 0.32 0.05 86.69

denovo853 0.00 0.00 0.03 0.41 0.05 86.74

denovo872 0.00 0.00 0.03 0.32 0.05 86.79

denovo1147 0.00 0.00 0.03 0.28 0.05 86.84

FJ999591 0.00 0.00 0.03 0.28 0.05 86.89

denovo1489 0.00 0.00 0.03 0.32 0.05 86.93

denovo898 0.00 0.00 0.03 0.32 0.05 86.98

denovo1082 0.00 0.00 0.03 0.23 0.05 87.03

denovo1441 0.00 0.00 0.03 0.40 0.05 87.08

denovo1103 0.00 0.00 0.03 0.30 0.05 87.12

denovo648 0.00 0.00 0.03 0.32 0.04 87.17

denovo1753 0.00 0.00 0.03 0.22 0.04 87.21

denovo147 0.00 0.00 0.03 0.22 0.04 87.26

denovo199 0.00 0.00 0.03 0.22 0.04 87.30

EU539909 0.00 0.00 0.03 0.22 0.04 87.35

EF076171 0.00 0.00 0.03 0.31 0.04 87.39

denovo1959 0.00 0.00 0.03 0.31 0.04 87.44

denovo1523 0.00 0.00 0.03 0.22 0.04 87.48

AJ863291 0.00 0.00 0.03 0.22 0.04 87.52

denovo1274 0.00 0.00 0.03 0.22 0.04 87.57

denovo1383 0.00 0.00 0.03 0.22 0.04 87.61

denovo225 0.00 0.00 0.03 0.22 0.04 87.65

denovo887 0.00 0.00 0.03 0.32 0.04 87.70

denovo1873 0.00 0.00 0.03 0.22 0.04 87.74

denovo34 0.00 0.00 0.03 0.22 0.04 87.78

denovo114 0.00 0.00 0.03 0.32 0.04 87.83

denovo1240 0.00 0.00 0.03 0.32 0.04 87.87

FJ557790 0.00 0.00 0.03 0.32 0.04 87.91

denovo183 0.00 0.00 0.03 0.22 0.04 87.96

DQ446109 0.00 0.00 0.03 0.22 0.04 88.00

HQ326257 0.00 0.00 0.03 0.22 0.04 88.04

FJ826409 0.00 0.00 0.03 0.31 0.04 88.08

denovo1740 0.00 0.00 0.03 0.31 0.04 88.12

denovo1476 0.00 0.00 0.03 0.32 0.04 88.17

denovo543 0.00 0.00 0.02 0.31 0.04 88.21

EF153415 0.00 0.00 0.02 0.31 0.04 88.25

denovo818 0.00 0.00 0.02 0.22 0.04 88.29

denovo929 0.00 0.00 0.02 0.32 0.04 88.33

denovo1047 0.00 0.00 0.02 0.32 0.04 88.37

denovo243 0.00 0.00 0.02 0.32 0.04 88.41

FJ675001 0.00 0.00 0.02 0.23 0.04 88.45

denovo660 0.00 0.00 0.02 0.31 0.04 88.48

EU794092 0.00 0.00 0.02 0.32 0.04 88.52

FJ820465 0.00 0.00 0.02 0.23 0.04 88.56

denovo1299 0.00 0.00 0.02 0.23 0.04 88.60

denovo787 0.00 0.00 0.02 0.23 0.04 88.64

denovo918 0.00 0.00 0.02 0.23 0.04 88.68

FJ946588 0.00 0.00 0.02 0.23 0.04 88.72

denovo1850 0.00 0.00 0.02 0.22 0.04 88.76

denovo1905 0.00 0.00 0.02 0.32 0.04 88.79

EF123323 0.00 0.00 0.02 0.32 0.04 88.83

denovo769 0.00 0.00 0.02 0.33 0.04 88.87

denovo1139 0.00 0.00 0.02 0.22 0.04 88.91

denovo1443 0.00 0.00 0.02 0.22 0.04 88.94

denovo1911 0.00 0.00 0.02 0.22 0.04 88.98

denovo1937 0.00 0.00 0.02 0.22 0.04 89.02

denovo1998 0.00 0.00 0.02 0.22 0.04 89.05

denovo565 0.00 0.00 0.02 0.22 0.04 89.09

denovo921 0.00 0.00 0.02 0.22 0.04 89.13

denovo859 0.00 0.00 0.02 0.32 0.04 89.16

denovo819 0.00 0.00 0.02 0.22 0.04 89.20

denovo1237 0.00 0.00 0.02 0.22 0.04 89.23

denovo1856 0.00 0.00 0.02 0.22 0.04 89.27

denovo236 0.00 0.00 0.02 0.22 0.04 89.30

denovo94 0.00 0.00 0.02 0.22 0.04 89.34

denovo480 0.00 0.00 0.02 0.32 0.04 89.37

EU536078 0.00 0.00 0.02 0.31 0.03 89.41

FJ848403 0.00 0.00 0.02 0.32 0.03 89.44

denovo1328 0.00 0.00 0.02 0.32 0.03 89.48

denovo1742 0.00 0.00 0.02 0.32 0.03 89.51

denovo556 0.00 0.00 0.02 0.32 0.03 89.55

denovo1301 0.00 0.00 0.02 0.32 0.03 89.58

denovo194 0.00 0.00 0.02 0.32 0.03 89.61

AAYC01000009 0.00 0.00 0.02 0.22 0.03 89.65

denovo1589 0.00 0.00 0.02 0.32 0.03 89.68

denovo1756 0.00 0.00 0.02 0.32 0.03 89.71

denovo904 0.00 0.00 0.02 0.32 0.03 89.75

denovo237 0.00 0.00 0.02 0.32 0.03 89.78

JN544140 0.00 0.00 0.02 0.33 0.03 89.81

AJ621576 0.00 0.00 0.02 0.33 0.03 89.84

denovo1403 0.00 0.00 0.02 0.32 0.03 89.87

EU287413 0.00 0.00 0.02 0.31 0.03 89.91

denovo1037 0.00 0.00 0.02 0.22 0.03 89.94

denovo1116 0.00 0.00 0.02 0.22 0.03 89.97

denovo1275 0.00 0.00 0.02 0.22 0.03 90.00

Groups Corticium & Petrosia

Average dissimilarity = 63.83

Group Corticium Group Petrosia

Species Av.Abund Av.Abund Av.Diss Diss/SD Contrib% Cum.%

denovo1749 0.63 0.89 8.71 0.83 13.65 13.65

JN579972 0.13 0.00 3.11 0.41 4.87 18.52

GQ118701 0.12 0.10 3.10 0.93 4.85 23.37

denovo75 0.12 0.00 2.31 0.38 3.63 26.99

FJ516885 0.06 0.00 1.78 0.25 2.79 29.79

denovo1026 0.15 0.01 1.16 0.73 1.82 31.60

JN030555 0.00 0.05 1.16 0.24 1.82 33.42

denovo1484 0.14 0.02 1.09 0.85 1.71 35.13

JF937422 0.13 0.06 1.09 0.78 1.71 36.84

denovo1146 0.03 0.03 0.79 1.14 1.23 38.07

FJ751910 0.04 0.00 0.79 0.23 1.23 39.30

denovo189 0.03 0.02 0.77 1.00 1.21 40.52

denovo1017 0.16 0.01 0.75 0.48 1.18 41.69

JF802167 0.03 0.00 0.72 0.26 1.13 42.83

AY739689 0.03 0.00 0.59 0.22 0.92 43.75

HQ245988 0.03 0.00 0.57 0.25 0.90 44.64

denovo109 0.02 0.01 0.56 0.93 0.87 45.52

denovo507 0.02 0.02 0.54 0.94 0.85 46.37

denovo1189 0.02 0.02 0.53 1.03 0.83 47.20

denovo1249 0.02 0.02 0.53 1.22 0.83 48.03

EU846601 0.03 0.00 0.49 0.22 0.77 48.80

AF286480 0.02 0.00 0.47 0.69 0.74 49.54

denovo114 0.11 0.00 0.45 0.39 0.70 50.24

AY914065 0.01 0.03 0.44 0.76 0.69 50.93

denovo838 0.11 0.01 0.44 0.40 0.68 51.61

denovo756 0.11 0.00 0.42 0.38 0.65 52.27

denovo1873 0.02 0.00 0.41 0.42 0.65 52.92

denovo1022 0.02 0.01 0.40 0.58 0.62 53.54

denovo1905 0.15 0.00 0.38 0.24 0.60 54.14

AB176201 0.01 0.01 0.35 0.65 0.55 54.70

denovo1743 0.11 0.00 0.34 0.30 0.53 55.22

denovo204 0.01 0.00 0.33 0.85 0.52 55.74

EU035954 0.01 0.02 0.33 0.53 0.52 56.26

EU935300 0.01 0.02 0.32 0.72 0.51 56.77

denovo1476 0.11 0.00 0.32 0.29 0.50 57.27

denovo1791 0.11 0.00 0.32 0.29 0.50 57.77

GU940713 0.01 0.01 0.32 0.52 0.50 58.26

EU536078 0.01 0.00 0.31 0.69 0.49 58.75

DQ889875 0.00 0.02 0.29 0.48 0.46 59.21

denovo141 0.01 0.01 0.29 0.90 0.46 59.67

AY631057 0.02 0.00 0.29 0.27 0.45 60.12

denovo324 0.11 0.00 0.28 0.25 0.44 60.56

AM992178 0.01 0.00 0.28 0.23 0.44 61.00

denovo1322 0.11 0.00 0.27 0.24 0.43 61.43

denovo854 0.01 0.01 0.27 0.60 0.42 61.85

denovo792 0.01 0.00 0.26 0.40 0.40 62.25

denovo1565 0.11 0.00 0.25 0.23 0.40 62.65

denovo1640 0.11 0.00 0.25 0.23 0.40 63.05

denovo1785 0.11 0.00 0.25 0.23 0.40 63.45

GQ385289 0.11 0.00 0.25 0.23 0.40 63.84

denovo828 0.00 0.01 0.25 0.36 0.40 64.24

denovo545 0.01 0.01 0.23 1.16 0.36 64.60

denovo1273 0.01 0.01 0.23 0.55 0.36 64.97

AM935808 0.00 0.02 0.23 0.22 0.36 65.33

denovo1145 0.01 0.01 0.21 1.07 0.34 65.66

GU118526 0.00 0.02 0.21 0.36 0.33 65.99

denovo415 0.00 0.01 0.20 0.97 0.32 66.31

denovo427 0.01 0.01 0.20 1.02 0.31 66.62

AF170746 0.00 0.01 0.20 0.47 0.31 66.93

FQ659744 0.00 0.01 0.19 0.22 0.30 67.23

denovo1848 0.00 0.01 0.19 0.22 0.29 67.53

HQ323455 0.01 0.00 0.18 0.25 0.29 67.81

denovo430 0.00 0.01 0.18 0.95 0.28 68.09

denovo1547 0.00 0.02 0.18 0.43 0.28 68.37

HQ616267 0.00 0.01 0.18 0.77 0.28 68.65

denovo1577 0.01 0.00 0.18 0.56 0.27 68.92

JF802166 0.01 0.00 0.17 0.22 0.27 69.19

denovo1162 0.00 0.01 0.17 0.43 0.26 69.45

AF252321 0.01 0.00 0.16 0.28 0.26 69.71

denovo1374 0.00 0.00 0.16 0.39 0.25 69.96

HQ721418 0.00 0.01 0.16 0.57 0.25 70.21

HM108471 0.00 0.01 0.16 0.77 0.25 70.45

denovo1753 0.01 0.00 0.16 0.30 0.24 70.70

denovo1306 0.01 0.00 0.16 0.31 0.24 70.94

denovo293 0.00 0.00 0.15 0.30 0.24 71.18

FJ957855 0.01 0.00 0.15 0.22 0.24 71.42

X97093 0.00 0.01 0.15 0.52 0.23 71.65

denovo550 0.01 0.00 0.14 0.22 0.22 71.87

GU179554 0.00 0.01 0.14 0.54 0.21 72.08

denovo1491 0.01 0.00 0.13 0.25 0.21 72.29

denovo607 0.01 0.00 0.13 0.44 0.20 72.49

FJ957708 0.01 0.00 0.13 0.22 0.20 72.69

JN868782 0.00 0.00 0.13 0.62 0.20 72.89

denovo1263 0.01 0.00 0.12 0.22 0.19 73.09

AJ347026 0.00 0.01 0.12 0.39 0.19 73.28

denovo40 0.01 0.00 0.12 0.29 0.19 73.47

FJ849072 0.01 0.00 0.12 0.22 0.18 73.65

denovo1996 0.01 0.00 0.12 0.22 0.18 73.83

denovo776 0.00 0.01 0.12 0.42 0.18 74.01

denovo1182 0.00 0.01 0.12 0.43 0.18 74.20

AB477015 0.00 0.01 0.12 0.58 0.18 74.38

FJ169195 0.00 0.00 0.11 0.56 0.18 74.55

GQ903461 0.01 0.00 0.11 0.35 0.17 74.72

EU160577 0.00 0.01 0.11 0.52 0.17 74.88

FJ418924 0.00 0.00 0.10 0.33 0.16 75.05

denovo1193 0.00 0.00 0.10 0.22 0.16 75.21

AM709702 0.00 0.01 0.10 0.41 0.16 75.37

denovo6 0.00 0.00 0.10 0.34 0.16 75.53

denovo1632 0.00 0.00 0.10 0.73 0.16 75.68

GU118575 0.00 0.01 0.10 0.44 0.15 75.83

denovo1789 0.00 0.00 0.09 0.43 0.14 75.97

denovo1579 0.00 0.00 0.09 0.73 0.14 76.11

denovo61 0.00 0.01 0.09 0.50 0.14 76.25

denovo1478 0.00 0.00 0.09 0.22 0.14 76.39

denovo547 0.00 0.00 0.09 0.40 0.14 76.52

FJ826409 0.00 0.00 0.09 0.41 0.13 76.66

denovo1250 0.00 0.00 0.08 0.49 0.13 76.79

GU553034 0.00 0.00 0.08 0.25 0.13 76.92

denovo499 0.00 0.00 0.08 0.32 0.13 77.05

denovo916 0.00 0.00 0.08 0.22 0.13 77.18

denovo1570 0.00 0.00 0.08 0.22 0.13 77.31

denovo2056 0.00 0.00 0.08 0.22 0.13 77.44

denovo757 0.00 0.00 0.08 0.22 0.13 77.57

JQ032050 0.00 0.00 0.08 0.22 0.13 77.70

GQ921403 0.00 0.00 0.08 0.22 0.12 77.82

denovo1592 0.00 0.00 0.08 0.22 0.12 77.94

denovo325 0.00 0.00 0.08 0.22 0.12 78.06

denovo696 0.00 0.00 0.07 0.22 0.12 78.18

denovo1757 0.00 0.00 0.07 0.36 0.12 78.29

denovo98 0.00 0.01 0.07 0.39 0.12 78.41

EU803928 0.00 0.01 0.07 0.37 0.12 78.52

denovo1284 0.00 0.01 0.07 0.40 0.12 78.64

U81990 0.00 0.00 0.07 0.53 0.11 78.76

GU118606 0.00 0.01 0.07 0.47 0.11 78.87

denovo249 0.00 0.00 0.07 0.58 0.11 78.98

GU118690 0.00 0.01 0.07 0.40 0.11 79.10

AB294294 0.00 0.00 0.07 0.28 0.11 79.21

denovo43 0.00 0.00 0.07 0.62 0.11 79.32

denovo574 0.00 0.01 0.07 0.34 0.11 79.42

GQ433928 0.00 0.00 0.06 0.26 0.10 79.52

denovo1619 0.00 0.00 0.06 0.42 0.10 79.62

EF076171 0.00 0.00 0.06 0.50 0.10 79.72

DQ889881 0.00 0.01 0.06 0.41 0.10 79.82

denovo2003 0.00 0.01 0.06 0.45 0.10 79.92

FJ892785 0.00 0.00 0.06 0.46 0.10 80.02

GU305772 0.00 0.00 0.06 0.53 0.10 80.11

JF925031 0.00 0.00 0.06 0.50 0.10 80.21

denovo1479 0.00 0.00 0.06 0.37 0.09 80.30

FJ900573 0.00 0.01 0.06 0.41 0.09 80.39

denovo917 0.00 0.00 0.06 0.27 0.09 80.48

denovo412 0.00 0.00 0.06 0.35 0.09 80.57

denovo679 0.00 0.00 0.06 0.27 0.09 80.66

GQ263306 0.00 0.00 0.06 0.40 0.09 80.75

denovo777 0.00 0.00 0.05 0.42 0.09 80.83

FJ820465 0.00 0.00 0.05 0.31 0.09 80.92

JF937433 0.00 0.00 0.05 0.36 0.08 81.00

denovo853 0.00 0.00 0.05 0.42 0.08 81.08

denovo744 0.00 0.00 0.05 0.37 0.08 81.17

JF712668 0.00 0.00 0.05 0.45 0.08 81.25

denovo528 0.00 0.00 0.05 0.25 0.08 81.32

denovo1696 0.00 0.00 0.05 0.28 0.08 81.40

denovo305 0.00 0.00 0.05 0.39 0.08 81.48

denovo788 0.00 0.00 0.05 0.22 0.08 81.56

HM595366 0.00 0.00 0.05 0.46 0.08 81.64

denovo1888 0.00 0.00 0.05 0.36 0.08 81.71

denovo1720 0.00 0.00 0.05 0.29 0.08 81.79

AJ621576 0.00 0.00 0.05 0.45 0.07 81.86

HQ326290 0.00 0.00 0.05 0.30 0.07 81.94

denovo719 0.00 0.00 0.05 0.37 0.07 82.01

FJ999591 0.00 0.00 0.04 0.35 0.07 82.08

FJ557790 0.00 0.00 0.04 0.29 0.07 82.15

denovo88 0.00 0.00 0.04 0.22 0.07 82.22

denovo1199 0.00 0.00 0.04 0.22 0.07 82.28

denovo1298 0.00 0.00 0.04 0.22 0.07 82.35

denovo1830 0.00 0.00 0.04 0.22 0.07 82.42

denovo1892 0.00 0.00 0.04 0.22 0.07 82.49

denovo481 0.00 0.00 0.04 0.22 0.07 82.56

denovo694 0.00 0.00 0.04 0.22 0.07 82.63

denovo946 0.00 0.00 0.04 0.22 0.07 82.70

DQ833490 0.00 0.00 0.04 0.22 0.07 82.76

HM243844 0.00 0.00 0.04 0.22 0.07 82.83

denovo1590 0.00 0.00 0.04 0.40 0.07 82.90

denovo1526 0.00 0.00 0.04 0.22 0.07 82.97

denovo1842 0.00 0.00 0.04 0.31 0.07 83.03

AJ240982 0.00 0.00 0.04 0.22 0.07 83.10

denovo1701 0.00 0.00 0.04 0.46 0.07 83.17

denovo815 0.00 0.00 0.04 0.38 0.07 83.23

DQ860060 0.00 0.00 0.04 0.32 0.07 83.30

denovo1705 0.00 0.00 0.04 0.22 0.07 83.36

denovo613 0.00 0.00 0.04 0.46 0.06 83.43

denovo611 0.00 0.00 0.04 0.22 0.06 83.49

denovo605 0.00 0.00 0.04 0.38 0.06 83.55

denovo143 0.00 0.00 0.04 0.29 0.06 83.62

denovo1962 0.00 0.00 0.04 0.46 0.06 83.68

denovo860 0.00 0.00 0.04 0.22 0.06 83.74

denovo165 0.00 0.00 0.04 0.29 0.06 83.80

denovo1244 0.00 0.00 0.04 0.22 0.06 83.86

FJ802385 0.00 0.00 0.04 0.33 0.06 83.92

denovo457 0.00 0.00 0.04 0.29 0.06 83.99

denovo1062 0.00 0.00 0.04 0.45 0.06 84.05

denovo1889 0.00 0.00 0.04 0.41 0.06 84.10

denovo1637 0.00 0.00 0.04 0.38 0.06 84.16

DQ396132 0.00 0.00 0.04 0.31 0.06 84.22

denovo28 0.00 0.00 0.04 0.31 0.06 84.28

EF076172 0.00 0.00 0.04 0.39 0.06 84.34

FJ529262 0.00 0.00 0.04 0.38 0.06 84.39

HQ672216 0.00 0.00 0.04 0.31 0.06 84.45

denovo1093 0.00 0.00 0.04 0.22 0.06 84.51

denovo1131 0.00 0.00 0.04 0.22 0.06 84.56

denovo1118 0.00 0.00 0.04 0.30 0.06 84.62

denovo320 0.00 0.00 0.04 0.45 0.06 84.67

denovo812 0.00 0.00 0.04 0.36 0.06 84.73

EF153415 0.00 0.00 0.04 0.38 0.05 84.78

denovo1561 0.00 0.00 0.03 0.30 0.05 84.84

denovo707 0.00 0.00 0.03 0.32 0.05 84.89

denovo1688 0.00 0.00 0.03 0.22 0.05 84.94

denovo1303 0.00 0.00 0.03 0.30 0.05 85.00

FJ957596 0.00 0.00 0.03 0.39 0.05 85.05

FJ497578 0.00 0.00 0.03 0.30 0.05 85.10

EU817491 0.00 0.00 0.03 0.30 0.05 85.16

denovo1555 0.00 0.00 0.03 0.30 0.05 85.21

JN411267 0.00 0.00 0.03 0.32 0.05 85.26

FJ624884 0.00 0.00 0.03 0.26 0.05 85.31

AJ583166 0.00 0.00 0.03 0.26 0.05 85.36

FQ660217 0.00 0.00 0.03 0.32 0.05 85.42

JF808978 0.00 0.00 0.03 0.39 0.05 85.47

denovo1690 0.00 0.00 0.03 0.32 0.05 85.52

HQ616309 0.00 0.00 0.03 0.32 0.05 85.57

AY959053 0.00 0.00 0.03 0.33 0.05 85.62

denovo1452 0.00 0.00 0.03 0.40 0.05 85.67

AJ867671 0.00 0.00 0.03 0.38 0.05 85.72

HQ588359 0.00 0.00 0.03 0.22 0.05 85.78

denovo1641 0.00 0.00 0.03 0.22 0.05 85.83

denovo521 0.00 0.00 0.03 0.22 0.05 85.88

denovo659 0.00 0.00 0.03 0.37 0.05 85.93

FJ808721 0.00 0.00 0.03 0.39 0.05 85.98

denovo60 0.00 0.00 0.03 0.31 0.05 86.03

denovo771 0.00 0.00 0.03 0.31 0.05 86.07

denovo1403 0.00 0.00 0.03 0.39 0.05 86.12

denovo871 0.00 0.00 0.03 0.31 0.05 86.17

DQ889935 0.00 0.00 0.03 0.32 0.05 86.22

denovo1823 0.00 0.00 0.03 0.39 0.05 86.26

denovo148 0.00 0.00 0.03 0.22 0.05 86.31

denovo1147 0.00 0.00 0.03 0.27 0.05 86.36

denovo1917 0.00 0.00 0.03 0.32 0.05 86.40

denovo1265 0.00 0.00 0.03 0.32 0.05 86.45

denovo1082 0.00 0.00 0.03 0.22 0.05 86.49

denovo1401 0.00 0.00 0.03 0.37 0.05 86.54

denovo1085 0.00 0.00 0.03 0.31 0.04 86.58

denovo1839 0.00 0.00 0.03 0.22 0.04 86.63

denovo1346 0.00 0.00 0.03 0.39 0.04 86.67

denovo1095 0.00 0.00 0.03 0.22 0.04 86.72

HE574879 0.00 0.00 0.03 0.22 0.04 86.76

AJ863291 0.00 0.00 0.03 0.22 0.04 86.80

denovo1274 0.00 0.00 0.03 0.22 0.04 86.85

denovo887 0.00 0.00 0.03 0.32 0.04 86.89

denovo34 0.00 0.00 0.03 0.22 0.04 86.94

denovo308 0.00 0.00 0.03 0.32 0.04 86.98

denovo636 0.00 0.00 0.03 0.30 0.04 87.02

denovo1150 0.00 0.00 0.03 0.22 0.04 87.07

denovo183 0.00 0.00 0.03 0.22 0.04 87.11

HQ326257 0.00 0.00 0.03 0.22 0.04 87.15

denovo624 0.00 0.00 0.03 0.22 0.04 87.19

DQ129268 0.00 0.00 0.03 0.31 0.04 87.23

denovo358 0.00 0.00 0.03 0.22 0.04 87.28

denovo708 0.00 0.00 0.03 0.22 0.04 87.32

denovo1240 0.00 0.00 0.03 0.31 0.04 87.36

denovo1881 0.00 0.00 0.03 0.30 0.04 87.40

denovo818 0.00 0.00 0.03 0.22 0.04 87.44

denovo797 0.00 0.00 0.03 0.31 0.04 87.48

GU563744 0.00 0.00 0.03 0.31 0.04 87.52

DQ298271 0.00 0.00 0.03 0.32 0.04 87.56

denovo1226 0.00 0.00 0.03 0.22 0.04 87.60

denovo543 0.00 0.00 0.03 0.30 0.04 87.64

denovo1581 0.00 0.00 0.02 0.32 0.04 87.68

denovo1415 0.00 0.00 0.02 0.30 0.04 87.72

denovo346 0.00 0.00 0.02 0.32 0.04 87.75

FN666870 0.00 0.00 0.02 0.22 0.04 87.79

denovo244 0.00 0.00 0.02 0.31 0.04 87.83

denovo423 0.00 0.00 0.02 0.22 0.04 87.87

denovo1299 0.00 0.00 0.02 0.22 0.04 87.90

denovo787 0.00 0.00 0.02 0.22 0.04 87.94

denovo918 0.00 0.00 0.02 0.22 0.04 87.98

FJ946588 0.00 0.00 0.02 0.22 0.04 88.02

FJ675001 0.00 0.00 0.02 0.23 0.04 88.05

EU794092 0.00 0.00 0.02 0.32 0.04 88.09

denovo1774 0.00 0.00 0.02 0.30 0.04 88.13

denovo862 0.00 0.00 0.02 0.32 0.04 88.17

denovo859 0.00 0.00 0.02 0.32 0.04 88.20

denovo656 0.00 0.00 0.02 0.32 0.04 88.24

denovo1938 0.00 0.00 0.02 0.22 0.04 88.27

denovo1291 0.00 0.00 0.02 0.22 0.04 88.31

denovo1820 0.00 0.00 0.02 0.31 0.04 88.35

denovo846 0.00 0.00 0.02 0.22 0.04 88.38

denovo819 0.00 0.00 0.02 0.22 0.04 88.42

HQ143274 0.00 0.00 0.02 0.22 0.04 88.45

denovo1855 0.00 0.00 0.02 0.22 0.04 88.49

denovo66 0.00 0.00 0.02 0.22 0.04 88.53

denovo769 0.00 0.00 0.02 0.32 0.04 88.56

denovo480 0.00 0.00 0.02 0.32 0.04 88.60

denovo1653 0.00 0.00 0.02 0.31 0.03 88.63

denovo32 0.00 0.00 0.02 0.31 0.03 88.66

FJ203135 0.00 0.00 0.02 0.39 0.03 88.70

FJ848403 0.00 0.00 0.02 0.31 0.03 88.73

HQ753432 0.00 0.00 0.02 0.31 0.03 88.77

denovo631 0.00 0.00 0.02 0.22 0.03 88.80

denovo184 0.00 0.00 0.02 0.31 0.03 88.83

HQ118340 0.00 0.00 0.02 0.29 0.03 88.87

denovo1589 0.00 0.00 0.02 0.31 0.03 88.90

denovo1801 0.00 0.00 0.02 0.32 0.03 88.93

denovo1292 0.00 0.00 0.02 0.32 0.03 88.97

denovo709 0.00 0.00 0.02 0.32 0.03 89.00

denovo1390 0.00 0.00 0.02 0.32 0.03 89.03

denovo571 0.00 0.00 0.02 0.22 0.03 89.07

FJ214667 0.00 0.00 0.02 0.22 0.03 89.10

denovo904 0.00 0.00 0.02 0.31 0.03 89.13

denovo1684 0.00 0.00 0.02 0.32 0.03 89.16

denovo1918 0.00 0.00 0.02 0.32 0.03 89.20

EU768401 0.00 0.00 0.02 0.32 0.03 89.23

FN794273 0.00 0.00 0.02 0.32 0.03 89.26

denovo16 0.00 0.00 0.02 0.31 0.03 89.29

denovo1395 0.00 0.00 0.02 0.32 0.03 89.33

denovo552 0.00 0.00 0.02 0.31 0.03 89.36

denovo104 0.00 0.00 0.02 0.31 0.03 89.39

denovo1775 0.00 0.00 0.02 0.22 0.03 89.42

denovo260 0.00 0.00 0.02 0.22 0.03 89.45

denovo865 0.00 0.00 0.02 0.32 0.03 89.48

denovo801 0.00 0.00 0.02 0.22 0.03 89.51

denovo997 0.00 0.00 0.02 0.22 0.03 89.54

CU919411 0.00 0.00 0.02 0.22 0.03 89.57

HM584318 0.00 0.00 0.02 0.22 0.03 89.60

denovo158 0.00 0.00 0.02 0.31 0.03 89.64

JN544140 0.00 0.00 0.02 0.33 0.03 89.67

GQ249358 0.00 0.00 0.02 0.32 0.03 89.70

denovo804 0.00 0.00 0.02 0.30 0.03 89.73

denovo432 0.00 0.00 0.02 0.30 0.03 89.76

denovo1936 0.00 0.00 0.02 0.22 0.03 89.79

denovo899 0.00 0.00 0.02 0.32 0.03 89.82

denovo285 0.00 0.00 0.02 0.22 0.03 89.85

JN397763 0.00 0.00 0.02 0.31 0.03 89.88

denovo385 0.00 0.00 0.02 0.22 0.03 89.90

denovo728 0.00 0.00 0.02 0.22 0.03 89.93

denovo673 0.00 0.00 0.02 0.22 0.03 89.96

denovo1457 0.00 0.00 0.02 0.32 0.03 89.99

GU118588 0.00 0.00 0.02 0.23 0.03 90.02
